# Genetic effects of sequence-conserved enhancer-like elements on human complex traits

**DOI:** 10.1186/s13059-023-03142-1

**Published:** 2024-01-02

**Authors:** Xiang Zhu, Shining Ma, Wing Hung Wong

**Affiliations:** 1 Department of Statistics, The Pennsylvania State University, 326 Thomas Building, University Park, 16802 PA USA; 2 Huck Institutes of the Life Sciences, The Pennsylvania State University, 201 Huck Life Sciences Building, University Park, 16802 PA USA; 3 Department of Statistics, Stanford University, 390 Jane Stanford Way, Stanford, 94305 CA USA; 4 Department of Biomedical Data Science, Stanford University School of Medicine, 1265 Welch Road MC5464, Stanford, 94305 CA USA

**Keywords:** Genome-wide association study, Sequence conservation, Enhancer, Tissue specificity, Heritability, Fine mapping, Gene prioritization, Data integration

## Abstract

**Background:**

The vast majority of findings from human genome-wide association studies (GWAS) map to non-coding sequences, complicating their mechanistic interpretations and clinical translations. Non-coding sequences that are evolutionarily conserved and biochemically active could offer clues to the mechanisms underpinning GWAS discoveries. However, genetic effects of such sequences have not been systematically examined across a wide range of human tissues and traits, hampering progress to fully understand regulatory causes of human complex traits.

**Results:**

Here we develop a simple yet effective strategy to identify functional elements exhibiting high levels of human-mouse sequence conservation and enhancer-like biochemical activity, which scales well to 313 epigenomic datasets across 106 human tissues and cell types. Combined with 468 GWAS of European (EUR) and East Asian (EAS) ancestries, these elements show tissue-specific enrichments of heritability and causal variants for many traits, which are significantly stronger than enrichments based on enhancers without sequence conservation. These elements also help prioritize candidate genes that are functionally relevant to body mass index (BMI) and schizophrenia but were not reported in previous GWAS with large sample sizes.

**Conclusions:**

Our findings provide a comprehensive assessment of how sequence-conserved enhancer-like elements affect complex traits in diverse tissues and demonstrate a generalizable strategy of integrating evolutionary and biochemical data to elucidate human disease genetics.

**Supplementary Information:**

The online version contains supplementary material available at 10.1186/s13059-023-03142-1.

## Background

To delineate functional elements [1] in the human genome, two major complementary approaches have been developed. The first approach [2, 3] searches for conserved sequences that remain unchanged over evolution across species (e.g., human and mouse), assuming that mutations therein typically reduce fitness and are thus under negative selection. The second approach [4, 5] omits evolutionary conservation and instead identifies sequences of biochemical activity through epigenomic profiling, such as ChIP-seq of H3K27ac to mark active enhancers [6]. The evolutionary approach has been empowered by genome sequencing and assembly for a growing number of species [7, 8]. In parallel, genome-wide catalogs of diverse biochemical marks have been generated in hundreds of human cell types and tissues [9, 10]. Collectively, the two approaches have enhanced our understanding of human genome function, especially for the vast non-coding regions that do not encode protein sequences.

Non-coding elements detected by the evolutionary and biochemical approaches are particularly relevant to human disease genetics, as GWAS often implicate non-coding regions [11]. Non-coding elements with either evolutionary [12] or biochemical [10] signatures can help prioritize functional variants and yield mechanistic insights at GWAS loci. Additionally, genomic regions marked by each of the two approaches explain a much larger proportion of heritability for complex traits [13] than one would expect by the region sizes. Despite the progress, both approaches have limitations to define regulatory elements [1, 6], and thus each approach alone cannot fully inform the regulatory causes of heritable traits.

Inspired by previous efforts of combining evolutionary and biochemical approaches to prioritize regulatory sequences in mammalian genomes [14, 15], recent studies have adopted this concept to interpret non-coding variation underlying human traits. Integrating evolutionary and biochemical data has proven effective in quantifying the fitness consequences of genetic variants [16, 17], outperforming methods that utilize a single data type [18, 19]. Besides the totality of phenotypic consequences (fitness), the integrative approach is also useful to elucidate the genetics of a specific trait. DNase I hypersensitivity sites in human fetal brains intersected with evolutionarily conserved sequences display a significant excess of de novo mutations concentrated exclusively in neurodevelopmental disorders [20]. Human orthologues of H3K27ac [21] and open chromatin [22–24] peaks in mouse brains are enriched for GWAS signals of many brain-related traits in specific brain cell types. H3K27ac peaks in human livers [25] show significantly stronger heritability enrichments across 41 complex traits [26] when restricted to peaks with sequence age older than the marsupial-placental split [27] or peaks with conserved H3K27ac signal in mammals [25]. Although promising, these efforts only assessed regions with both evolutionary and biochemical signatures for a limited set of tissues and traits. The genetic effects of these regions on a wide range of traits across diverse tissues remain largely unknown, impeding our ability to understand tissue-specific regulation of hereditary traits [10].

Here, we revisit the classical idea of exploiting human-mouse sequence conservation to locate functional elements in the human genome [28, 29], which has informed many large-scale initiatives [9, 30]. Building on this simple but profound idea, we develop a human-mouse comparison method to identify human enhancer-like elements that display both sequence conservation and biochemical activity. We apply the method to 313 epigenomic datasets across 106 tissues and cell types and employ the identified elements to analyze 468 GWAS of EUR and EAS ancestries. These elements not only show strong tissue-specific enrichments of heritability and causal variants for a wide range of traits but also nominate previously undescribed effector genes for BMI and schizophrenia, revealing additional biological and clinical insights. Overall, we present a scalable and effective strategy to annotate the human genome with complementary lines of evolutionary and biochemical evidence, and demonstrate its utility systematically across a host of tissues and traits.

## Results

### Human-mouse comparisons identify conserved enhancer-like sequences

**Fig. 1:**
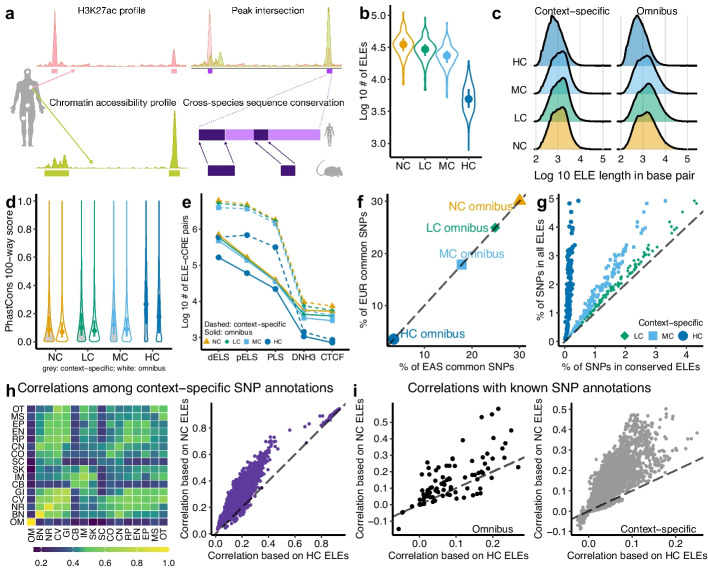
Identify and characterize sequence-conserved human enhancer-like elements. **a** Schematic of identifying ELEs (NC) and subsets with low (LC), moderate (MC) and high (HC) levels of sequence conservation. **b** Numbers of context-specific ELEs across 105 contexts. **c**, **d** Length (**c**) and evolutionary conservation score (**d**) distributions for NC, LC, MC, and HC ELEs. For **b** and **d**, each point denotes a median and each line denotes an interquartile range. **e** Numbers of overlapping pairs between ELEs and ENCODE cCREs. dELS and pELS, distal and proximal enhancer-like signature, respectively. PLS, promoter-like signature. DNH3, signature marked by DNase and H3K4me3. **f** Percentages of EUR and EAS common SNPs inside omnibus ELEs. **g** Percentages of EUR common SNPs inside context-specific ELEs. Each point denotes a context. **h** The heatmap shows the maximum correlations of SNP annotations based on NC context-specific ELEs from two context groups. BN, bone. CB, cancer (blood). CO, cancer (other). CV, cardiovascular. CN, connective. EN, endocrine. EP, epithelial. GI, gastrointestinal. IM, immune. MS, muscle. NR, neural. OM, omnibus. OT, other tissues (kidney, liver, lung). RP, reproductive. SK, skin. SC, stem cell. The scatter plot shows the pairwise correlations of SNP annotations based on NC and HC context-specific ELEs, where each point denotes a pair of contexts. **i** Correlations between a known SNP annotation and an ELE-based SNP annotation. Each point denotes a pair of an ELE-based SNP annotation and one of the 96 known SNP annotations. For **h–i**, the correlation of two binary annotations is quantified by Cramér’s *V* (from 0 to 1; $V=0$: no correlation; $V=1$: complete correlation). The correlation of a binary and a quantitative annotation is quantified by Pearson’s *R* (from –1 to 1; $R=0$: no correlation; $|R|=1$: complete correlation). For **f**–**i**, dashed lines have intercept 0 and slope 1

We developed a simple method to identify putative human enhancers that exhibit sequence conservation in the mouse genome (Fig. 1a; Methods). Given a human tissue or cell type (henceforth “context”), we first used its H3K27ac profile to empirically determine putative enhancers across the human genome [5] and then intersected them with accessible chromatin regions identified in the same context. Since biochemical activity is not necessarily a definitive proof of enhancer function [1, 6], we cautiously termed these regions marked by H3K27ac and chromatin accessibility signals “enhancer-like elements” (ELEs). Finally, we identified sequence-conserved ELEs by comparing human ELEs with the mouse genome [28, 29]. We specified the level of sequence conservation as the minimum proportion of bases mapped to gapless aligned blocks in the mouse genome (minMatch), with larger values indicating higher conservation levels. Under this definition, a higher level of sequence conservation would pose a more stringent threshold for a given ELE to be classified as conserved. Consequently, the collection of conserved ELEs became a smaller subset of all ELEs as the conservation level increased.

We applied this method to context-matched H3K27ac and chromatin accessibility profiles across 106 contexts in humans (Additional file 1: Table S1). First, we generated all ELEs for each context without considering sequence conservation in the mouse genome, denoted as NC ELEs. The median NC ELE count per context is 35,065 (range: 8309–112,362). Across all NC ELEs, the median length is 1119 bp (range: 100–194,631 bp). We then created three non-disjoint sets of conserved ELEs in the same context by setting the sequence conservation level as 0.1, 0.5, and 0.9, denoted as lowly (LC), moderately (MC) and highly conserved (HC) ELEs, respectively. As expected, there are fewer HC ELEs than NC ELEs in the same context (median decrease: 29,296; Fig. 1b), and HC ELEs are shorter than NC ELEs (median decrease: 450 bp; Fig. 1c).

For each set (NC, LC, MC, HC), we further aggregated all context-specific ELEs across 106 contexts and merged overlapping segments into non-overlapping ones to produce an “omnibus” version (Additional file 2: Fig. S1a), resulting in 338,743 unique NC, 247,504 LC, 222,160 MC, and 132,717 HC omnibus ELEs (Additional file 2: Fig. S1b). The increased element counts are expected, because omnibus ELEs accumulate ELEs from diverse contexts, most of which are unique to a single context. Of all HC omnibus ELEs, 52.1% are indeed HC ELEs from one context (Additional file 2: Fig. S1c), and 57.6% consist of HC ELEs from one context group (Additional file 2: Fig. S1d).

To assess their evolutionary and biochemical relevance, we overlapped the four sets of ELEs with the 100-vertebrate phastCons scores [18] (Fig. 1d) and the human candidate cis-regulatory elements (cCREs) from ENCODE [9] (Fig. 1e; Methods). Reassuringly, HC omnibus ELEs are more evolutionarily conserved than NC omnibus ELEs (median increase: 0.11; one-sided Wilcoxon $P<2.2\times 10^{-308}$). More than 90% of the omnibus ELE-cCRE pairs contain enhancer-like signatures irrespective of sequence conservation. We observed similar patterns for context-specific ELEs. Together, the results show that all ELEs are often biochemically active, while conserved ELEs display both evolutionary and biochemical signals.

While the vast majority of ELEs map to promoter-distal regions that are more than 2 kb away from a transcriptional start site (TSS), a small fraction of ELEs may lie in close proximity to promoters. Specifically, approximately 1–3% of the omnibus ELEs fall within 200 bp of an annotated TSS, and roughly 4–9% of the omnibus ELE-cCRE pairs possess promoter-like signatures that are marked by H3K4me3 strength and TSS proximity (Methods). These ELEs may denote TSS-proximal enhancers [31], enhancer-like promoters [32], or other functional elements around canonical promoters [9]. Compared to NC ELEs, HC ELEs tend to overlap with a TSS more frequently (3.1% versus 0.9%; Additional file 2: Fig. S1e) and capture more promoter-like signatures (8.8% versus 4.3%; Fig. 1e). These observations recapitulate previous findings that promoters are more conserved across species than enhancers [25, 30].

To assess their regulatory functions, we tested motif enrichments in ELEs (Methods). Despite their smaller coverage of the human genome compared with all ELEs (Fig. 1b, c), conserved ELEs contain many significantly enriched motifs (Additional file 1: Table S2). Specifically, we identified 151 unique motifs in HC ELEs showing strong enrichments ($\ge 2$-fold and $P\le 1.0\times 10^{-12}$) in at least one context against the GC-matched random background. To avoid confounding caused by context specificity, we repeated this analysis with the background being all ELEs in the same context and identified enrichments for 186 unique motifs. Some enriched motifs are relevant to the context from which HC ELEs are derived. For example, HC ELEs derived from neural progenitors are enriched for a DBX2 motif (2.09-fold, $P=1.0\times 10^{-100}$), consistent with the brain-specific mRNA expression of DBX2 and its regulatory role in age-related neurogenic decline [33]. The motif enrichments confirm high concentrations of regulatory sequences in conserved ELEs, forming a basis to interpret non-coding variation.

To capture their genetic variation, we mapped ELEs to biallelic autosomal single-nucleotide polymorphisms (SNPs) with minor allele frequency above 0.05 (henceforth “common SNPs”) in the EUR and EAS populations [34]. In total, $30.1\%$ of 5,961,159 EUR common SNPs lie within NC, $24.9\%$ within LC, $17.9\%$ within MC, and $3.6\%$ within HC omnibus ELEs. These percentages are the same up to three decimal places for 5,469,053 EAS common SNPs (Fig. 1f). We also mapped context-specific ELEs to common SNPs and observed a similar trend of ELEs with a higher level of sequence conservation covering fewer common SNPs (Fig. 1g), consistent with the patterns of ELE count (Fig. 1b) and length (Fig. 1c).

To investigate context specificity, we examined correlations between the annotations of common SNPs (henceforth “SNP annotations”) for all ELEs in all context pairs (Fig. 1h; Additional file 1: Table S3). Reassuringly, correlations are generally stronger (average increase: 0.12; Wilcoxon one-sided $P=4.4\times 10^{-35}$) when ELEs belong to the same context group (e.g., ascending aorta and tibial artery, Cramér’s $V=0.73$) than when they are in distant groups (e.g., neural progenitor and smooth muscle cell, $V=0.08$). The correlations are high in different but related contexts, such as immune and blood cancer groups (both rich in immune cells, $V=0.53$) and cardiovascular and gastrointestinal groups (both rich in muscle and connective cells, $V=0.69$). Conserved ELEs produce concordant results for the same context pairs (Pearson’s $R=0.87-0.99$; Fig. 1h), showing that conserved ELEs preserve the context specificity of all ELEs.

We also correlated 96 known annotations [26] with ELE-based SNP annotations (Fig. 1i; Additional file 1: Table S4). The NC omnibus ELE annotation is weakly correlated with most of the 96 annotations (median $V=9.9\times 10^{-2}$; $2.2\times 10^{-4}\le V\le 0.58$). The strongest correlation is for a context-merged H3K27ac annotation [13], consistent with the construction of NC omnibus ELEs (Fig. 1a; Additional file 2: Fig. S1a). Compared to the NC omnibus ELE annotation, the HC omnibus ELE annotation is far less correlated with existing SNP annotations (median $V=6.5\times 10^{-2}$; $9.0\times 10^{-5}\le V\le 0.27$; Wilcoxon one-sided $P=2.3\times 10^{-9}$). The strongest correlation is for an evolutionary constraint annotation [19], consistent with the role of sequence conservation in the identification of HC ELEs. We observed even weaker correlations between context-specific ELE annotations and existing SNP annotations (NC: $1.1\times 10^{-7}\le V\le 0.50$; HC: $1.3\times 10^{-6}\le V\le 0.25$). The identified ELEs, especially HC ELEs, yield new SNP annotations that are not strongly correlated with existing SNP annotations, suggesting their potential to capture additional genetic signals of complex traits.

### HC ELEs explain heritability independent of known SNP annotations

Enrichments of common SNP heritability for complex traits have been shown in various SNP annotations [13, 26, 35]. Because of their potential functions (Fig. 1d, e) and weak correlations with existing SNP annotations (Fig. 1i), we hypothesized that SNP annotations based on ELEs could be enriched in heritability, independent of contributions from known SNP annotations. To test this hypothesis, we used S-LDSC [13] to analyze 468 GWAS (Additional file 1: Table S5) and 4 SNP annotations of omnibus ELEs from this study (Methods; Additional file 1: Table S6). For each GWAS, we first applied S-LDSC to the annotation of NC omnibus ELEs while conditioning on 96 previous SNP annotations [26]. We then analyzed each of three annotations of conserved (LC, MC, HC) omnibus ELEs with S-LDSC while conditioning on the NC omnibus ELEs and 96 previous annotations. For each GWAS and annotation, we summarized the S-LDSC analysis by (1) heritability enrichment ($\ge 1$), defined as the proportion of heritability explained by SNPs in the annotation divided by the proportion of SNPs in the annotation, and (2) standardized effect size ($\tau ^\star \ge 0$), defined as the proportionate change in per-SNP heritability associated with a 1-standard deviation increase of the annotation, conditioned on all other annotations. The heritability enrichment indicates the marginal effect of an annotation, while $\tau ^\star$ indicates the unique effect of an annotation.

**Fig. 2:**
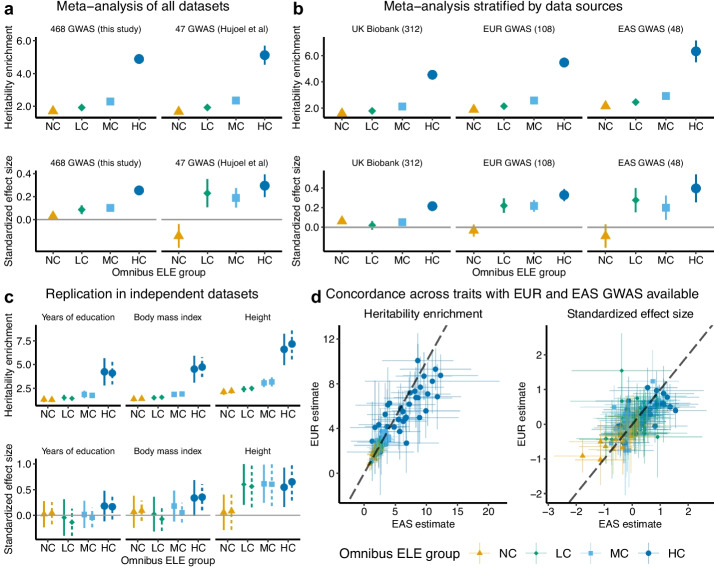
Quantify common SNP heritability enrichments for sequence-conserved omnibus ELEs. We show heritability enrichment and standardized effect size ($\tau ^\star$) estimates for 4 SNP annotations based on all omnibus ELEs without considering sequence conservation (NC) and the subsets with varying levels of sequence conservation (LC: low; MC: moderate; HC: high). The estimates for NC omnibus ELEs are conditional on 96 known annotations. The estimates for LC, MC, and HC omnibus ELEs are conditional on NC omnibus ELEs and 96 known annotations. **a** Estimates meta-analyzed across 468 GWAS datasets curated for this study and a previously defined set of 47 independent GWAS datasets, respectively. **b** Estimates meta-analyzed across 312 UK Biobank, 108 EUR, and 48 EAS GWAS datasets, respectively. Numerical results for **a** and **b** are available in Additional file 1: Table S7. **c** Comparison of heritability enrichment and $\tau ^\star$ estimates based on 2 independent EUR GWAS for 3 representative traits. **d** Heritability enrichment and $\tau ^\star$ estimates for 4 omnibus ELE annotations across 34 traits that each has EUR and EAS GWAS available. Numerical results for **c** and **d** are available in Additional file 1: Table S6. For **a**–**d**, each point denotes an estimate and each error bar denotes $\pm 1.96$ SE. For **d**, dashed lines have intercept 0 and slope 1

In the meta-analysis across all 468 GWAS, we observed a significant heritability enrichment for NC omnibus ELEs (1.71-fold, one-sided $P=6.9\times 10^{-263}$), consistent with previous findings [13, 26]. We also found NC omnibus ELEs uniquely informative for per-SNP heritability conditional on 96 known annotations, as quantified by $\tau ^\star =0.029$ (one-sided $P=1.7\times 10^{-2}$). When analyzing conserved omnibus ELEs, we identified a rising signal strength along the sequence conservation level (Fig. 2a). Specifically, we obtained 4.88-fold heritability enrichment ($P=5.4\times 10^{-325}$) for HC omnibus ELEs, compared to 2.29-fold ($P=3.0\times 10^{-277}$) for MC and 1.93-fold ($P=1.7\times 10^{-341}$) for LC. Conditional on NC omnibus ELEs and 96 previous annotations, we estimated $\tau ^\star =0.255$ ($P=4.8\times 10^{-65}$) for HC omnibus ELEs, compared to $\tau ^\star =0.101$ ($P=2.8\times 10^{-13}$) for MC and $\tau ^\star =0.086$ ($P=1.8\times 10^{-6}$) for LC. Together, the results demonstrate a significant effect of HC omnibus ELEs on heritability, which is not explained by NC omnibus ELEs or known SNP annotations.

As a sensitivity analysis, we restricted the meta-analysis to a previously described set [26] of 47 independent datasets (Fig. 2a). Despite the larger standard errors (SEs) caused by the fewer datasets meta-analyzed, we obtained similar heritability enrichments for all four annotations ($P\ge 0.44$ for difference). We also estimated similar $\tau ^\star$ for HC omnibus ELEs ($P=0.43$ for difference), highlighting the robustness of our results based on this SNP annotation.

We further meta-analyzed results (Additional file 1: Table S7) stratified by study populations (Fig. 2b) and trait categories (Additional file 2: Fig. S2), reaching two conclusions consistent with the full analysis (Fig. 2a). First, HC omnibus ELEs have stronger heritability enrichments than NC omnibus ELEs ($2.12-6.13$ fold increase). Second, HC omnibus ELEs have significantly positive effect sizes (median $\tau ^\star =0.33$ and $P=3.6 \times 10^{-5}$ across 28 strata) conditional on NC omnibus ELEs and 96 known annotations. Across three populations, the meta-analysis of 48 EAS GWAS produced the strongest enrichment for HC omnibus ELEs (6.33-fold, $P=1.6\times 10^{-27}$; $\tau ^\star =0.396$, $P=2.2\times 10^{-8}$). Within EUR GWAS, the meta-analysis of 8 cardiovascular traits produced the strongest enrichment (8.13-fold, $P=6.2\times 10^{-6}$; $\tau ^\star =0.682$, $P=6.8\times 10^{-8}$). Within UK Biobank, the meta-analysis of 19 medication use traits produced the strongest enrichment (6.04-fold, $P=6.6\times 10^{-11}$; $\tau ^\star =0.525$, $P=2.2\times 10^{-13}$). Despite quantitative differences, the qualitative finding remains the same: HC ELEs are more informative than NC ELEs for heritability enrichment.

To assess replicability, we examined the S-LDSC results of 13 traits that each had two independent EUR GWAS with comparable sample sizes. For all four annotations of the omnibus ELEs, we obtained similar results between two independent datasets of the same trait ($P>0.05/13$ for difference; Fig. 2c).

To evaluate the transferability of our findings across populations, we compared the results of 34 traits that each had EUR and EAS GWAS available. Across annotations and traits, we obtained concordant estimates between EUR and EAS (Fig. 2d; heritability enrichment: $R=0.91$, $P=7.6\times 10^{-52}$; $\tau ^\star$: $R=0.63$, $P=1.9\times 10^{-16}$). Furthermore, we found no evidence of population heterogeneity for the same annotation and trait ($P>0.05/34$ for difference). The estimates tend to be smaller in EUR than EAS (heritability enrichment: $\text {slope}=0.93$, $\text {SE}=0.052$; $\tau ^\star$: $\text {slope}=0.86$, $\text {SE}=0.068$; Methods), which is consistent with our meta-analysis stratified by populations (Fig. 2b) as well as a recent EAS-EUR comparison across 29 traits and 100 regulatory annotations [35]. Overall, the results not only indicate comparable heritability enrichments for EUR and EAS in all omnibus ELEs regardless of sequence conservation, but also show consistently stronger enrichments in HC than in NC omnibus ELEs for both populations.

### HC ELEs show context-specific heritability enrichments

Having established the strong heritability enrichment for HC omnibus ELEs, we next assessed context-dependent enrichments for HC ELEs (Additional file 1: Table S8). Specifically, we analyzed the annotation of HC context-specific ELEs from each context against each GWAS with S-LDSC, while conditioning on all ELEs in the same context and 96 previous annotations (Methods). We quantified the significance of context-specific enrichment by a one-sided *P*-value that tests $\tau ^\star >0$, controlling for effects of all other annotations.

**Fig. 3:**
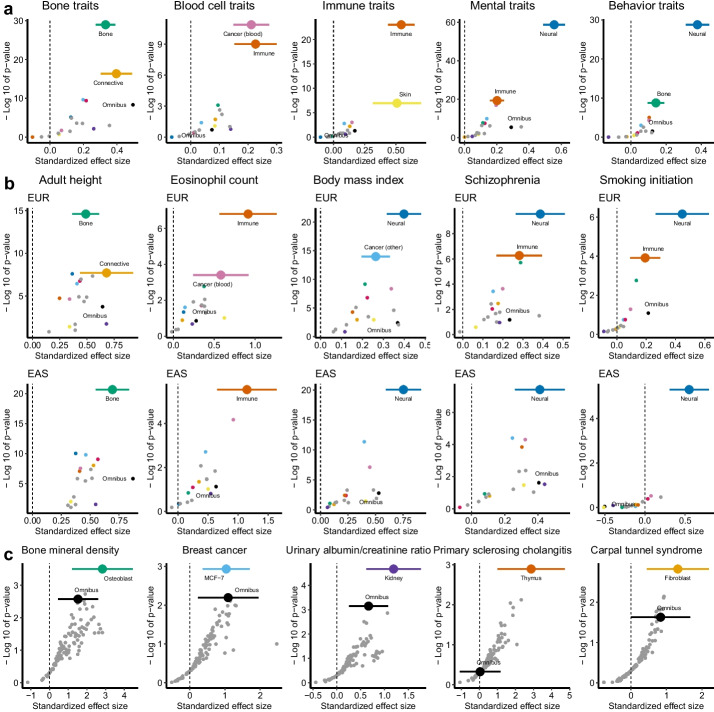
Assess common SNP heritability enrichments for HC context-specific ELEs. For HC context-specific ELEs from each of the 105 contexts, we compute the standardized effect size ($\tau ^\star$) estimate and *P*-value for testing $\tau ^\star >0$ conditional on all ELEs in the same context and 96 known annotations. **a** Estimates meta-analyzed within each of the 17 context groups (Additional file 1: Table S1) for 5 groups of related traits in EUR GWAS. Additional results are shown in Additional file 2: Fig. S3. **b** Estimates meta-analyzed within each of the 17 context groups for 5 traits that have both EUR and EAS GWAS available. Numerical results for **a** and **b** are available in Additional file 1: Table S9. **c** Estimates of 105 individual contexts for 5 exemplary traits. Numerical results are available in Additional file 1: Table S8. For **a**–**c**, each point denotes an estimate and each error bar denotes $\pm 1.96$ SE. The color legend is provided in Additional file 2: Fig. S1j

We first meta-analyzed results across groups of related traits and contexts. For many trait groups, we observed top-ranked enrichments in HC ELEs derived from contexts highly relevant to the traits (Fig. 3a; Additional file 1: Table S9; Additional file 2: Fig. S3). HC ELEs derived from the nervous system show strong enrichments for mental disorders ($\tau ^\star =0.556$, $P=1.7\times 10^{-59}$) and a wide range of traits related to behavior ($\tau ^\star =0.451$, $P=1.4\times 10^{-125}$), sleep ($\tau ^\star =0.422$, $P=2.5\times 10^{-76}$), reproduction ($\tau ^\star =0.367$, $P=1.1\times 10^{-8}$), and diet ($\tau ^\star =0.359$, $P=1.3\times 10^{-162}$). HC ELEs derived from the immune system show strong enrichments for immune diseases ($\tau ^\star =0.543$, $P=9.4\times 10^{-24}$) and blood cell traits ($\tau ^\star =0.226$, $P=1.0\times 10^{-9}$). Other examples include bone for bone traits ($\tau ^\star =0.332$, $P=1.1\times 10^{-29}$), connective tissue for early growth traits ($\tau ^\star =0.456$, $P=7.2\times 10^{-11}$), and kidney for kidney traits ($\tau ^\star =0.998$, $P=4.2\times 10^{-9}$). The significantly positive $\tau ^\star$ estimates indicate that HC ELEs provide additional information about heritability conditional on ELEs in the same context. Furthermore, top enrichments of HC context-specific ELEs are consistently stronger than enrichments of HC omnibus ELEs based on the same GWAS, recapitulating the tissue selectivity of heritable traits [10, 13].

We also observed context-dependent enrichments in the meta-analysis of related contexts for a single trait. Specifically, for 34 traits with both EUR and EAS GWAS available, we often identified the strongest enrichments of HC ELEs from the same context group (Fig. 3b). For example, HC ELEs derived from the nervous system show the strongest enrichments in EUR and EAS for BMI, schizophrenia, and smoking initiation. Other examples are bone for adult height and immune system for eosinophil count. Across all contexts and traits, we obtained concordant estimates of $\tau ^\star$ between EUR and EAS ($R=0.53$, $P=1.4\times 10^{-253}$) and found no evidence of population heterogeneity ($P>0.05/(34\times 105)$ for difference). As in the omnibus results (Fig. 2d), we estimated smaller $\tau ^\star$ for HC context-specific ELEs in EUR than in EAS ($\text {slope}=0.69$, $\text {SE}=0.018$). Overall, the results showcase the transferability of heritability enrichments for HC context-specific ELEs across populations.

Lastly, we examined individual contexts for a given trait (Fig. 3c). As expected, individual enrichments are weaker than meta-analyzed enrichments, but the top-ranked ones still inform trait-relevant contexts. Some top-ranked enrichments are straightforward to interpret, such as kidney for urine albumin-creatinine ratio, osteoblast for bone mineral density, and MCF-7 for breast cancer. Some top-ranked enrichments are less direct but functionally relevant nonetheless. HC ELEs derived from the thymus (where T cells mature) show the strongest enrichment among 105 contexts for primary sclerosing cholangitis (PSC), followed by HC ELEs derived from T cells. This adds to the considerable evidence linking T cells to the pathogenesis of PSC [36]. HC ELEs derived from fibroblasts (connective tissue) show the strongest enrichment for carpal tunnel syndrome (CTS), consistent with the fibrosis of subsynovial connective tissue in CTS patients [37].

### HC ELEs capture more heritability than H3K27ac-conserved ELEs

Our identification of HC ELEs differs from previous work of detecting human enhancers aligned with H3K27ac signals in other mammals [25]. To compare the two approaches, we exploited 14 contexts that each had data of human chromatin accessibility, human and mouse H3K27ac available (Additional file 1: Table S10). For each context, we created two sets of HC ELEs based on the same level of sequence conservation (minMatch = 0.9) with the mouse genome (Fig. 1a) and mouse H3K27ac peaks (Additional file 2: Fig. S4a; Methods), respectively. We then assessed heritability enrichments for the two sets of HC ELEs on the same GWAS (Additional file 1: Table S11). Though using less information, we observed stronger heritability enrichments for HC ELEs based on the mouse genome (10.9-fold, $P=5.4\times 10^{-14}$; $\tau ^\star =0.158$, $P=8.9\times 10^{-215}$) than for HC ELEs based on mouse H3K27ac peaks (8.4-fold, $P=4.9\times 10^{-15}$; $\tau ^\star =0.079$, $P=6.1\times 10^{-121}$) in the meta-analysis across GWAS and contexts (Additional file 1: Table S12). We obtained similar results when restricting to an independent set of GWAS (Additional file 2: Fig. S4b) or individual contexts (Additional file 2: Fig. S4c). Besides capturing more heritability than H3K27ac-conserved ELEs, HC ELEs do not require human-mouse H3K27ac data in the same context, thus widening applicability.

### HC ELEs harbor an excess of likely causal variants

Besides heritability enrichment, we examined fine-mapped GWAS variants in ELEs. Specifically, we intersected all omnibus ELEs (NC) and the conserved subsets (LC, MC, HC) with 515,848 fine-mapped variants [38] of 94 traits whose posterior inclusion probabilities (PIPs) were estimated by two different approaches (FINEMAP; SUSIE) independent of any SNP annotation (Methods). For each trait and annotation, we computed the fraction of fine-mapped variants inside the elements with PIPs above a given threshold. We further compared these fractions with the fraction of all fine-mapped variants that had PIPs above the same threshold in the same trait, to quantify enrichments.

**Fig. 4:**
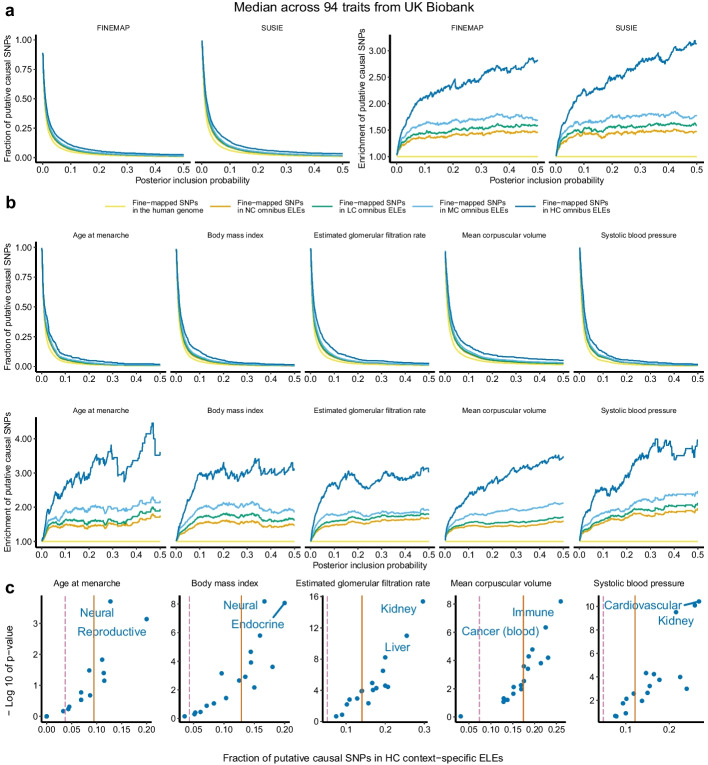
Overlap fine-mapped GWAS variants with sequence-conserved ELEs. For each of the 94 traits, we compute fractions of putative causal SNPs that fall inside the omnibus ELEs (NC) and the sequence-conserved subsets (LC, MC, HC) and then compare them against the fraction of putative causal SNPs among all fine-mapped SNPs in the genome for the trait to assess enrichments. A fine-mapped SNP is “putative causal” for a trait if this SNP has a PIP above a given threshold. **a** Median fractions and enrichments across 94 traits for 2 fine-mapping methods. **b** Individual fractions and enrichments for 5 traits based on SUSIE. Additional results are available in Additional file 1: Table S13. **c** Fractions of SNPs with SUSIE-estimated PIP $\ge 0.1$ that fall inside HC context-specific ELEs from each of the 17 context groups for 5 traits. The solid and dashed lines denote the fractions of putative causal SNPs for a given trait that fall inside HC omnibus ELEs and the whole genome, respectively. Additional results are available in Additional file 1: Table S14

Across 94 traits (Fig. 4a), we observed consistently larger fractions (median increase: $0.5-9.2\%$) and stronger enrichments (median increase: $5.6\times 10^{-3}-1.7$) of fine-mapped variants in HC than in NC omnibus ELEs, as PIP thresholds varied from 0 to 0.5. For example, across 94 traits we obtained a median 13.9% of variants with SUSIE-estimated $\text {PIP}\ge 0.1$ among the fine-mapped variants residing in HC omnibus ELEs, compared to 10.1% for MC, 9.4% for LC, 8.9% for NC, and 6.1% for the whole genome. We obtained highly concordant results between SUSIE and FINEMAP (e.g., $R=0.99$ for HC omnibus ELEs), confirming the robustness of our findings to fine-mapping methods. We also observed similar patterns in individual traits (Fig. 4b; Additional file 1: Table S13). Altogether, the results demonstrate a significant enrichment of putative causal variants in HC omnibus ELEs.

To characterize the context specificity of fine-mapping enrichments, we intersected the HC ELEs of 17 context groups (Additional file 1: Table S1) with the 515,848 fine-mapped variants (Fig. 4c; Additional file 1: Table S14). For each trait and group, we calculated the fraction of fine-mapped variants residing in HC ELEs from this context group with PIPs above 0.1. We compared this fraction with the fraction of all fine-mapped variants that had PIPs above 0.1 in the same trait, producing a one-sided binomial *P*-value to quantify enrichments. Similar to heritability enrichments (Fig. 3), context-specific enrichments of fine-mapped variants in HC ELEs highlight trait-relevant contexts. Fine-mapped variants for BMI show a stronger enrichment in HC ELEs from the neural ($16.7\%$, $P=6.6\times 10^{-9}$) and endocrine ($20.0\%$, $P=8.5\times 10^{-9}$) groups than the HC omnibus ELEs ($12.9\%$). For estimated glomerular filtration rate, fine-mapped variants are strongly enriched in HC ELEs from the kidney group ($29.6\%$, $P=4.1\times 10^{-16}$; omnibus: $14.0\%$), Other examples include immune-related HC ELEs for blood cell phenotypes ($18.8-45.0\%$, $P=6.5\times 10^{-9}-7.6\times 10^{-6}$; omnibus: $13.6-25.2\%$) and cardiovascular-related HC ELEs for blood pressure traits ($17.1-26.8\%$, $P=3.7\times 10^{-11}-8.6\times 10^{-7}$; omnibus: $10.8-12.6\%$).

### HC ELEs aid prioritization of trait-associated regulatory elements

**Fig. 5:**
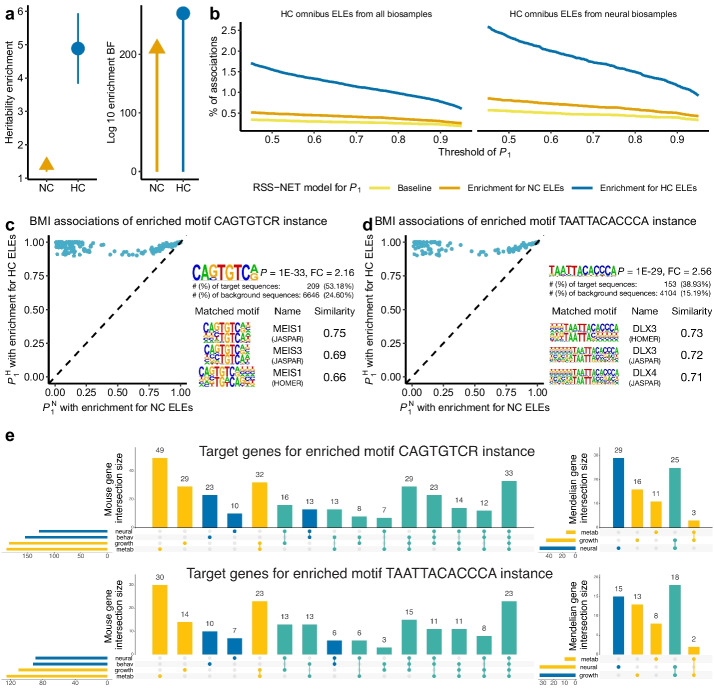
Prioritize HC ELEs for BMI. **a** Enrichments of heritability (S-LDSC) and genetic associations (RSS-NET) for all omnibus ELEs (NC) and the subset with a high level of sequence conservation (HC). **b** Percentages of HC omnibus ELEs with $P_1^\text {H}$ (enrichment model for HC ELEs), $P_1^\text {N}$ (enrichment model for NC ELEs), or $P_1^\text {B}$ (baseline model without any enrichment) above a given threshold. **c** BMI associations of 210 HC ELEs that contain instances of the enriched motif CAGTGTCR. **d** BMI associations of 154 HC ELEs that contain instances of the enriched motif TAATTACACCCA. For **c**-**d**, each point denotes a HC omnibus ELE present in neural samples, with *y*-axis and *x*-axis indicating its $P_1^\text {H}$ and $P_1^\text {N}$ respectively. FC, fold change. **e** Overlap of the putative target genes (507 for CAGTGTCR-instance and 309 for TAATTACACCCA-instance HC ELE, respectively) with genes implicated in knockout mouse phenotypes and human Mendelian traits. behav, behavioral. metab, metabolic

**Fig. 6:**
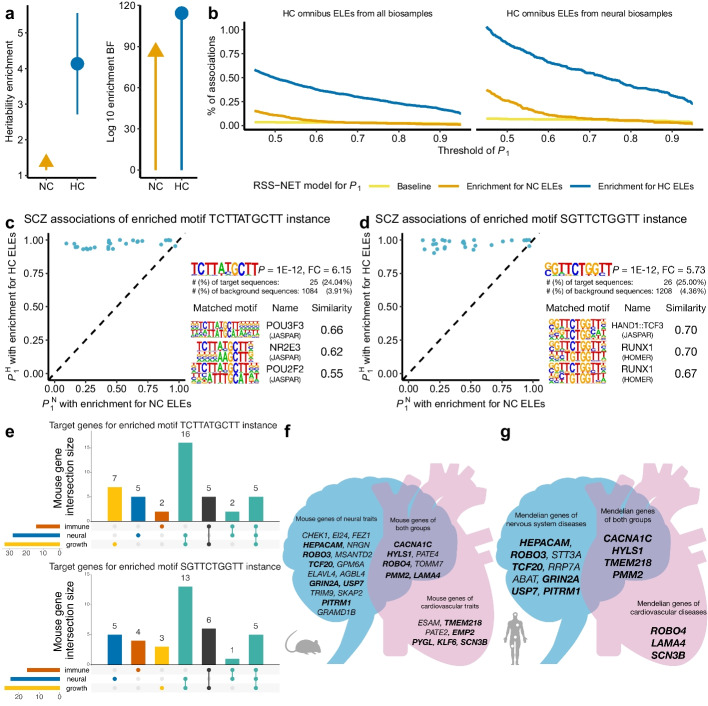
Prioritize HC ELEs for schizophrenia. Legends of **a**, **b** are the same as those in Fig. 5a, b. **c** Schizophrenia associations of 26 HC ELEs that contain instances of the enriched motif TCTTATGCTT. **d** Schizophrenia associations of 27 HC ELEs that contain instances of the enriched motif SGTTCTGGTT. For **c**, **d**, the rest is the same as Fig. 5c, d. **e** Overlap of the putative target genes (59 for TCTTATGCTT-instance and 59 for SGTTCTGGTT-instance HC ELEs) with genes implicated in knockout mouse phenotypes. **f**, **g** Putative target genes of schizophrenia-associated HC ELEs with the enriched motif SGTTCTGGTT that cause neural, cardiac, or both types of knockout mouse phenotypes (**f**) and neural, cardiac, or both types of human Mendelian traits (**g**). For **f**, **g**, genes shown in bold font are implicated in both knockout mouse phenotypes and human Mendelian traits

To prioritize trait-associated regulatory elements based on conserved ELEs, we extended RSS-NET [39], a method that simultaneously infers genetic enrichments and associations from GWAS summary statistics and genomic annotations (Methods; Additional file 2: Note S1). After validating this RSS-NET extension through simulations (Additional file 2: Note S2 and Figs. S5-8), we applied it to analyze the omnibus ELEs in the GWAS of BMI [40] (Fig. 5) and schizophrenia [41] (Fig. 6). As a sanity check, we examined enrichments produced by RSS-NET in each trait. Reassuringly, HC omnibus ELEs are more enriched in genetic associations than omnibus ELEs for both traits (Figs. 5a and 6a), mirroring the pattern of S-LDSC results (Additional file 1: Table S6). As such, we focused on the genetic associations of HC omnibus ELEs hereafter.

To quantify the genetic association between an ELE and a trait, we computed a posterior probability for each ELE that at least one SNP in this element is associated with the trait ($P_1$; Methods), assuming that HC omnibus ELEs are enriched for associations with this trait ($P_1^\text {H}$). For comparison, we also computed $P_1$ for the same element-trait pair assuming (1) no enrichment ($P_1^\text {B}$) and (2) enrichment for NC omnibus ELEs ($P_1^\text {N}$). Here, we used a significant association cutoff of $P_1^\text {H}\ge 0.9$, which yielded false positive rates less than $7.6\times 10^{-4}$ and false discovery rates less than 0.1 across all simulation scenarios (Additional file 2: Fig. S8).

The enrichment-informed $P_1^\text {H}$ increases the inferred number of genetic associations (Additional file 1: Table S15; Additional file 2: Fig. S9). Of 100,591 HC omnibus ELEs, 781 are associated with BMI at $P_1^\text {H}\ge 0.9$, compared to 304 at $P_1^\text {N}\ge 0.9$ and 229 at $P_1^\text {B}\ge 0.9$ (Fig. 5b). Similarly, 173 HC omnibus ELEs are associated with schizophrenia at $P_1^\text {H}\ge 0.9$, compared to 13 at $P_1^\text {N}\ge 0.9$ and 19 at $P_1^\text {B}\ge 0.9$ (Fig. 6b). Of 33,745 HC omnibus ELEs present in neural samples (a context highly relevant to BMI [42] and schizophrenia [43]; Fig. 3b), the same trend holds for BMI (394 at $P_1^\text {H}\ge 0.9$, 168 at $P_1^\text {N}\ge 0.9$, 132 at $P_1^\text {B}\ge 0.9$) and schizophrenia (105 at $P_1^\text {H}\ge 0.9$, 10 at $P_1^\text {N}\ge 0.9$, 14 at $P_1^\text {B}\ge 0.9$). The enhanced evidence for genetic associations (measured by $P_1^\text {H}$) is attributed to the enrichment-informed design of RSS-NET for prioritizing associations at HC ELEs. Specifically, once the enrichment of HC ELEs is identified for a trait, RSS-NET automatically increases the prior association probability and effect size for SNPs therein, which in turn increases the posterior association probability and effect size for these SNPs (Additional file 2: Note S3). The results further demonstrate the potential of our approach to identify additional regulatory elements associated with complex traits that might otherwise be missed by GWAS alone.

To assess regulatory functions of the identified associations, we searched for sequence motifs significantly enriched in a target set of trait-associated elements ($P_1^\text {H} \ge 0.9$) relative to a background set of non-associated elements ($P_1^\text {H} \le 0.1$). For both the target and background, we only used HC omnibus ELEs present in neural samples (Additional file 2: Fig. S1f-i) to minimize confounding introduced by sequence conservation or context specificity. We identified 127 and 3 enriched motifs from 394 and 105 HC ELEs associated with BMI (Additional file 1: Table S16) and schizophrenia (Additional file 1: Table S17), respectively. Furthermore, we linked trait-associated elements containing top-ranked motifs to their putative target genes using a variety of functional genomic resources (Methods; Additional file 2: Figs. S10-S14). Many linked genes are functionally and therapeutically relevant to the trait of interest (Additional file 2: Fig. S15), as reported below.

### HC ELEs inform candidate effector genes for BMI

The 394 BMI-associated HC ELEs show the strongest enrichment of a sequence motif recognized by MEIS1 (Fig. 5c), which has key roles in adipogenesis [44] and neural development [45]. Of 210 BMI-associated HC ELEs that contain the MEIS1 motif, 107 are connected to 507 putative target genes (Additional file 1: Table S18). Pathway analysis of these genes highlights multiple BMI-relevant processes, including apelin signaling, pituitary gland development and insulin secretion (Additional file 1: Table S19). Though not implicated in GWAS [40, 46], apelin and its receptors are involved in energy metabolism [47] and obesity [48].

The 394 BMI-associated HC ELEs are also strongly enriched for a DLX3 motif (Fig. 5d), which is essential for vertebrate development [49]. There are 154 BMI-associated HC ELEs containing the DLX3-binding motif, 74 of which are further linked to 309 putative target genes (Additional file 1: Table S20). These genes are enriched in multiple processes related to body weight, such as pancreas development, apelin signaling, and adipogenesis (Additional file 1: Table S21).

To assess their biological and clinical themes, we looked up the 568 unique putative target genes of BMI-associated HC ELEs that contain the MEIS1 or DLX3-binding motif in external databases (Additional file 1: Table S22; Methods). Of the 568 genes, 407 and 146 are associated with knockout mouse phenotypes and human Mendelian traits, respectively. More than half of the 407 knockout mouse genes show growth (213), metabolic (217) and neural (228) phenotypes. A considerable fraction of the 146 Mendelian genes cause diseases characterized by growth (50), metabolic (17) and neural (59) phenotypes. Many of the neural genes are also related to growth and metabolism. Of 228 genes with neural mouse phenotypes, 181 (79.4%) have growth or metabolic phenotypes. Of 59 Mendelian genes with neural manifestations, 27 (45.8%) affect growth. We identified similar patterns when analyzing putative target genes informed by MEIS1 and DLX3-binding motifs separately (Fig. 5e). The gene results, together with the neural enrichments of heritability (Fig. 3b) and fine-mapped variants (Fig. 4c), reinforce the key role of brain on body weight regulation [42].

Integrating BMI-associated HC ELEs with genes that affect mouse body weight and human monogenic obesity helps prioritize effector genes for BMI. Apart from the well-known obesity genes [42] (*LEP*, *PCSK1*, *NTRK2*), we identified several BMI effector genes that have not been reported in GWAS [40, 46] but are supported by multiple converging lines of evidence (Additional file 2: Figs. S10-S12). For example, *CDK5* is a strong candidate for BMI: *CDK5* encodes cyclin-dependent kinase 5 (Cdk5) that has diverse functions [50] in neurons, adipocytes and beta cells; Cdk5 affects obesity and diabetes through phosphorylation of PPAR$\gamma$ [51]; and a mutation in *CDK5* causes lissencephaly with multiple neurodevelopmental features [52]. *HSD11B1* is another plausible BMI effector: *HSD11B1* encodes 11$\beta$-hydroxysteroid dehydrogenase type 1 (11$\beta$-HSD1) that plays key roles in obesity and related metabolic diseases [53]; 11$\beta$-HSD1 overexpressed in adipose leads to visceral obesity and hyperphagia in mice [54]; and mutations in *HSD11B1* affect the regeneration of cortisol [55], a steroid hormone associated with obesity [56]. Furthermore, *CDK5* and *HSD11B1* are therapeutic targets of preclinical (L-751250) and Phase 1 (AZD8329) drugs for obesity, respectively, and our results provide genetic support for early stage development (Additional file 2: Fig. S15).

### HC ELEs inform candidate effector genes for schizophrenia

The 105 schizophrenia-associated HC ELEs show strong enrichments of motifs recognized by POU3F3 (Fig. 6c) and HAND1::TCF3 complex (Fig. 6d), all of which are relevant to schizophrenia. POU3F3 is essential for cerebral cortex development [57]. TCF3 regulates neocortical development through Wnt-$\beta$-catenin signaling [58]. HAND1 is critical for placenta development [59], which has been associated with the genetic risk of schizophrenia [60].

We identified 26 and 27 schizophrenia-associated HC ELEs containing POU3F3 and HAND1::TCF3-binding motifs respectively, further suggesting 86 unique putative target genes (Additional file 1: Tables S23-S25). Of the 31 genes related to Mendelian diseases, 19 (61.3%) have neural indications. Among 59 genes with knockout mouse phenotypes available, many have immune (22), growth (43), and neural (38) phenotypes. Of 38 neural genes, 11 (28.9%) and 29 (76.3%) have immune and growth phenotypes in knockout mice, respectively. We observed similar neural-immune and neural-growth overlaps when analyzing POU3F3 and HAND1::TCF3 target genes separately (Fig. 6e). These findings recapitulate the roles of immunity [61] and early development [43] in the etiology of schizophrenia.

Many putative target genes of schizophrenia-associated HC ELEs with the HAND1::TCF3-binding motif cause both neural and cardiac knockout mouse phenotypes (Fig. 6f) and human Mendelian traits (Fig. 6g), likely due to the key role of HAND1 in heart development [59]. Our results highlight three genes (*CACNA1C*, *HYLS1*, *PMM2*) with neural-cardiac roles. *CACNA1C* has been repeatedly identified in GWAS of schizophrenia [41] and causes arrhythmia associated with autism [62]. Both *HYLS1* and *PMM2* have not been implicated in GWAS (Additional file 1: Table S26), but their neural-cardiac roles are relevant to schizophrenia. *HYLS1* encodes hydrolethalus syndrome protein 1, which regulates the biogenesis and signaling of cilia [63]. Cilia are antenna-like organelles with essential roles in cerebral cortical [64] and cardiac [65] development. A mutation in *HYLS1* causes hydrolethalus syndrome [66] characterized by developmental defects of the fetal brain and heart. Mutations in *PMM2* cause a congenital disorder of glycosylation [67] with neurological and cardiac manifestations [68]. Glycosylation has been linked to cardiovascular [69] and neuroinflammatory [70] diseases, as well as schizophrenia [71]. In sum, HAND1::TCF3 target genes with neural-cardiac roles provide a means to elucidate the genetic causes of comorbidity between schizophrenia and cardiovascular diseases [72].

## Discussion

We present a simple and scalable strategy to identify human enhancer-like elements that are highly conserved in the mouse genome (HC ELEs) for 106 tissues and cell types. Across 468 GWAS of EUR and EAS ancestries, we demonstrate that HC ELEs harbor a significant excess of genetic signals for human complex traits, as measured by common SNP heritability and fine-mapped variants. We further show that HC ELEs capture these signals independent of existing SNP annotations, therefore providing a unique interpretation of non-coding variation in complex traits.

Integrating HC ELEs with GWAS and gene regulatory networks further helps pinpoint previously undescribed but functionally relevant genes for BMI (e.g., *CDK5*, *HSD11B1*) and schizophrenia (e.g., *HYLS1*, *PMM2*). Despite convergent evidence supporting their roles in the biology of BMI and schizophrenia, these genes prioritized by our approach were not identified by the same GWAS data used in our analysis, nor by the updated GWAS with much larger sample sizes, nor by the multi-omics integrative analysis of these GWAS data at the time of this study [40, 41, 46, 73, 74]. Identifying these genes post-GWAS presents two challenges. First, although *CDK5* and *HSD11B1* are within the loci (± 500 kb) of known GWAS hits of BMI (rs2907948, $P=1.3\times 10^{-13}$; rs12140373, $P=7.7\times 10^{-11}$), these loci contain many protein-coding genes (28 and 10, respectively), thus complicating the nomination of likely causal genes. Our approach helps address this challenge by shortlisting genes on the same regulatory circuit as HC ELEs that are strongly associated with the GWAS trait ($P_1^\text {H} \ge 0.9$), based on the premise that enhancers affect a trait through their downstream genes [38]. Second, *HYLS1* and *PMM2* are more than 950 kb away from any GWAS hits of schizophrenia ($P\le 5\times 10^{-8}$), rendering their discoveries through standard GWAS or integrative strategies difficult. Our approach helps address this challenge by first identifying trait-associated HC ELEs and then linking them to genes that are far away from GWAS signals via long-range enhancer-gene connections. As high-quality enhancer-gene maps are becoming available [38, 75], enhancer-centric approaches like ours will prioritize effector genes for complex traits beyond GWAS.

Our findings have several other important implications for the genetic architecture of complex traits. First, compared to all ELEs, HC ELEs display fewer sequence changes across species but stronger enrichments of trait heritability and causal variants, supporting the model of negative selection on genetic variants to affect complex traits [13, 26, 41]. Second, HC ELEs capture consistent signals between EUR and EAS ancestries, highlighting the potential of cross-species methods like ours to improve the transferability of genetic findings across human populations [35]. Third, though imperfectly conserved between humans and mice, HC ELEs retain regulatory functions to affect complex traits in a tissue-specific manner, corroborating the functional robustness of ultraconserved enhancers to mutations [76]. Fourth, HC ELEs highlight human sequences with high similarity in the mouse genome, suggesting a path to test human GWAS discoveries in mice [77].

While the idea of combining evolutionary and biochemical data has proven broadly useful [14–17, 25], our study demonstrates several key strengths of implementing this idea specifically for multi-omics integrative analysis in GWAS. First, we use the human-mouse sequence comparison [28, 29] to locate functional elements for hundreds of tissues and cell types, whereas existing studies often examine a single tissue [21–26]. The scalability of our method to many tissues enables the interpretation of GWAS findings through tissue-specific gene regulation [10]. Second, unlike many studies that use either H3K27ac [21, 25, 26] or chromatin accessibility [22–24] alone to mark functional elements, we integrate both types of epigenomic profiles to refine these elements. Third, we focus firmly on functional sequences in the human genome, despite the comparison of human and mouse sequences. This approach contrasts with many studies [21–24] that focus on human orthologues of functional sequences in the mouse genome, bypassing the issue that the orthologues may not be functional in humans [1, 6]. Last but not least, we assess conservation of genome sequences rather than H3K27ac signals between species [25, 26]. This choice not only yields significantly stronger heritability enrichments, but also eliminates the need for cross-species H3K27ac profiling in the same tissue, making our method more widely applicable.

Although tested on the mouse genome only, our pairwise comparison to identify HC ELEs is straightforward to implement more broadly for other species, as high-quality genomes are becoming available for many species [7, 8]. That said, we caution that the pairwise approach might fall short in evolutionarily related species, such as humans and primates, due to the paucity of cross-species sequence variation. In such case, phylogenetic modeling of multiple species may be worth pursuing [3, 18]. Another limitation is that sequence comparisons omit enhancers that are functionally conserved but nonorthologous at the sequence level [6, 78]. In such case, integrative modeling across functions and species may help [24, 79]. Despite the limitations, our simple method provides a useful benchmark for sophisticated models.

Currently, HC ELEs are based on the bulk sequencing of H3K27ac and chromatin accessibility profiles, likely missing detailed cellular processes in which regulatory variants affect complex traits. Identifying cellularly resolved HC ELEs will be enabled by the emerging single-cell epigenomic data. Indeed, single-cell atlases of chromatin accessibility have been recently established for many human tissues [80], and single-cell H3K27ac measurements will likely be available for diverse tissues soon with the advent of new technologies [81]. Besides the single-cell extension, other data such as gene expression [39, 82], chromatin conformation [83, 84], and CRISPR screening [38, 75] may need to be incorporated to capture the multifaceted nature of enhancers [6]. Altogether, fine-tuning HC ELEs alongside advances in genomic technologies and resources will markedly increase resolution and accuracy.

## Conclusions

Our findings, together with recent studies by others [8, 20–22, 26, 85, 86], emphasize the importance of combining evolutionary and biochemical evidence to understand the regulatory basis of heritable human traits. This integrative idea has been well documented and increasingly appreciated, but it remains under-exploited in depth and at scale. This work represents a comprehensive effort and a major step forward to close this gap.

## Methods

### Reference genomes

We used the GRCh37 (hg19, human) and GRCm38 (mm10, mouse) genome assemblies throughout this study. We converted data based on GRCh38 (hg38) to GRCh37 using liftOver [87] with the default setting and the minimum ratio of bases that must remap (minMatch) being 0.95.

### Human epigenomes

We collected genome-wide sequencing data of 142 H3K27ac (ChIP-seq) and 171 chromatin accessibility (DNase-seq, ATAC-seq) profiles across 106 contexts (Additional file 1: Table S1). We followed the ENCODE data standards and processing pipelines [9] to identify H3K27ac and accessible chromatin peaks in each context.

### Sequence-conserved ELEs

To identify NC ELEs without considering sequence conservation between the human and mouse genomes for each of the 106 contexts, we intersected H3K27ac and accessible chromatin peaks in this context using BEDTools [88] (version 2.27.1). To identify LC, MC, and HC ELEs with low, moderate, and high levels of human-mouse sequence conservation for each context, we searched for segments of NC ELEs that are conserved in the mouse genome using liftOver with the default setting and minMatch as 0.1, 0.5, and 0.9, respectively. Each ELE defines an interval on the human genome, indicated by the chromosome, start and end positions. Since an increased level of sequence conservation imposes a more stringent criterion for an ELE to be deemed as conserved, HC ELEs are a subset of MC ELEs, MC ELEs are a subset of LC ELEs, and LC ELEs are a subset of NC ELEs in a given context. Hence, these sets of ELE are not disjoint for each context. The source code implementing the methods, as well as all resulting ELEs, are freely available online [89, 90].

Because the counts of ELEs derived from an induced pluripotent stem cell line (iPS DF 6.9) are significantly lower than those of other contexts (NC: 547; LC: 387; MC: 360; HC: 182; Fig. 1b), we only used this cell line to create the omnibus ELEs and excluded it from all other analyses.

To benchmark our primary approach (Fig. 1a), we developed an alternative method (Additional file 2: Fig. S4a) to identify conserved ELEs in the human genome that (1) displayed H3K27ac signals in both humans and mice for the same context [25] and (2) reached the same level of human-mouse sequence conservation as our primary approach. We termed this alternative “H3K27ac-conserved ELEs.” For each context, we first mapped H3K27ac peaks from the mouse to human genome using liftOver with the default setting and minMatch being 0.9, and then we intersected the coordinate-converted mouse H3K27ac peaks with human H3K27ac and accessible chromatin peaks in the same context to create H3K27ac-conserved ELEs. We compared the two approaches in 14 contexts that each had profiles of human chromatin accessibility, human and mouse H3K27ac available (Additional file 1: Table S10).

### ENCODE cCRE classifications

To assess their biochemical relevance, we compared ELEs with the human cCREs (version 3) cataloged in phase 3 of the ENCODE Project [9]. The ENCODE cCREs were classified into the following five groups (Fig. 1e). Distal enhancer-like signature (dELS) elements are cCREs with high DNase and H3K27ac signals that are located more than 2 kb from the nearest TSS. Proximal enhancer-like signature (pELS) elements are cCREs with high DNase and H3K27ac but low H3K4me3 signals that are located within 2 kb of a TSS. Promoter-like signature (PLS) elements are cCREs with high DNase and H3K4me3 signals that are located within 200 bp of a TSS. Other high-DNase and high-H3K4me3 (DNH3) elements are cCREs with high DNase and H3K4me3 signals but low H3K27ac signals that do not reside within 200 bp of a TSS. CTCF-only elements are cCREs that possess high DNase and CTCF signals but low signals for H3K4me3 and H3K27ac.

### SNP annotation

We stored each set of ELEs (omnibus or context-specific; NC, LC, MC or HC) as a BED file, consisting of one line per genomic interval (henceforth “element”). For each set of elements, we created the corresponding binary SNP annotation as:

1 $$\begin{aligned} a(j) = \textbf{1}\left\{ \text {SNP }j\text { falls inside at least one element of the set}\right\} , \end{aligned}$$

where *j* belongs to 5,961,159 EUR or 5,469,053 EAS common SNPs. Because omnibus ELEs aggregate all context-specific ELEs (Additional file 2: Fig. S1a), we can show:

2 $$\begin{aligned} a(j;\text { Omni}) ={} & {} \textbf{1}\left\{ \text {SNP }j\text { falls inside at least one omnibus element}\right\} \nonumber \\ ={} & {} \textbf{1}\left\{ \text {SNP }j\text { falls inside at least one element in one context}\right\} \nonumber \\ ={} & {} \max_{k}~a(j;\text { Context }k). \end{aligned}$$

Equation (2) provides an alternative way to create the SNP annotation for omnibus ELEs directly from SNP annotations of context-specific ELEs, without identifying omnibus ELEs first and then applying Eq. (1).

To assess the correlation of two binary SNP annotations (Fig. 1h, i), we computed Cramér’s *V* using the function “cramerV” from R package rcompanion [91] (version 2.4.16). To assess the correlation between a binary and a quantitative annotation (Fig. 1i), we computed Pearson’s *R* using the R built-in function “cor.test” [92] (version 4.2.1).

### GWAS

We collected GWAS summary statistics from 312 UK Biobank, 108 EUR, and 48 EAS studies (Additional file 1: Table S5). The sample size of 468 datasets ranged from 14,267 to 1,320,016, with a median of 452,264. All datasets had observed-scale heritability $Z\text {-scores}\ge 6$ as estimated by S-LDSC (see below). All datasets were processed as previously described [13, 26].

### Heritability enrichment

To assess the heritability enrichment of a SNP annotation in a GWAS, we used S-LDSC [13] (version 1.0.1) with 1000 Genomes [34] phase 3 as the linkage disequilibrium (LD) reference panel (9,997,231 EUR and 8,768,561 EAS reference SNPs) and 96 annotations from the baselineLD model [26] (version 2.2) as covariates, which capture diverse genomic functions such as translation, regulation, and selection (Additional file 1: Table S4).

To analyze all (omnibus or context-specific) ELEs without considering human-mouse sequence conservation (NC) in a GWAS, we modeled the variance of effect size for SNP *j* as

3 $$\begin{aligned} \text {Var}(\beta _j)=\tau _0 +{\sum _{d=1}^{96}}\tau ^B_d \cdot a^B_d(j) + \tau ^N \cdot a^N(j), \end{aligned}$$

where $\tau _0$ is the background per-SNP contribution to heritability, $a^B_d(j)$ is the value of SNP *j* for one of the 96 baseline annotations, $a^N(j)=1$ if SNP *j* falls inside any ELE and 0 otherwise, and $\{\tau _d^B,\tau ^N\}$ are per-SNP contributions of one unit of the corresponding annotations to heritability. Equation (3) allows us to assess the contribution of ELEs to heritability conditional on 96 known annotations, which helps reduce bias due to model mis-specification [13, 26].

To analyze conserved (omnibus or context-specific) ELEs at each level of human-mouse sequence conservation (LC, MC, HC) in a GWAS, we extended Eq. (3) as

4 $$\begin{aligned} \text {Var}(\beta _j)=\tau _0 +{\sum _{d=1}^{96}}\tau ^B_d \cdot a^B_d(j) + \tau ^N \cdot a^N(j) + \tau ^C \cdot a^C(j), \end{aligned}$$

where $a^C(j)=1$ if SNP *j* falls inside any conserved ELE and 0 otherwise, and $\tau ^C$ is the per-SNP contribution of one unit of the conserved ELE annotation to heritability. Like Eq. (3), this model captures the unique contribution of conserved ELEs to heritability conditional on 96 known annotations and ELEs without sequence conservation in the same context.

We used two quantities [13, 26] to summarize the S-LDSC results. First, we computed the heritability enrichment of an annotation *a* in a GWAS as

5 $$\begin{aligned} \text {Enrichment}_a = \frac{h^2_a~/~h^2}{|a|~/~p},\quad h^2_a=\sum \limits _{j\in a}\text {Var}(\beta _j),\quad h^2=\sum \limits _{j=1}^p\text {Var}(\beta _j), \end{aligned}$$

where |*a*| is the number of common SNPs with annotation *a*, *p* is the total number of common SNPs ($p=5,961,159$ for EUR and 5,469,053 for EAS), and $h^2_a$ and $h^2$ are heritabilities due to |*a*| common SNPs with annotation *a* and *p* common SNPs respectively. Second, we computed the standardized effect size ($\tau ^\star$) of an annotation *a* in a GWAS as

6 $$\begin{aligned} \tau ^\star _a=\frac{p\cdot \text {SD}_a}{h^2}\cdot \tau _a, \end{aligned}$$

where $\text {SD}_a$ is the standard deviation of annotation *a* across *p* common SNPs and $\tau _a$ is the original effect size for annotation *a* in Eqs. (3) and (4). Both quantities can be compared across GWAS and annotations. Unlike the enrichment in Eq. (5), $\tau ^\star$ in Eq. (6) can capture the unique effect of annotation *a* conditional on all other annotations in Eqs. (3) and (4).

To meta-analyze the S-LDSC results across traits and contexts, we used the function “meta.summaries” from R package rmeta [93] (version 3.0) as previously described [13, 26]. For both heritability enrichment and $\tau ^\star$, we performed random-effects meta-analysis of individual estimates and SEs to obtain meta-analyzed estimates and SEs (Fig. 2a, b, Fig. 3a, b; Additional file 2: Figs. S2-S4). To find the *P*-value for meta-analyzed heritability enrichment, we first meta-analyzed $(h^2_a/|a|)-[(h^2-h^2_a)/(p-|a|)]$ and then computed a one-sided *Z*-score to test if this difference is greater than 0. To find the *P*-value for meta-analyzed $\tau ^\star$, we computed a one-sided *Z*-score to test if the meta-analyzed estimate is greater than 0.

To assess the concordance of S-LDSC results between EUR and EAS GWAS of the same trait, we use the function “deming” from R package deming [94] (version 1.4) as previously described [35]. For each annotation, we fitted a generalized Deming regression of EUR estimates on EAS estimates across 34 traits, while accounting for SEs.

### Fine mapping

The fine-mapping results [38] of 94 traits in UK Biobank (version 1.1) were produced by FINEMAP [95] and SUSIE [96]. Here, we excluded variants without any 95% credible set assigned, variants in LD ($R^2>0.6$) with a variant failing the Hardy-Weinberg equilibrium test ($P<1\times 10^{-12}$), and variants in LD ($R^2>0.8$) with a common EUR structural variant. We further intersected the variants with 9,997,231 EUR SNPs in 1000 Genomes [34], yielding a final set of 515,848 unique SNPs for this study.

### Trait-associated HC ELEs

We previously developed RSS-NET [39] that integrates GWAS summary statistics with genomic annotations to identify genetic enrichments and associations simultaneously. Here, we expanded this Bayesian framework to prioritize trait-associated HC ELEs (Additional file 2: Note S1). Specifically, we combined the RSS likelihood [97] with a new prior distribution as follows:

7 $$\begin{aligned} \beta _j \sim{} & {} \pi _j\cdot N\left(0,~\sigma _j^2\right) + (1-\pi _j)\cdot \delta _0,\end{aligned}$$

8 $$\begin{aligned} \pi _j ={} & {} \left( 1+10^{-(\theta _0 + a_j\cdot \theta )}\right) ^{-1},\end{aligned}$$

9 $$\begin{aligned} \sigma _j^2 ={} & {} \sigma _0^2 + a_j\cdot \sigma ^2, \end{aligned}$$

where $\beta _j$ denotes the effect of SNP *j* on a given trait, $\pi _j$ denotes the probability that SNP *j* is associated with the trait ($\beta _j\ne 0$), $N(0,~\sigma _j^2)$ denotes a normal distribution with mean 0 and variance $\sigma _j^2$ specifying the effect size of a trait-associated SNP *j*, $\delta _0$ denotes point mass at zero ($\beta _j=0$), and $a_j=1$ if SNP *j* falls inside HC ELEs and 0 otherwise. In Eq. (8), the baseline parameter $\theta _0<0$ captures the genome-wide background fraction of trait-associated SNPs, and the enrichment parameter $\theta >0$ reflects the increase in probability that a SNP inside HC ELEs is trait-associated [39, 82, 98]. In Eq. (9), the baseline parameter $\sigma _0^2$ captures the genome-wide background effect size of trait-associated SNPs, and the enrichment parameter $\sigma ^2$ reflects the increase in effect size of trait-associated SNPs inside HC ELEs [13, 39, 98]. We specified hyper-priors on the unknown parameters $\left\{\theta _0,\theta ,\sigma _0^2,\sigma ^2\right\}$ (Additional file 2: Note S3) and used variational inference to compute posterior distributions as previously described [39, 82]. The implementation of this RSS-NET extension is freely available online [99, 100].

To assess whether HC ELEs are enriched for genetic associations with a target trait (Figs. 5a and 6a), we computed a Bayes factor (BF):

10 $$\begin{aligned} \text {BF} = \frac{f(\textbf{D}\mid \theta>0\text { or }\sigma ^2>0)}{f(\textbf{D}\mid \theta =0\text { and }\sigma ^2=0)}, \end{aligned}$$

where $f(\cdot )$ denotes the marginal likelihood for the extended RSS-NET model and $\textbf{D}$ is a shorthand for the input data including GWAS summary statistics, LD estimates, and SNP annotations of HC ELEs ($a_j$). A larger BF indicates stronger evidence for enrichment of genetic associations.

To identify if a HC ELE is associated with a trait, we used $P_1$, the posterior probability that at least one SNP in the HC ELE is trait-associated:

11 $$\begin{aligned} P_1=1-\Pr (\beta _j=0 \text { for any SNP } j \text { inside this HC ELE}\mid \textbf{D}). \end{aligned}$$

A larger $P_1$ indicates stronger evidence for association between a HC ELE and a trait. For each HC ELE, $P_1^\text {H}$, $P_1^\text {N}$, and $P_1^\text {B}$ are $P_1$ values evaluated with different definitions of $a_j$ (Figs. 5b and 6b). For $P_1^\text {H}$, $a_j=1$ if SNP *j* falls inside HC ELEs and 0 otherwise, which corresponds to the enrichment model for HC ELEs. For $P_1^\text {N}$, $a_j=1$ if SNP *j* falls inside NC ELEs and 0 otherwise, which corresponds to the enrichment model for NC ELEs. For $P_1^\text {B}$, $a_j=0$ for any SNP *j*, which corresponds to the baseline model without any enrichment and is equivalent to setting $\theta =\sigma ^2=0$ in Eqs. (8) and (9).

We evaluated the RSS-NET extension through a large array of simulations (Additional file 2: Note S2). To reduce the computation, we performed simulations on 348,965 genome-wide common SNPs [101], with 19,335 SNPs annotated by HC omnibus ELEs ($a_j=1$). To mimic the genetic architectures of various complex traits, we specified 8 simulation scenarios with varying proportions of (1) trait-associated SNPs and (2) phenotypic variation explained by all SNPs. For each scenario, we simulated 200 “positive” datasets where SNP effects ($\beta _j$) were simulated from priors (8)-(9) with the presence of enrichment parameters ($\theta >0$ or $\sigma ^2>0$) as well as 200 “negative” datasets where SNP effects ($\beta _j$) were simulated from priors (8)-(9) without any enrichment ($\theta =0$ and $\sigma ^2=0$). To ensure a fair comparison in each scenario, we matched the positive and negative datasets by the proportions of (1) trait-associated SNPs and (2) phenotypic variation explained by all SNPs. We combined the simulated SNP effects with the genotypes of 348,965 genome-wide SNPs from 1458 individuals [101] to simulate phenotypes using an additive multiple-SNP model with Gaussian noise as previously described [39]. We performed the standard single-SNP analysis of simulated individual-level datasets to generate GWAS summary statistics, on which we compared RSS-NET results with the ground truth of each simulation scenario. The simulation results show that the RSS-NET extension produces accurate posterior estimation for model parameters (Additional file 2: Fig. S5), as well as valid inference of both enrichments (Additional file 2: Fig. S6) and associations (Additional file 2: Figs. S7-S8).

We applied the RSS-NET extension to the GWAS meta-analysis summary statistics of BMI [40] and schizophrenia [41] as previously described [39, 82, 97]. Since both GWAS datasets were derived from cohorts of EUR ancestry, we supplied the RSS-NET extension with reference LD estimates based on the haplotypes of unrelated individuals with EUR ancestry from Phase 3 of the 1000 Genomes Project [34]. Prior to the analysis of each GWAS with the RSS-NET extension, we executed a series of quality control procedures to ensure consistency between the GWAS summary statistics and the reference LD estimates (Additional file 2: Note S4 and Fig. S16). For each GWAS, we analyzed both NC and HC omnibus ELEs using the same hyper-priors (Additional file 2: Note S3). We did not analyze HC context-specific ELEs with RSS-NET because they contain less than 0.45% of common SNPs (Fig. 1g), leading to sparse SNP annotations (i.e., $a_j=0$ for most SNPs). Reliable estimation of the RSS-NET enrichment parameters $(\theta ,\sigma ^2)$ in priors (8)-(9) requires sufficient SNPs with $a_j=1$.

### Motif enrichment

We used the HOMER [102] command “findMotifsGenome.pl” (version 4.11) to identify genomic regions specifically enriched in a target set of sequences against a background set. We used the exact regions provided (“-size given”) and searched for known motifs (“-mknown”) in the curated list of 1465 unique motifs [83, 103]. Beyond the 1465 known motifs, we also identified de novo motifs and matched them to known motifs based on similarity of motif matrices (Figs. 5c–d and 6c–d). For each motif, we computed a fold change of fractions of target against background sequences containing the motif and a binomial *P*-value to quantify enrichment. We identified a significant enrichment of a motif when the fold change $\ge 2$ and $P\le 1\times 10^{-12}$.

### Target genes of HC ELEs

To link trait-associated HC ELEs to putative target genes, we used cCRE-gene linkages derived from recent studies of the adult mouse cerebrum [23], fetal [104], and adult [105] human brain. We used BEDTools to overlap HC ELEs with cCREs in these datasets and then used the cCRE-gene linkages to identify putative target genes for HC ELEs.

The adult mouse study [23] integrated snATAC-seq with scRNA-seq data to identify gene-cCRE connections on the basis of both co-accessibility between cCREs and positive association with gene expression, resulting in 813,638 linkages that connect 261,204 cCREs to 12,722 putative target genes for 160 brain cell types. For each cell type, the BEDPE file of gene-cCRE connections is freely available online [106]. When using these linkages, we first converted HC ELEs (GRCh37) to regions in the mouse genome (GRCm38), overlapped them with the mouse cCREs to find the linked mouse genes, and then converted the mouse gene symbols (MGI) to human gene symbols (HGNC).

The fetal human study [104] used Hi-C data to produce 63,653 linkages of 33,862 enhancers and 10,892 genes for the cortical plate as well as 63,740 linkages of 34,044 enhancers and 11,146 genes for the germinal zone. These linkages are freely available online [107]; see also “Table S5: enhancer-gene predictions” of the original publication [104].

The adult human study [105] first combined Hi-C linkages, quantitative trait loci, and transcription factor (TF) binding sites to build a reference network of gene regulation in brain and then refined enhancer-gene connections by relating the activity of TFs to expression of target genes via elastic net regression. The network construction used both the repeat-masked TF binding site map (GRN1) and the complete map (GRN2). GRN1 has 577,529 linkages of 71,097 enhancers and 13,308 genes. GRN2 has 531,322 linkages of 70,532 enhancers and 13,330 genes. These linkages are freely available online [108] as INT-11 (GRN1) and INT-14 (GRN2).

### External databases

To interpret our findings, we used the following external databases: UCSC Genome Browser [87] for phastCons 100-vertebrate scores [18], Metascape [109] for biological pathways (version 3.5), MGI [110] for knockout mouse genes (version 6.18), OMIM [111] for human Mendelian genes, TTD [112] for drug target genes (version 8.1.01), and GWAS Catalog [74] for GWAS-implicated genes (version 1.0.2).

### Supplementary information

**Additional file 1:** Supplementary Tables 1-26.

**Additional file 2:** Supplementary information. This file includes Supplementary Notes 1-4, legends for Supplementary Tables 1-26, and Supplementary Figures 1-16.

**Additional file 3.** Review history.

## Data Availability

The BED files of ELEs (omnibus or context-specific, all or conserved) and the source code are freely available at https://doi.org/10.5281/zenodo.8317239 [89] and https://github.com/SUwonglab/m2h-ele [90]. The source code and documentation of RSS-NET are freely available at https://doi.org/10.5281/zenodo.4553387 [99] and https://github.com/SUwonglab/rss-net [100]. Links and identifiers of all other data and codes are specified in Methods, References, and Additional file 1: Tables S1, S5, S10.
