# Supplementary material for: Genetic effects of sequence-conserved enhancer-like elements on human complex traits
Source: Genome Biol. 2024 Jan 2;25:1. doi: 10.1186/s13059-023-03142-1 (PMC10759394; doi:10.1186/s13059-023-03142-1)
Supplement: Supplementary file 2 — Additional file 2: Supplementary information. This file includes Supplementary Notes 1-4, legends for Supplementary Tables 1-26, and Supplementary Figures 1-16. [file 13059_2023_3142_MOESM2_ESM.pdf]

Supplementary information for "*Genetic effects of sequence-conserved enhancer-like elements on human complex traits*"

Xiang Zhu, Ph.D., xiangzhu@psu.edu
The Pennsylvania State University

Supplementary Notes

Note S1: Connection between the original RSS-NET and the extension

The RSS-NET extension in the present study is closely related to the original RSS-NET model. Both models are built on the same multiple-SNP regression likelihood for GWAS summary statistics. Furthermore, the prior distribution used in the RSS-NET extension is a specific instance of the prior used in the original RSS-NET model, as demonstrated below.

In the original RSS-NET model, the prior distribution is given by

$$\begin{aligned} \beta_j &\sim \pi_j \cdot \mathcal{N}(\mu_j,\ \sigma_0^2) + (1-\pi_j)\cdot \delta_0, \\ \pi_j &= \left(1 + 10^{-(\theta_0 + \ell_j \cdot \theta)}\right)^{-1}, \\ \mu_j &= \textstyle\sum_{g \in \mathbf{O}_j} w_{jg} \cdot \gamma_{jg}, \quad \gamma_{jg} \sim \mathcal{N}(0,\ \sigma^2), \end{aligned}$$

where β_j is the true effect of each SNP j, π_j denotes the probability that SNP j is associated with the trait ($\beta_j \neq 0$), $\mathcal{N}(\mu_j,\ \sigma_0^2)$ denotes a normal distribution with mean μ_j and variance σ_0^2 specifying the effect size of a trait-associated SNP j, δ_0 denotes the point mass at zero ($\beta_j = 0$), $\ell_j = 1$ if SNP j is within 100 kb of a given regulatory network and 0 otherwise, $\mathbf{O}_j$ is the set of all nearby or distal genes contributing to the total effect of SNP j, w_{jg} measures the relevance between SNP j and gene g, γ_{jg} denotes the random effect of SNP j on a trait due to gene g, and $\{\theta_0, \theta, \sigma_0, \sigma\}$ are hyper-parameters capturing baseline and enrichment effects.

After integrating out the random effects $\{\gamma_{jg}\}$, we can rewrite the prior above as follows:

$$\begin{aligned} \beta_j &\sim \pi_j \cdot \mathcal{N}(0,\ \sigma_j^2) + (1-\pi_j)\cdot \delta_0, \\ \pi_j &= \left(1 + 10^{-(\theta_0 + \ell_j \cdot \theta)}\right)^{-1}, \\ \sigma_j^2 &= \sigma_0^2 + \left(\textstyle\sum_{g \in \mathbf{O}_j} w_{jg}^2\right) \cdot \sigma^2. \end{aligned}$$

Lastly, we set $\ell_j = a_j$ and $\sum_{g \in \mathbf{O}_j} w_{jg}^2 = a_j$, resulting in the prior used in the RSS-NET extension:

$$\begin{aligned} \beta_j &\sim \pi_j \cdot \mathcal{N}(0,\ \sigma_j^2) + (1-\pi_j)\cdot \delta_0, \\ \pi_j &= \left(1 + 10^{-(\theta_0 + a_j \cdot \theta)}\right)^{-1}, \\ \sigma_j^2 &= \sigma_0^2 + a_j \cdot \sigma^2, \end{aligned}$$

where $a_j = 1$ if SNP j falls inside HC ELEs and 0 otherwise. Due to this close connection between the original RSS-NET and the present extension, we can reuse the efficient algorithm developed for the original RSS-NET to fit the extension model.

Note S2: Details of the simulations to assess the RSS-NET extension

We used real genotypes of 348965 genome-wide SNPs on chromosomes 1-22 from 1458 individuals in the UK Blood Service Control Group to simulate phenotype data, and then computed the standard single-SNP association summary statistics. On these summary data, we compare the results of RSS-NET extension with the ground truth of each simulation scenario.

We used the HC omnibus ELEs to create binary annotations for 348965 genome-wide SNPs. Specifically, we let $a_j = 1$ if SNP j falls inside the HC omnibus ELEs, and let $a_j = 0$ otherwise. There are 19335 SNPs mapped to the HC omnibus ELEs with annotation $a_j = 1$.

To ensure comparable proportions of true genetic signals across simulated datasets in a given scenario, we simulated causal indicators for negative and positive datasets in a paired way. We first simulated the causal indicator ζ_j of each SNP j in a positive dataset as:

$$\zeta_j \sim \text{Bernoulli}(\pi_j) \quad \text{and} \quad \pi_j = \left(1 + 10^{-(\theta_0 + a_j \cdot \theta)}\right)^{-1},$$

where θ_0 and θ are the baseline and enrichment proportion parameters, respectively. We then counted the number of causal SNPs in this positive dataset as $n_c := \sum_j \zeta_j$, and randomly chose n_c SNPs as causal SNPs for the corresponding negative dataset.

For positive datasets, we simulated the true effect β_j of each SNP j as

$$\beta_j \mid \zeta_j = 0 \sim \delta_0 \quad \text{and} \quad \beta_j \mid \zeta_j = 1 \sim \mathcal{N}(0,\, \sigma_0^2 + a_j \cdot \sigma^2),$$

where δ_0 denotes the point mass at zero, σ_0^2 and σ^2 are the baseline and enrichment magnitude parameters respectively, and a_j denotes the annotation of SNP j based on HC omnibus ELEs. For negative datasets, we simulated β_j from the same model above with $\sigma^2 = 0$.

To ensure comparable total genetic signals between negative and positive datasets, we simulated phenotypes by matching signal-to-noise ratios across simulated datasets. Specifically, we simulated phenotype y_i of individual i as $y_i = \sum_{j=1}^{p} x_{ij} \cdot \beta_j + \epsilon_i$, $\epsilon_i \sim \mathcal{N}(0, \tau^{-1})$, where $p = 348965$ and x_{ij} is the genotype of SNP j for individual i. Let $\mathbf{y} = [y_i]$ and $\mathbf{X} = [x_{ij}]$. The true value of residual variance τ^{-1} is determined by the total proportion of variance in phenotype $\mathbf{y}$ explained by all available SNPs in $\mathbf{X}$: PVE $= V(\mathbf{X}\boldsymbol{\beta}) \,/\, [\tau^{-1} + V(\mathbf{X}\boldsymbol{\beta})]$, where $V(\mathbf{X}\boldsymbol{\beta})$ is the sample variance of $\mathbf{X}\boldsymbol{\beta}$.

We set the true values of hyper-parameters for positive datasets as follows: baseline proportion parameter $\theta_0 \in \{-2, -4\}$, enrichment proportion parameter $\theta = 2$, baseline magnitude parameter $\sigma_0 = 1$, enrichment magnitude parameter $\sigma \in \{0, 2\}$, and PVE $\in \{0.2, 0.6\}$, resulting in a total of 8 unique simulation scenarios. For each scenario of $\{\theta_0, \theta, \sigma_0, \sigma, \text{PVE}\}$, we simulated 200 negative and 200 positive datasets independently.

We applied the RSS-NET extension to simulated datasets with the following hyper-parameter grids. The baseline parameters θ_0 and σ_0 were fixed as their true values. The grid on the enrichment proportion parameter θ was $\{0, 1.5, 1.75, 2, 2.25, 2.5\}$. The grid on the enrichment magnitude parameter σ was $\{0, 0.25, 0.5, 0.75, 1\}$ if the true value of σ was zero; otherwise it was $\{0, 1.5, 1.75, 2, 2.25, 2.5\}$. For the same simulation scenario, we used the same hyper-parameter grid in the analysis of all negative and positive datasets. Posterior estimates of $\{\theta, \sigma\}$ are shown in Fig. S5.

We evaluated the performance of the RSS-NET extension for enrichment testing on the paired positive and negative datasets in each simulation scenario. For all datasets the target of enrichment testing is the full set of HC omnibus ELEs. A false positive occurs if a method identifies enrichment

in a negative dataset. A true positive occurs if a method identifies enrichment in a positive dataset. We evaluated the performance of enrichment testing by plotting the receiver operating characteristic (ROC) curve and computing the area under the ROC curve (AUROC) for each simulation scenario. Both metrics are implemented in the R package plotROC. The results are shown in Fig. S6.

We evaluated the performance of the RSS-NET extension for association testing only on positive datasets in each simulation scenario. For each positive dataset, a HC ELE is defined as "trait-associated" if at lease one SNP j within this element has non-zero effect ($\beta_j \neq 0$). In each simulated dataset, we quantified the strength of genetic association for each HC ELE by P_1^{H}, the posterior trait-associated probability evaluated under the model that HC ELEs are enriched for genetic associations with the trait. An association between a non-trait-associated HC ELE and the trait is a "false positive". An association between a trait-associated HC ELE and the trait is a "true positive".

We visualized the performance of the RSS-NET extension for association testing in two distinct ways. First, we plotted the ROC curve and computed the AUROC for each simulation scenario, as we did in the assessment of enrichment testing above. Unlike the balanced structure of enrichment testing (200 positive and 200 negative datasets in each scenario), the numbers of trait-associated HC ELEs (true labels) and non-trait-associated HC ELEs (false labels) differed a lot for most simulated datasets. Due to this imbalanced structure, we additionally plotted the precision-recall (PRC) curve and computed the area under the PRC curve (AUPRC) for each simulation scenario. Both metrics are implemented in the R package precrec. The results are shown in Fig.s S7 and S8.

Note S3: Hyper-parameter inference for the RSS-NET extension

The RSS-NET extension consists of four unknown hyper-parameters $\{\theta_0, \theta, \sigma_0^2, \sigma^2\}$. We followed the approach from the original RSS-NET publication to infer these unknown hyper-parameters. Specifically, we first introduced two free parameters $\{\eta, \rho\}$ to re-parameterize $\{\sigma_0^2, \sigma^2\}$:

$$\sigma_0^2 = \eta \cdot (1-\rho) \cdot \left(\sum_{j=1}^{p} \frac{\pi_j}{n \hat{s}_j^2} \right)^{-1}, \quad \sigma^2 = \eta \cdot \rho \cdot \left(\sum_{j=1}^{p} \frac{\pi_j \cdot a_j}{n \hat{s}_j^2} \right)^{-1},$$

where η represents the proportion of the total phenotypic variation explained by p SNPs, and ρ represents the proportion of total genetic variation explained by SNPs with a given annotation ($a_j = 1$). We then placed independent uniform grid hyper-priors on $\{\theta_0, \theta, \eta, \rho\}$.

When applying the RSS-NET extension to the GWAS of BMI and schizophrenia, we used the following grid hyper-priors: $\theta_0 = -2$, $\theta \in \{0, 0.25, 0.5, 0.75, 1\}$, $\eta \in \{0.2, 0.3\}$ and $\rho \in \{0, 0.2, 0.4, 0.6, 0.8\}$. In each GWAS, we applied the RSS-NET extension under three models: (1) 'Baseline model', where $a_j = 0$ for any SNP j across the genome; (2) 'Enrichment model for NC ELEs', where $a_j = 1$ for SNP j inside NC ELEs and 0 otherwise; (3) 'Enrichment model for HC ELEs', where $a_j = 1$ for SNP j inside HC ELEs and 0 otherwise. The following table shows the posterior means of $\{\theta_0, \theta, \sigma_0^2, \sigma^2\}$ estimated in each GWAS under each model.

GWAS	BMI			Schizophrenia		
Model	Baseline	NC ELE	HC ELE	Baseline	NC ELE	HC ELE
$\mathrm{E}(\theta_0 \mid \mathbf{D})$	-1.9996	-2.0213	-2.0140	-1.9884	-1.9911	-1.9903
$\mathrm{E}(\theta \mid \mathbf{D})$	NA	0.4536	0.9323	NA	0.7410	0.7486
$\mathrm{E}(\sigma_0^2 \mid \mathbf{D})$	1.2591×10^{-5}	1.0287×10^{-5}	1.0946×10^{-5}	1.6039×10^{-4}	8.0174×10^{-5}	1.1863×10^{-4}
$\mathrm{E}(\sigma^2 \mid \mathbf{D})$	NA	1.3842×10^{-7}	1.1122×10^{-6}	NA	1.1712×10^{-15}	6.8689×10^{-5}

The posterior estimates of hyper-parameters $\{\theta_0, \theta, \sigma_0^2, \sigma^2\}$ help explain the larger number of genetic associations for BMI and schizophrenia identified under the enrichment model for HC ELEs, compared to the baseline model. On the same GWAS, the RSS-NET extension gives similar estimates for the baseline parameters $\{\theta_0, \sigma_0^2\}$ under both the baseline and enrichment models, and it produces positive estimates for the enrichment parameters $\{\theta, \sigma^2\}$ if HC ELEs are identified as enriched for genetic associations with BMI (Fig. 5a) and schizophrenia (Fig. 6a). The positive estimate of θ increases $\pi_j = \left(1 + 10^{-(\theta_0 + a_j \cdot \theta)}\right)^{-1}$, the prior probability of association for SNPs in HC ELEs, which in turn increases the posterior probability of association for these SNPs. The positive estimate of σ^2 increases $\sigma_j^2 = \sigma_0^2 + a_j \cdot \sigma^2$, the prior effect size for trait-associated SNPs in HC ELEs, which in turn increases the posterior effect size for these SNPs. Collectively, the positive enrichment parameter estimates enhance the evidence for associations between SNPs in HC ELEs and the GWAS trait, leading to the identification of more trait-associated elements under the enrichment model for HC ELEs (Fig.s 5b-d, 6b-d, S9; Table S15).

Note S4: Quality control for reference LD estimates in the RSS-NET extension

Built upon the multiple-SNP regression likelihood for GWAS summary statistics, the RSS-NET extension requires the input of reference LD estimates that align with GWAS summary statistics. In the present study, we have executed a series of quality control (QC) procedures to ensure consistency between GWAS summary statistics and reference LD estimates when running the RSS-NET extension. The following table lists the major sources of GWAS-LD inconsistencies to date, as well as the specific QC procedures that we have implemented in this study to address these issues.

**Source of potential GWAS-LD mismatches**	**QC procedure used in the present study**
Differences in population or ancestry between the sample to generate GWAS summary statistics and the sample to derive reference LD estimates.	Since the GWAS summary statistics of BMI and schizophrenia analyzed in this study were generated from European ancestry cohorts, we estimated LD using the haplotypes of unrelated individuals with European ancestry from Phase 3 of the 1000 Genomes Project.
Errors in genotyping or imputation, as well as genetic variants with incorrectly specified effect alleles.	We used the HapMap3 SNPs as an approximation of the well-imputed genome-wide SNPs, and we further excluded SNPs on sex chromosomes, SNPs with minor allele frequency less than 1%, and SNPs in the major histocompatibility complex region to avoid potential LD mismatches caused by poorly genotyped or imputed SNPs and incorrect allele encodings.
Inconsistent sample size coverage at different genetic variants when meta-analyzed cohorts are genotyped or imputed at variable density.	We excluded SNPs that were not analyzed in all individual cohorts in the GWAS meta-analysis, such as SNPs measured on custom arrays (e.g., Metabochip and Immunochip).
'Allele flip' in the sense that alleles in the GWAS summary statistics are encoded differently from those in the reference LD estimates.	For each SNP analyzed, we checked whether its alleles were consistently encoded in both the GWAS summary statistics and the reference LD estimates. We manually adjusted SNPs that used different allele encodings prior to any downstream analysis.

Supplementary Table Legends

Table S1: Human epigenomic data. Accession numbers of H3K27ac and chromatin accessibility profiles for 106 human tissues and cell types.

Table S2: Motif enrichments of ELEs. For each set of ELEs (omnibus or context-specific, all or conserved), significant motif enrichments are identified by HOMER ($\geq$ 2-fold and $P \leq 1 \times 10^{-12}$). For all ELEs without considering sequence conservation (NC), the background is set as randomly selected sequences that match the GC-content distribution of NC ELEs. For conserved ELEs (LC, MC, HC), the background is set as either (1) GC-matched random sequences or (2) NC ELEs in the same context.

Table S3: Pairwise correlations for ELE annotations of SNPs. For each set of ELEs (omnibus or context-specific, all or conserved), the binary SNP annotation is created for both EUR and EAS populations. For each population, two sets of genome-wide SNPs are used: 'all SNPs' (EUR: 9997231; EAS: 8768561); 'common SNPs' (EUR: 5961159; EAS: 5469053). For each pair of SNP annotations, the correlation is measured by Cramér's V, ranging from 0 (no correlation) to 1 (complete correlation).

Table S4: Correlations between ELE annotations and 96 known annotations of SNPs. The S-LDSC baselineLD model consists of 83 binary and 13 quantitative annotations of SNPs. The correlation between an ELE annotation and a binary S-LDSC annotation is measured by Cramér's V. The correlation between an ELE annotation and a quantitative S-LDSC annotation is measured by Pearson's R. The rest is the same as Table S3.

Table S5: GWAS data. Trait descriptions, PubMed IDs and sample sizes for 468 GWAS datasets.

Table S6: Heritability enrichments of omnibus ELEs. S-LDSC results of SNP annotations based on all omnibus ELEs (NC) and subsets with varying levels of sequence conservation (low: LC; moderate: MC; high: HC) for 468 GWAS datasets.

Table S7: Meta-analyzed heritability enrichments of omnibus ELEs. S-LDSC results of SNP annotations based on all omnibus ELEs (NC) and subsets with varying levels of sequence conservation (low: LC; moderate: MC; high: HC) meta-analyzed in each of the 30 strata.

Table S8: Heritability enrichments of HC context-specific ELEs. S-LDSC results of HC context-specific ELEs for 468 GWAS and 105 contexts.

Table S9: Meta-analyzed heritability enrichments of HC context-specific ELEs. S-LDSC results of HC context-specific ELEs meta-analyzed in each of the 432 strata.

Table S10: Mouse epigenomic data. Accession numbers of H3K27ac profiles for 14 mouse contexts and human epigenomic data matched to each context.

Table S11: Heritability enrichments of HC ELEs with H3K27ac conservation. S-LDSC results of SNP annotations based on NC context-specific ELEs, HC context-specific ELEs using human-mouse H3K27ac comparison, and HC context-specific ELEs using human-mouse genome comparison, for 379 GWAS and 14 contexts.

Table S12: Meta-analyzed heritability enrichments of ELEs with H3K27ac conservation. S-LDSC results of HC context-specific ELEs using human-mouse H3K27ac comparison and human-mouse genome comparison, meta-analyzed in each of the 34 strata.

Table S13: Fractions of fine-mapped SNPs in omnibus ELEs. Each row reports the following:

$$\frac{\#\{\text{fine-mapped SNPs that fall inside a region and have PIPs} \geq \text{a threshold}\}}{\#\{\text{fine-mapped SNPs that fall inside a region}\}},$$

for a combination of GWAS dataset, fine-mapping method and PIP threshold (columns 1-3), where the 'region' denotes the whole genome (column 4), all omnibus ELEs (NC; column 5), or omnibus ELEs with varying levels of sequence conservation (low: LC; moderate: MC; high: HC; columns 6-8).

Table S14: Fractions of fine-mapped SNPs in HC context-specific ELEs. Each row reports the following fraction (column 6):

$$\frac{\#\{\text{fine-mapped SNPs that fall inside HC ELEs and have PIPs} \geq 0.1\}}{\#\{\text{fine-mapped SNPs that fall inside HC ELEs}\}},$$

for a combination of GWAS dataset, fine-mapping method and context group (columns 1-3), together with a binomial P-value (column 7) testing if this fraction is greater than the fraction of all fine-mapped SNPs that have PIPs ≥ 0.1 (using the same GWAS data and fine-mapping method).

Table S15: Genetic associations of HC omnibus ELEs with BMI and schizophrenia. Each row represents a HC omnibus ELE (columns 1-3), and reports the posterior trait-associated probabilities estimated under the baseline model without any enrichment (P_1^{B}), under the enrichment model for NC ELEs (P_1^{N}), and under the enrichment model for HC ELEs (P_1^{H}), based on the GWAS of BMI (columns 5-7) and schizophrenia (columns 8-10). 'Neural' (column 4) indicates if the element contains any HC context-specific ELE derived from neural samples (1: yes; 0: no), as visualized in Fig.s S1f-i.

Table S16: Motif enrichments of HC ELEs associated with BMI. Each row represents either a de novo or known motif whose enrichment result is generated by HOMER. The target sequences are HC omnibus ELEs that are present in neural samples and are associated with BMI at $P_1^{\mathrm{H}} \geq 0.9$, as indicated in columns 4 and 7 of Table S15. The background sequences are HC omnibus ELEs that are present in neural samples and are associated with BMI at $P_1^{\mathrm{H}} \leq 0.1$.

Table S17: Motif enrichments of HC ELEs associated with schizophrenia. The target and background sequences are HC omnibus ELEs that are present in neural samples and are associated with schizophrenia at $P_1^{\mathrm{H}} \geq 0.9$ and $P_1^{\mathrm{H}} \leq 0.1$, respectively; see columns 4 and 10 of Table S15. The rest are the same as in Table S16.

Table S18: Putative target genes of BMI-associated HC ELEs with MEIS1-binding motif. Each row reports a published linkage between a likely target gene and a neural-related, BMI-associated HC omnibus ELE ($P_1^{\mathrm{H}} \geq 0.9$) that contains the enriched sequence motif recognized by MEIS1, together with the corresponding publication (PubMed ID).

Table S19: Pathway enrichments of target genes in Table S18. Each row reports a Metascape pathway (columns 1-3), the number and fraction of target genes that belong to this pathway (columns 4-5), the log10 P-value (column 6) based on the hypergeometric distribution (with all genes in the genome as the enrichment background), and the log10 q-value (column 7) based on the Benjamini-Hochberg procedure to account for multiple testing.

Table S20: Putative target genes of BMI-associated HC ELEs with DLX3-binding motif. All elements listed here contain instances of the enriched DLX3-binding motif. The rest are the same as in Table S18.

Table S21: Pathway enrichments of target genes in Table S20. The legend is the same as the legend of Table S19.

Table S22: External database lookup of genes in Tables S18 and S20. Each row reports the identifiers of a gene in Mouse Genome Informatics (MGI), Online Mendelian Inheritance in Man (OMIM) and Therapeutic Target Database (TTD). A blank entry indicates the absence of a gene in a database.

Table S23: Putative target genes of schizophrenia-associated HC ELEs with POU3F3-binding motif. The elements listed here are neural-related, schizophrenia-associated HC omnibus ELEs ($P_1^{\mathrm{H}} \geq 0.9$) that contain the enriched motif recognized by POU3F3. The rest are the same as in Table S18.

Table S24: Putative target genes of schizophrenia-associated HC ELEs with HAND1::TCF3-binding motif. The elements listed here are neural-related, schizophrenia-associated HC omnibus ELEs ($P_1^{\mathrm{H}} \geq 0.9$) that contain the enriched HAND1::TCF3 motif. The rest are the same as in Table S18.

Table S25: External database lookup of genes in Tables S23 and S24. The legend is the same as Table S22.

Table S26: GWAS Catalog lookup. The column heading description is available in the GWAS Catalog (`https://www.ebi.ac.uk/gwas/docs/fileheaders`).

Supplementary Figures

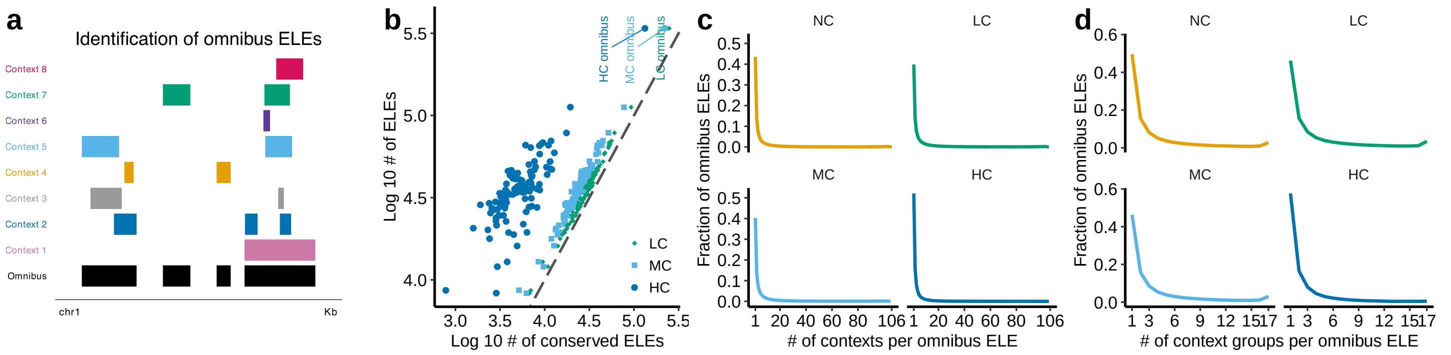

e

Omnibus ELEs	TSS-overlapping	TSS-proximal	TSS-distal
NC	0.86%	3.60%	95.54%
LC	1.12%	4.49%	94.39%
MC	1.53%	5.21%	93.26%
HC	3.14%	9.63%	87.23%

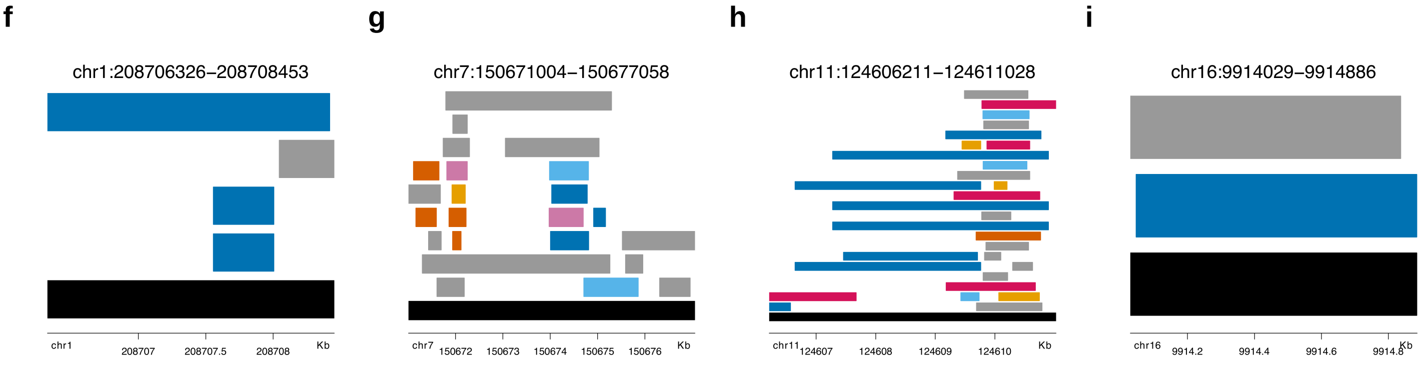

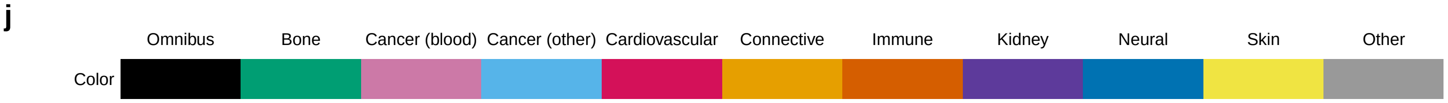

Figure S1: Identification and characterization of omnibus ELEs. a Context-specific ELEs from various contexts (colored bars) are first aggregated and then the overlapping segments are merged into 4 non-overlapping omnibus ELEs (black bars). **b** Numbers of context-specific and omnibus ELEs without considering sequence conservation (y-axis) and those with sequence conservation (x-axis). **c** Distributions of context counts for all omnibus ELEs (NC) and the sequence-conserved subsets (LC, MC, HC). **d** Distributions of context group counts for each NC, LC, MC and HC omnibus ELE. **e** Percentages of omnibus ELEs overlapping with, proximal to, and distal from annotated transcriptional start sites (TSSs). We first determine the center of each ELE, and then we categorize an ELE as (1) "TSS-overlapping" if its center is located within 200 bp of a TSS, (2) "TSS-proximal" if its center falls between 200 bp and 2 kb of a TSS, and (3) "TSS-distal" if the genomic distance from its center to the nearest TSS exceeds 2 kb. The annotated TSS list is obtained from UCSC RefGene hg19. **f-i** Illustration of 4 neural-related HC omnibus ELEs that contain significant genetic associations with BMI (**f-g**; Fig.s 5c-d and S11-12) and schizophrenia (**h-i**; Fig.s 6c-d and S13-14). **j** Color legends for context groups shown in **f-i**, as well as Fig.s 3 and S3.

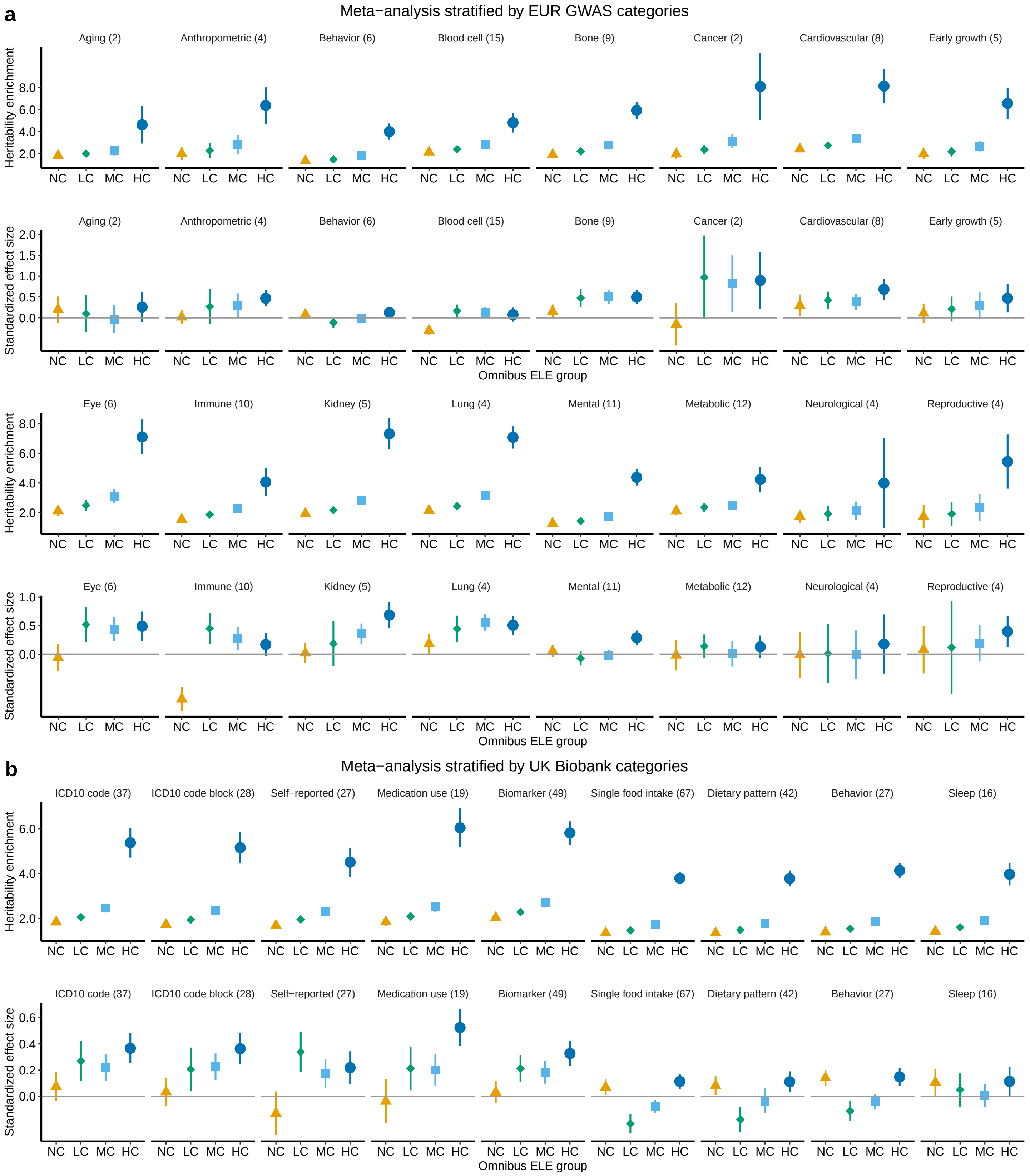

Figure S2: Additional results of heritability enrichments for sequence-conserved omnibus ELEs. Estimates meta-analyzed within each of **a** 16 trait groups for the 108 EUR GWAS datasets and **b** 9 trait groups for the 312 UK Biobank datasets. Numerical results are available in Table S7. The legend is the same as Fig.s 2a-b.

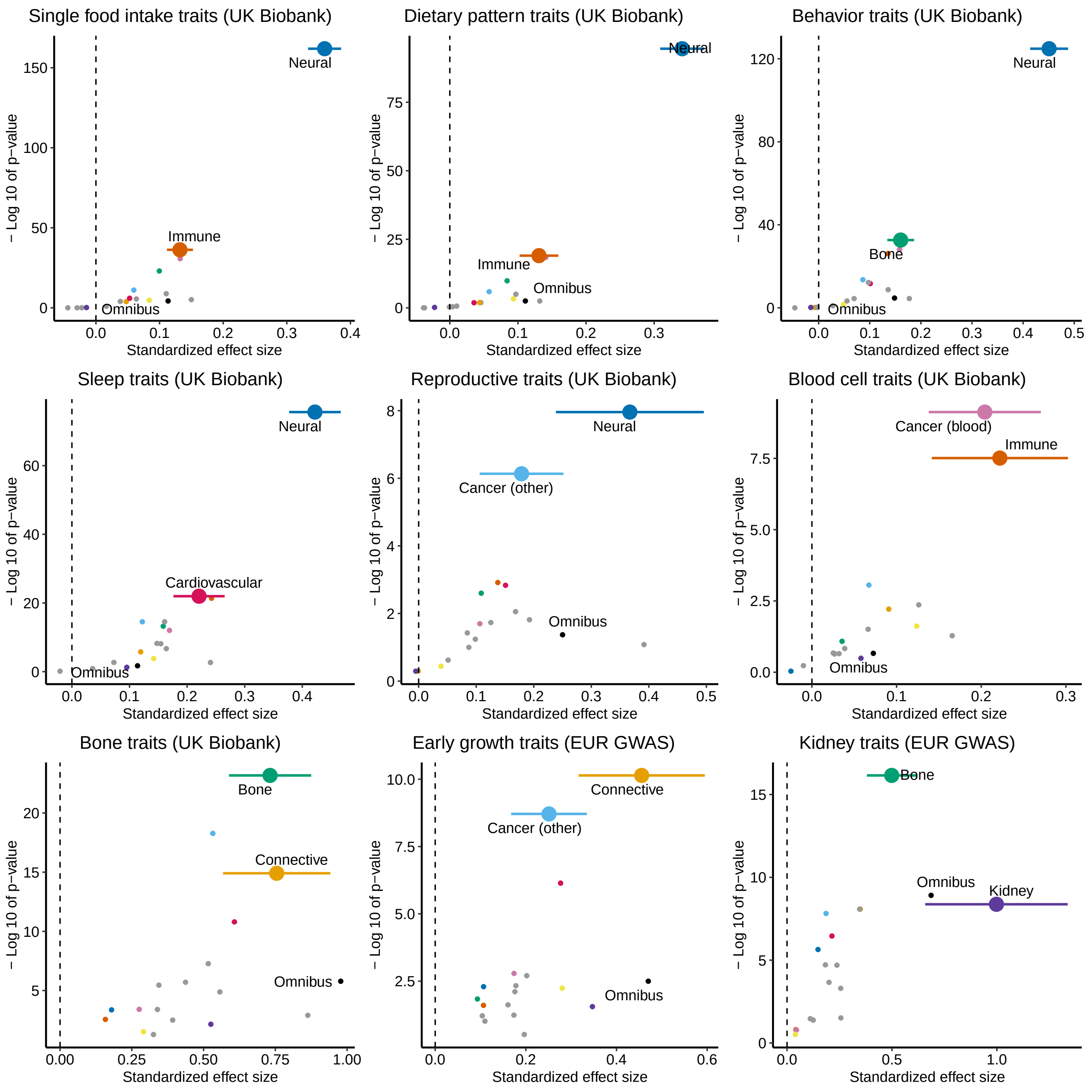

Figure S3: Additional results of heritability enrichments for HC context-specific ELEs. The legend is the same as Fig. 3a. For behavior, blood cell and bone traits, the results shown in Fig. 3a are based on the EUR GWAS datasets whereas the results shown here are based on the UK Biobank datasets.

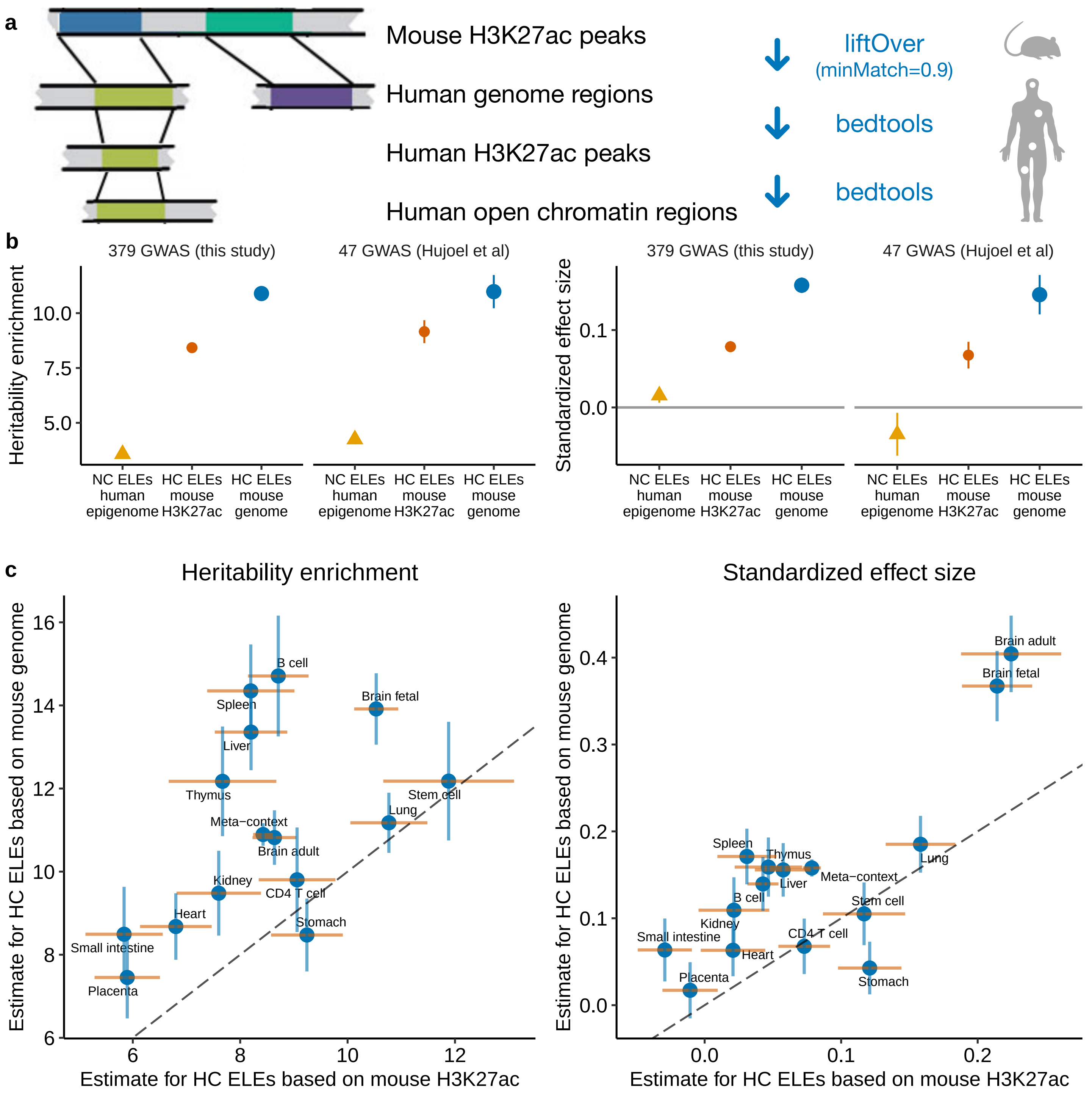

Figure S4: Comparison of common SNP heritability enrichments for HC ELEs based on the mouse genome and mouse H3K27ac profile. For each trait and each of the 14 contexts with mouse H3K27ac ChIP-seq data available (Table S10), we estimate the heritability enrichment and standardized effect size ($\tau^\star$) for HC ELEs based on the conservation with the mouse genome (Fig. 1a) and HC ELEs based on the conservation with the context-matched mouse H3K27ac profile, both conditioning on NC ELEs in the same context and 96 known annotations. **a** Schematic of creating HC ELEs based on the mouse H3K27ac profile. To achieve a fair comparison, HC ELEs based on the mouse genome and mouse H3K27ac profile use the same level of sequence conservation (minMatch=0.9). **b** Estimates meta-analyzed across 14 contexts and 379 GWAS datasets curated for this study, or a previously defined set of 47 independent GWAS datasets. **c** Estimates meta-analyzed across 379 GWAS datasets for each of the 14 contexts. The dashed lines have intercept 0 and slope 1. Numerical results of **b** and **c** are available in Table S12. The 'Meta-context' results shown in **c** correspond to the meta-analyzed results of 379 GWAS in **b**. For **b**-**c**, each point denotes an estimate and each error bar denotes ±1.96 SE.

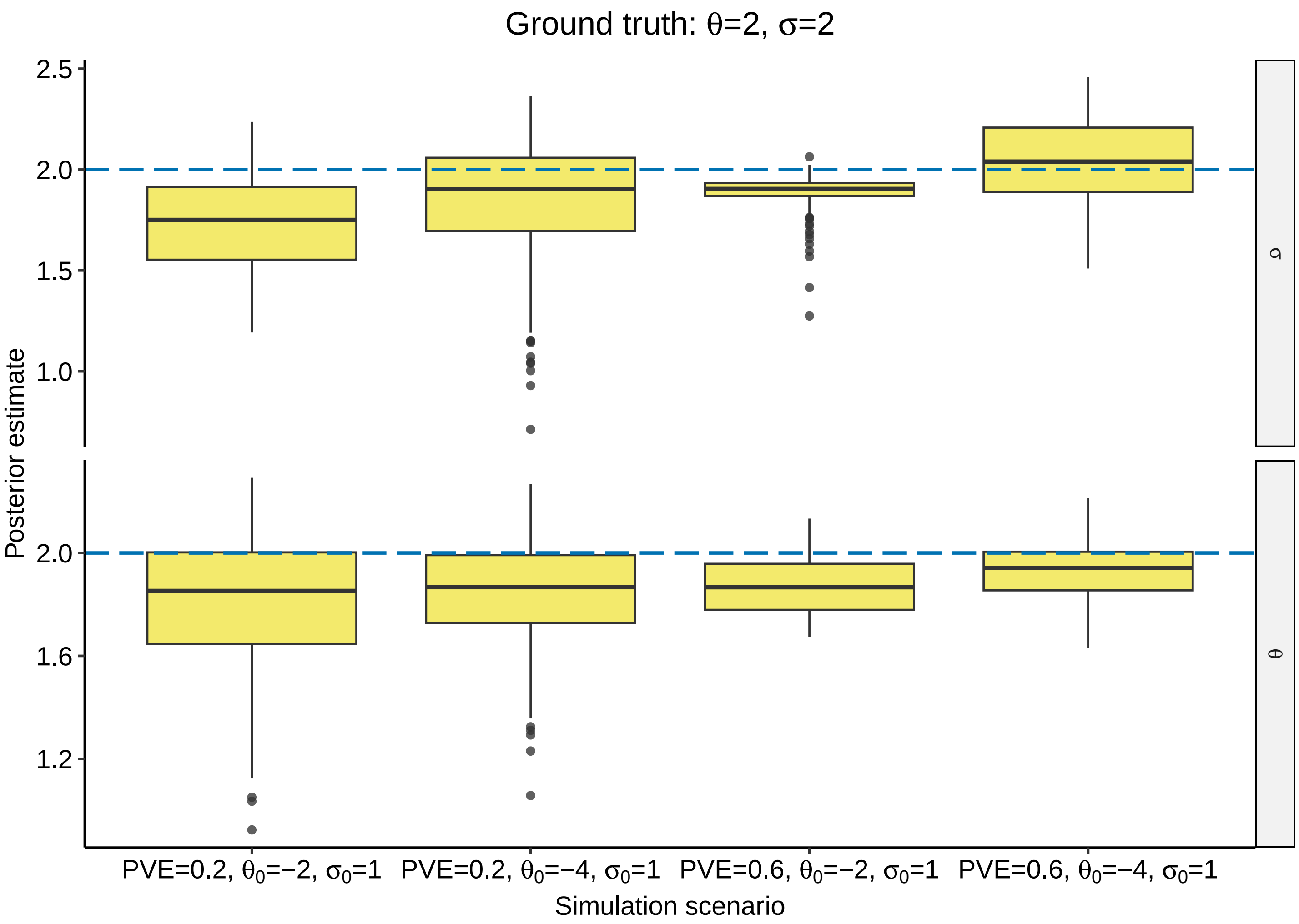

Figure S5: Posterior estimation of enrichment parameters in the RSS-NET extension across 4 simulation scenarios. The x-axis indicates the true hyper-parameter values for each simulation scenario (Note S2). The y-axis indicates the posterior mean estimates (upper: σ; lower: θ). Each box contains results of 200 independent positive datasets for a given scenario. Box plots indicate median (middle line), 25th, 75th percentile (box) and 5th and 95th percentile (whiskers) as well as outliers (single points). Horizontal dashed lines indicate true values of $\{\sigma, \theta\}$.

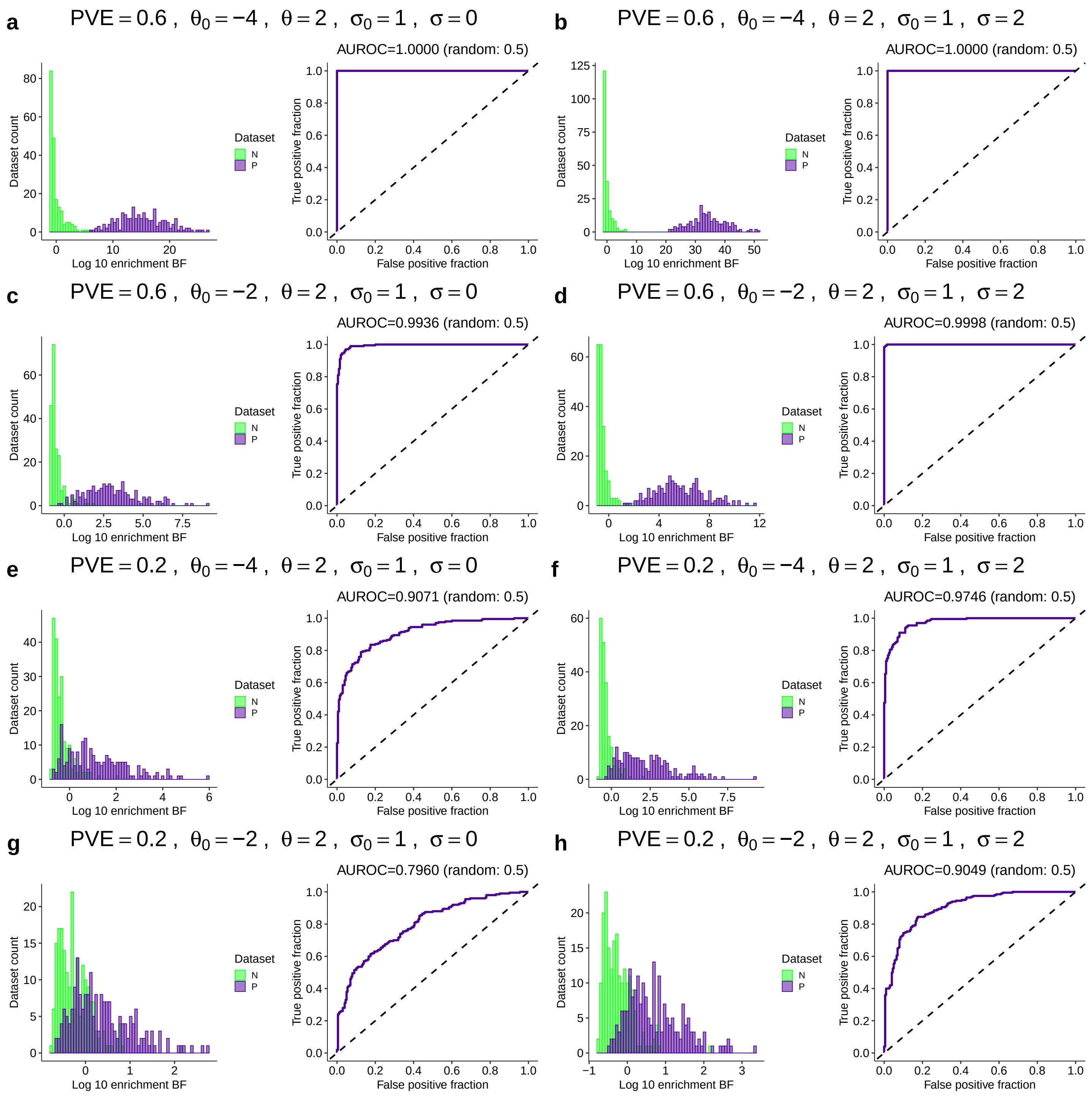

Figure S6: Trade-off between false and true enrichments identified by the RSS-NET extension across 8 simulation scenarios. The title of each panel indicates the ground truth of each simulation scenario. Each panel shows results based on enrichment Bayes factors (BFs) of 200 negative (N) and 200 positive (P) datasets simulated for a given scenario. Each dashed diagonal line has a slope of one and an intercept of zero, indicating the random ROC curve (AUROC=0.5). Higher value of AUROC indicates better performance. Simulation details are provided in Note S2.

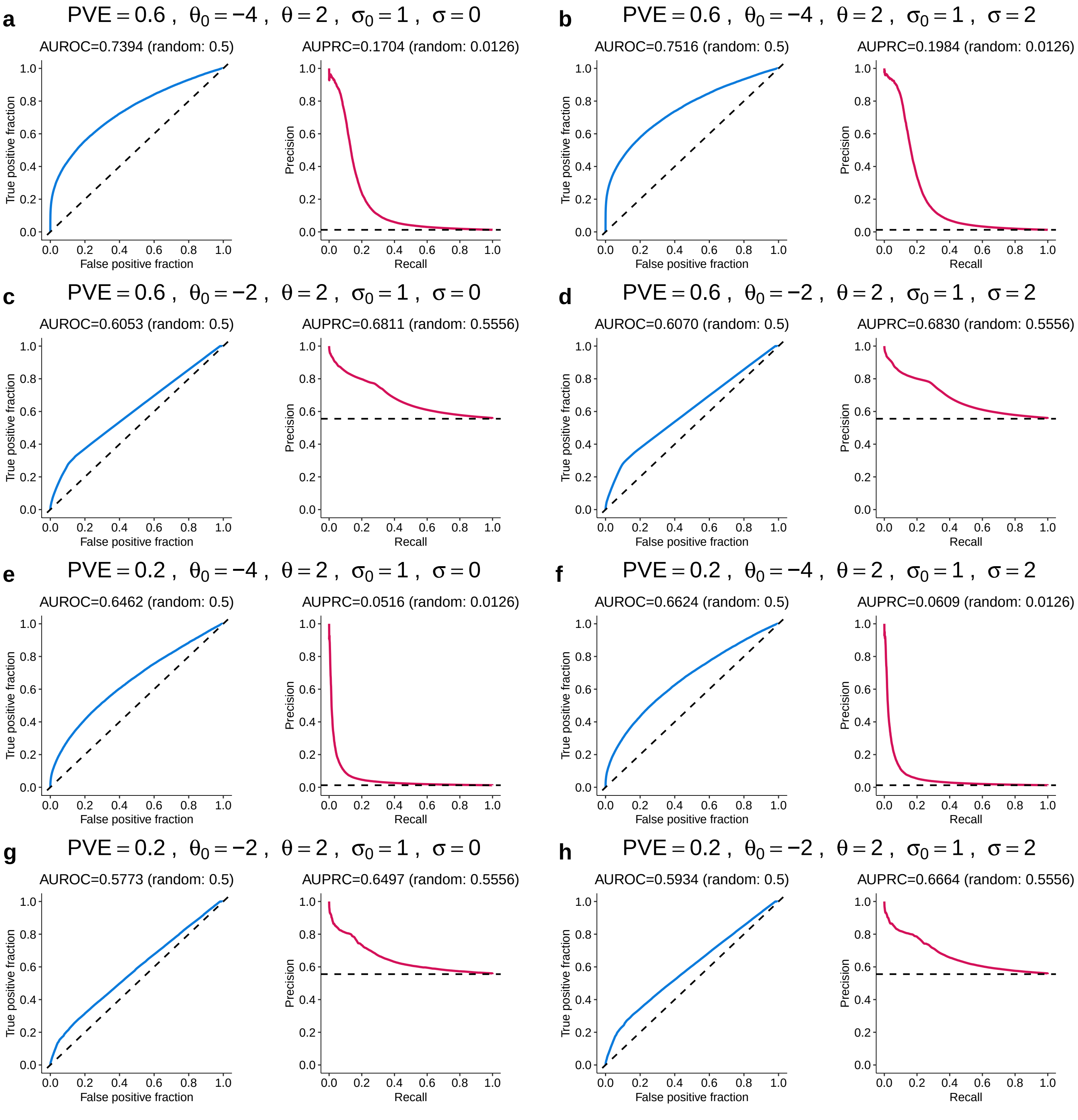

Figure S7: Trade-off between false and true associations at the level of HC ELEs identified by the RSS-NET extension across 8 simulation scenarios. The title of each panel indicates the ground truth of each simulation scenario. Each panel shows results based on 200 positive datasets simulated for a given scenario. Each dashed diagonal line has a slope of one and an intercept of zero, indicating the random ROC curve (AUROC=0.5). Each dashed horizontal line indicates the random PR curve. For both AUROC and AUPRC, higher value indicates better performance. Simulation details are provided in Note S2.

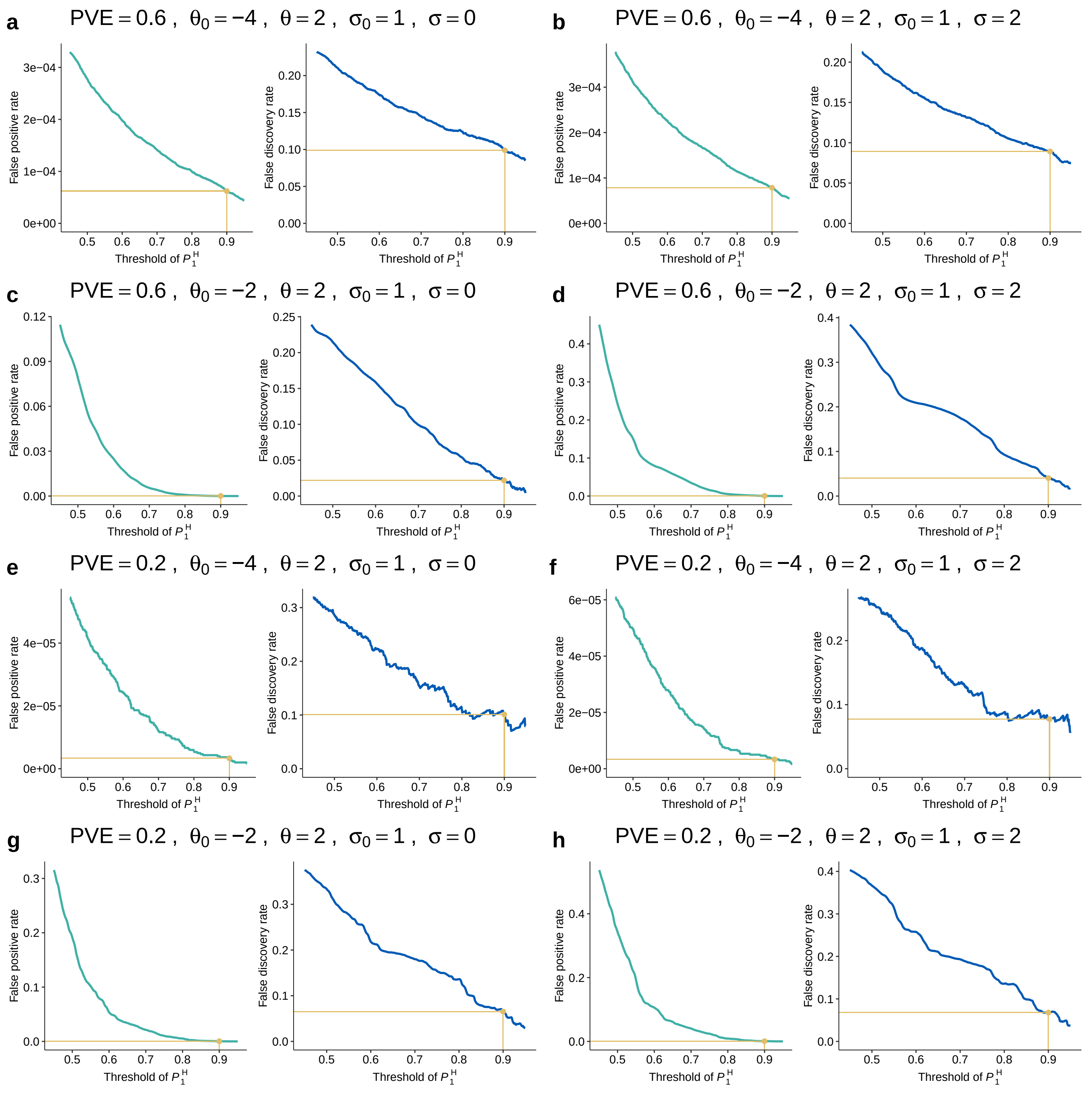

Figure S8: False positive and discovery rates for 8 simulation scenarios across a wide range of P_1^H thresholds. The false positive rate is defined as the proportion of HC ELEs that do not contain any causal SNP but reach a given significance threshold of P_1^H (i.e., false positives) among all the HC ELEs that do not contain any causal SNP. The false discovery rate is defined as the proportion of false positives among all the HC ELEs with posterior trait-associated probability under the enrichment model for HC ELEs reaching a given threshold (e.g., $P_1^H \geq 0.9$). The title of each panel indicates the ground truth of each simulation scenario (Note S2). For each of the 8 simulation scenarios, false positive and discovery rates are computed based on all 200 simulated datasets. The yellow dot in each panel indicates the false positive or discovery rate at $P_1^H \geq 0.9$ for each simulation scenario.

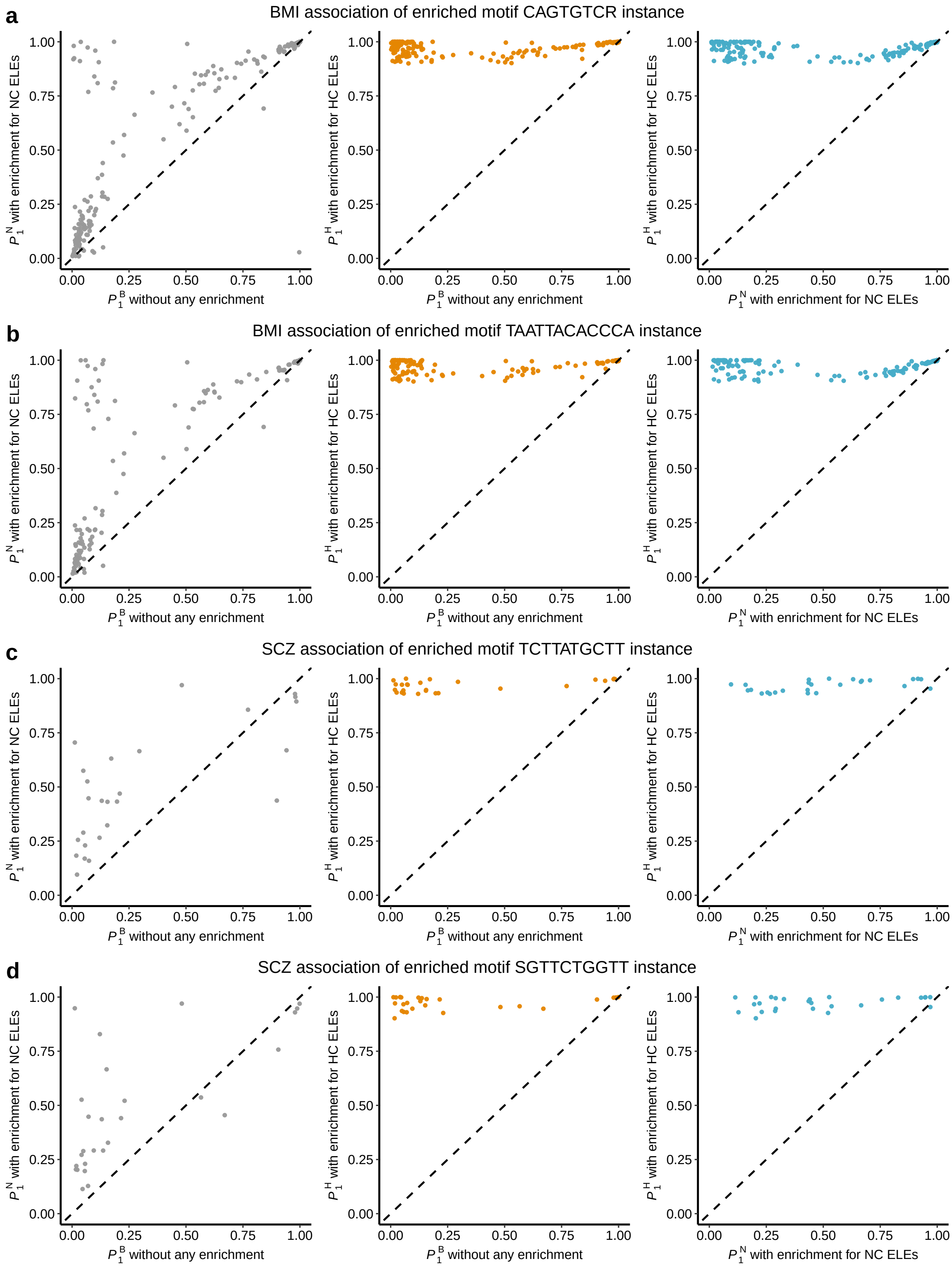

Figure S9: Comparison of posterior trait-associated probabilities under the enrichment model for HC ELEs (P_1^{H}), the enrichment model for NC ELEs (P_1^{N}), and the baseline model without any enrichment (P_1^{B}), for the same HC ELE in the same GWAS. a BMI associations of 210 HC ELEs that contain instances of the enriched motif CAGTGTCR. **b** BMI associations of 154 HC ELEs that contain instances of the enriched motif TAATTACACCCA. **c** Schizophrenia associations of 26 HC ELEs that contain instances of the enriched motif TCTTATGCTT. **d** Schizophrenia associations of 27 HC ELEs that contain instances of the enriched motif SGTTCTGGTT. In each panel, each point represents a HC ominbus ELE present in neural samples, and the dashed diagonal line has a slope of one and an intercept of zero.

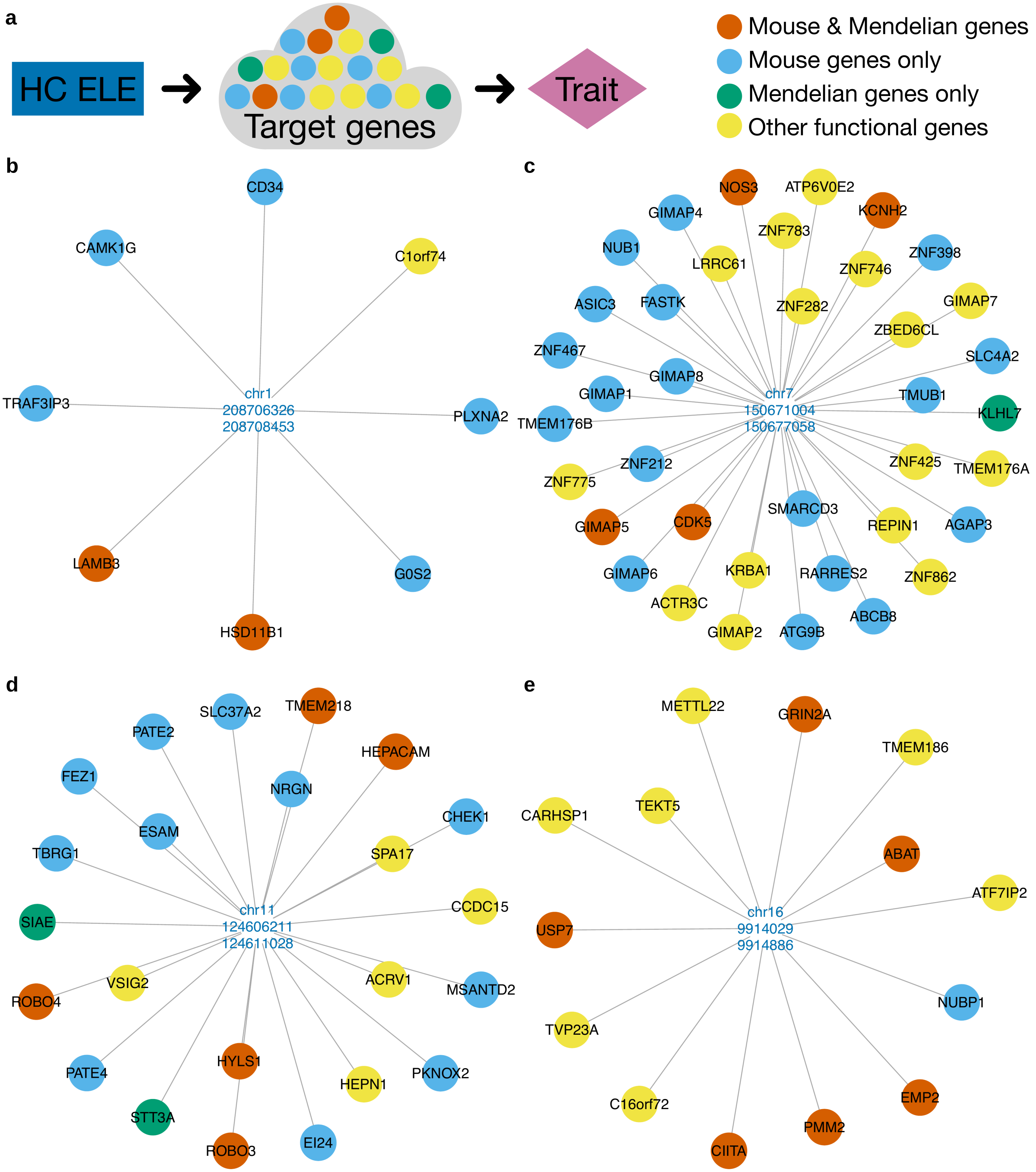

Figure S10: Putative target genes of HC ELEs associated with BMI and schizophrenia. **a** HC ELEs affect a complex trait through regulating target genes with functional impact on the trait. **b-c** Putative target genes (Table S18) of two BMI-associated HC ELEs that contain instances of the enriched motif CAGTGTCR (Fig. 5c): **b** chr1:208706326-208708453 ($P_1^{\mathrm{B}} = 0.05, P_1^{\mathrm{N}} = 0.19, P_1^{\mathrm{H}} = 1.00$); **c** chr7:150671004-150677058 ($P_1^{\mathrm{B}} = 0.05, P_1^{\mathrm{N}} = 0.04, P_1^{\mathrm{H}} = 0.99$). **d-e** Putative target genes (Table S24) of two schizophrenia-associated HC ELEs that contain instances of the enriched motif SGTTCTGGTT (Fig. 6d): **d** chr11:124606211-124611028 ($P_1^{\mathrm{B}} = 0.04, P_1^{\mathrm{N}} = 0.53, P_1^{\mathrm{H}} = 1.00$); **e** chr16:9914029-9914886 ($P_1^{\mathrm{B}} = 0.16, P_1^{\mathrm{N}} = 0.33, P_1^{\mathrm{H}} = 0.99$).

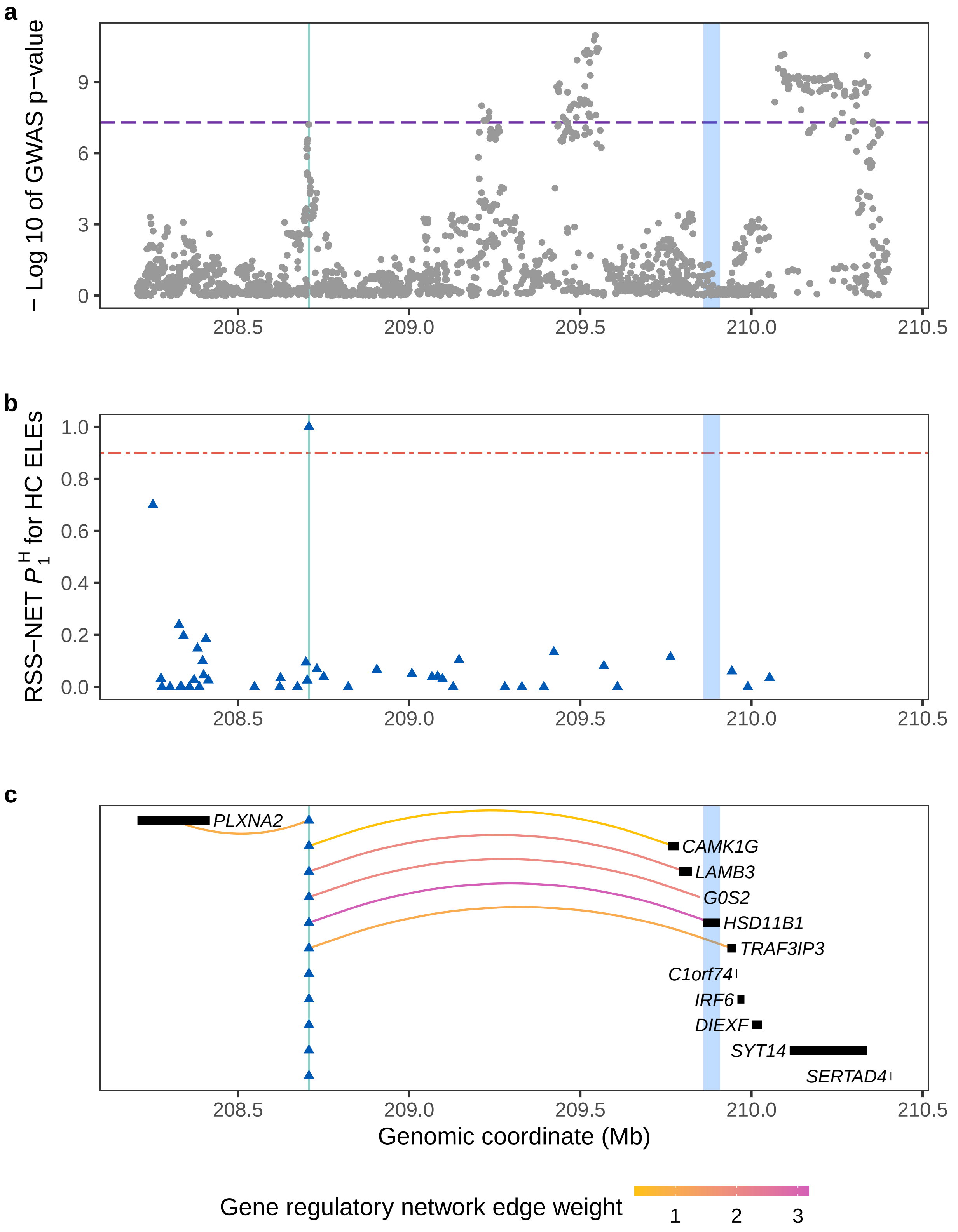

Figure S11: Regional visualization of the BMI-associated HC ELE chr1:208706326-208708453. **a** Each point represents a GWAS SNP in the given genomic region, with its GWAS P-value on the -log10 scale shown on the vertical axis and its chromosomal position shown on the horizontal axis. The purple horizontal dashed line indicates the genome-wide significance threshold ($P = 5 \times 10^{-8}$) on the -log10 scale. **b** Each triangle represents a HC ELE in the given genomic region, with its posterior trait-associated probability under the enrichment model for HC ELEs (P_1^{H}) shown on the vertical axis and its chromosomal position shown on the horizontal axis. The red horizontal dashed line indicates the significance threshold for posterior trait-associated probabilities ($P_1^{\mathrm{H}} = 0.9$). **c** Each triangle represents the same trait-associated HC ELE ($P_1^{\mathrm{H}} \geq 0.9$) identified by the RSS-NET extension in the given genomic region, and each bar represents a protein-coding gene. If the trait-associated HC ELE is linked to a gene according to a recently published gene regulatory network, then they are connected by a curved edge. The edge color corresponds to the estimated edge weight in the gene regulatory network. For **a**-**c**, the green vertical solid line indicates the trait-associated HC ELE ($P_1^{\mathrm{H}} \geq 0.9$) identified by the RSS-NET extension, and the blue rectangular area indicates the prioritized effector gene that is likely regulated by the trait-associated HC ELE and is supported by multiple lines of external evidence (Fig. S15).

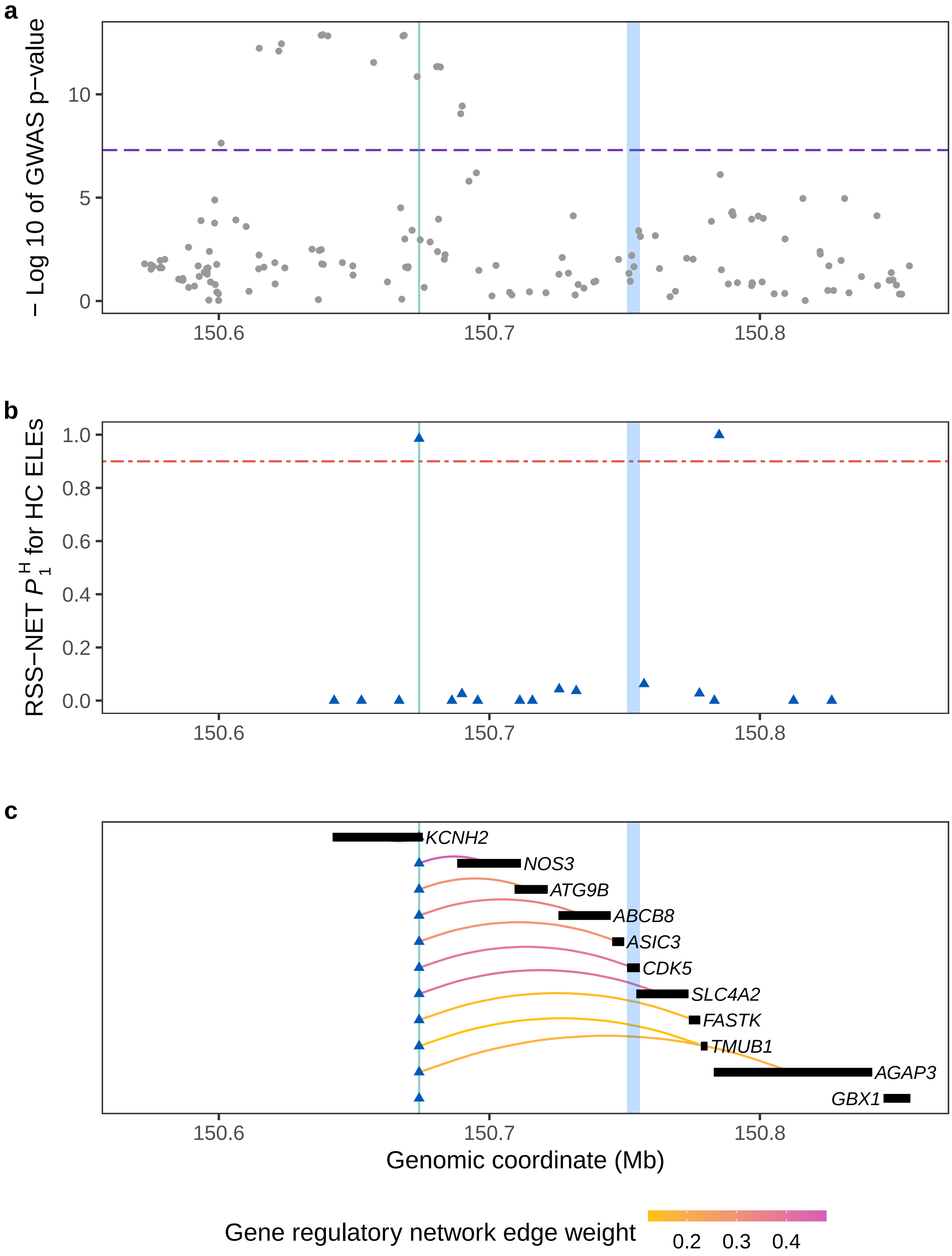

Figure S12: Regional visualization of the BMI-associated HC ELE chr7:150671004-150677058. The legend is the same as Fig. S11.

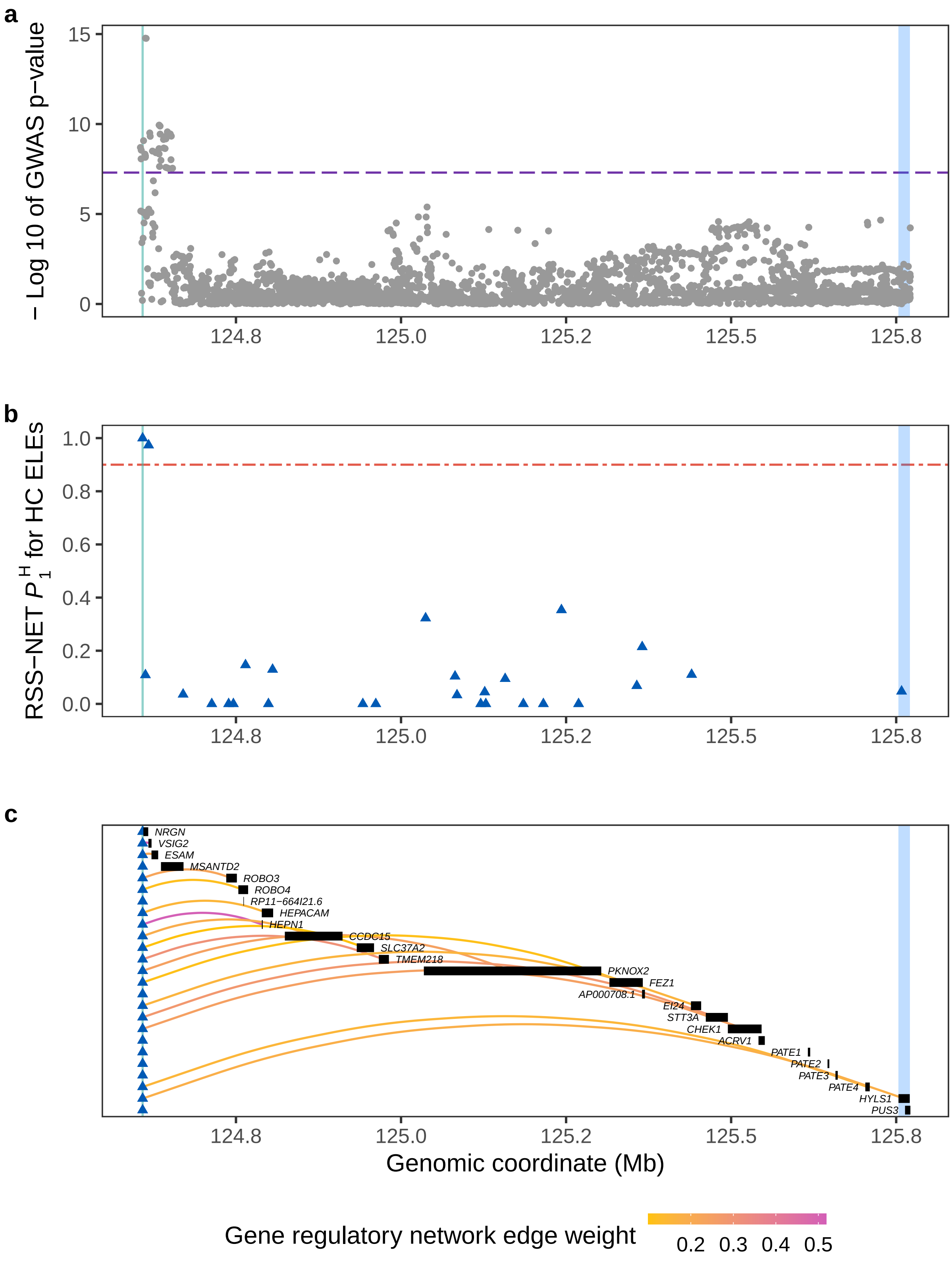

Figure S13: Regional visualization of the schizophrenia-associated HC ELE chr11:124606211-124611028. The legend is the same as Fig. S11.

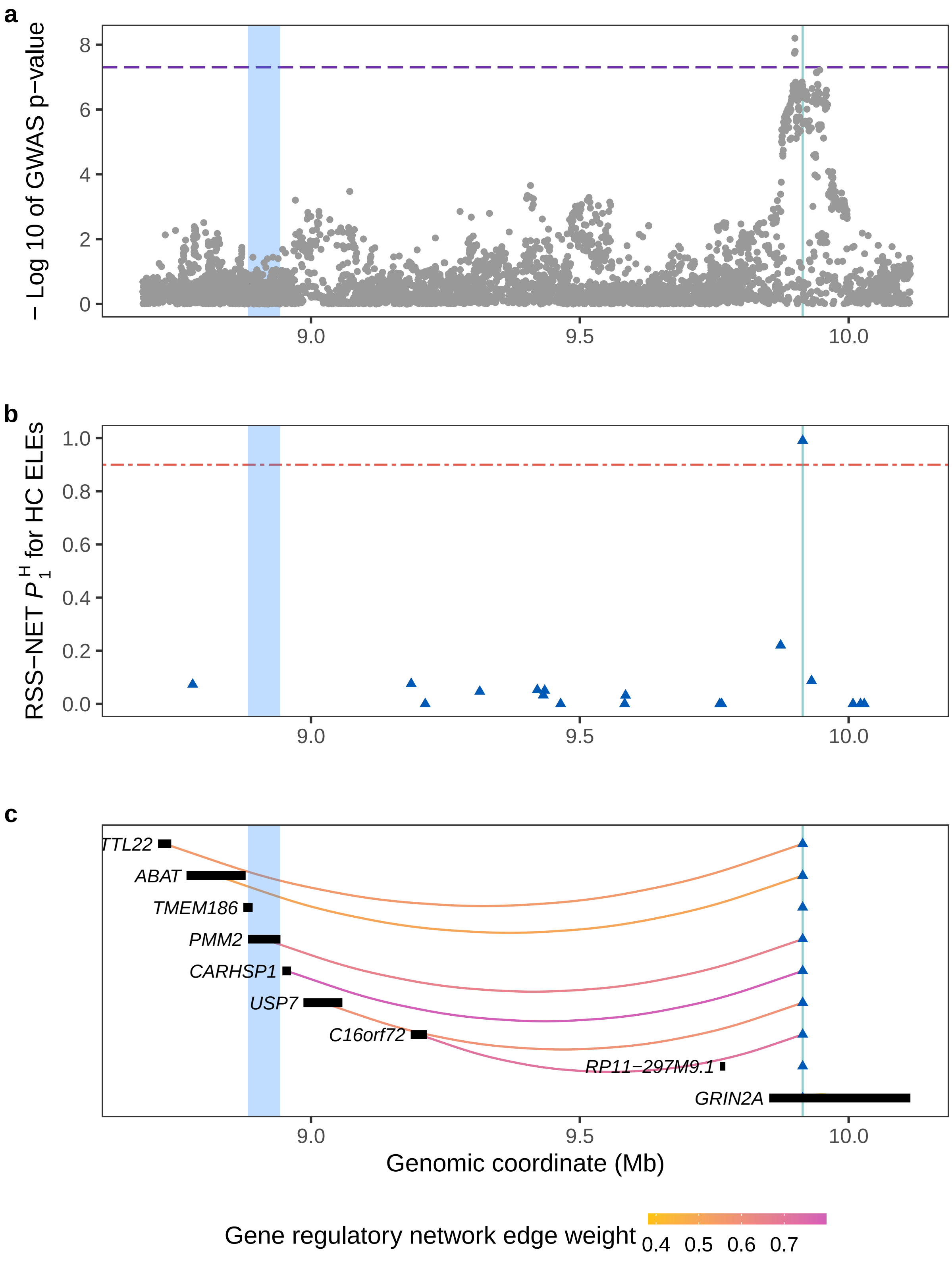

Figure S14: Regional visualization of the schizophrenia-associated HC ELE chr16:9914029-9914886. The legend is the same as Fig. S11.

Gene (trait)	Database ID	Summary
CDK5 (BMI)	MGI: 101765	Brain development<br>Learning and conditioning<br>Suckling reflex<br>Locomotor activity<br>Weakness<br>Melanogenesis<br>Hair and skin conditions<br>Cyanosis<br>Hypopnea
	OMIM: 123831	Lissencephaly 7 with cerebellar hypoplasia
	TTD: T20973	L-751250 (Preclinical, Obesity)
HSD11B1 (BMI)	MGI: 103562	Food intake and eating behavior<br>Corticosterone, insulin and lipid levels<br>Glucose tolerance<br>Abdominal fat pad<br>Subcutaneous adipose tissue<br>Weight gain
	OMIM: 600713	Cortisone reductase deficiency 2
	TTD: T65200	INCB13739 (Phase 2a, Type-2 diabetes)<br>BMS-770767 (Phase 2, Hypercholesterolemia)<br>JTT-654 (Phase 2, Type-2 diabetes)<br>RG-4929 (Phase 2, Metabolic disorder)<br>UE-2343 (Phase 2, Alzheimer disease)<br>URSOLIC ACID (Phase 2, Metabolic syndrome)<br>AZD8329 (Phase 1, Obesity)<br>BI-135585 (Phase 1, Type-2 diabetes)<br>BMS-816336 (Phase 1, Lipid metabolism disorder)<br>HPP-851 (Preclinical, Metabolic disorder)
HYLS1 (SCZ)	MGI: 1924082	Prepulse inhibition<br>Heart looping<br>PQ interval<br>Tibia length<br>Embryonic growth
	OMIM: 610693	Hydrolethalus syndrome
PMM2 (SCZ)	MGI: 1859214	Neuroepithelium<br>Myocardium layer<br>Interventricular septum<br>Ventricular wall<br>Hemorrhage<br>Body length and weight<br>Embryonic growth
	OMIM: 601785	Congenital disorder of glycosylation type Ia

Figure S15: External database lookup of select downstream genes of BMI and schizophrenia-associated HC ELEs. BMI: body mass index. SCZ: schizophrenia. MGI: Mouse Genome Informatics. OMIM: Online Mendelian Inheritance in Man. TTD: Therapeutic Target Database. The lookups of all putative target genes are provided in Tables S22 and S25.

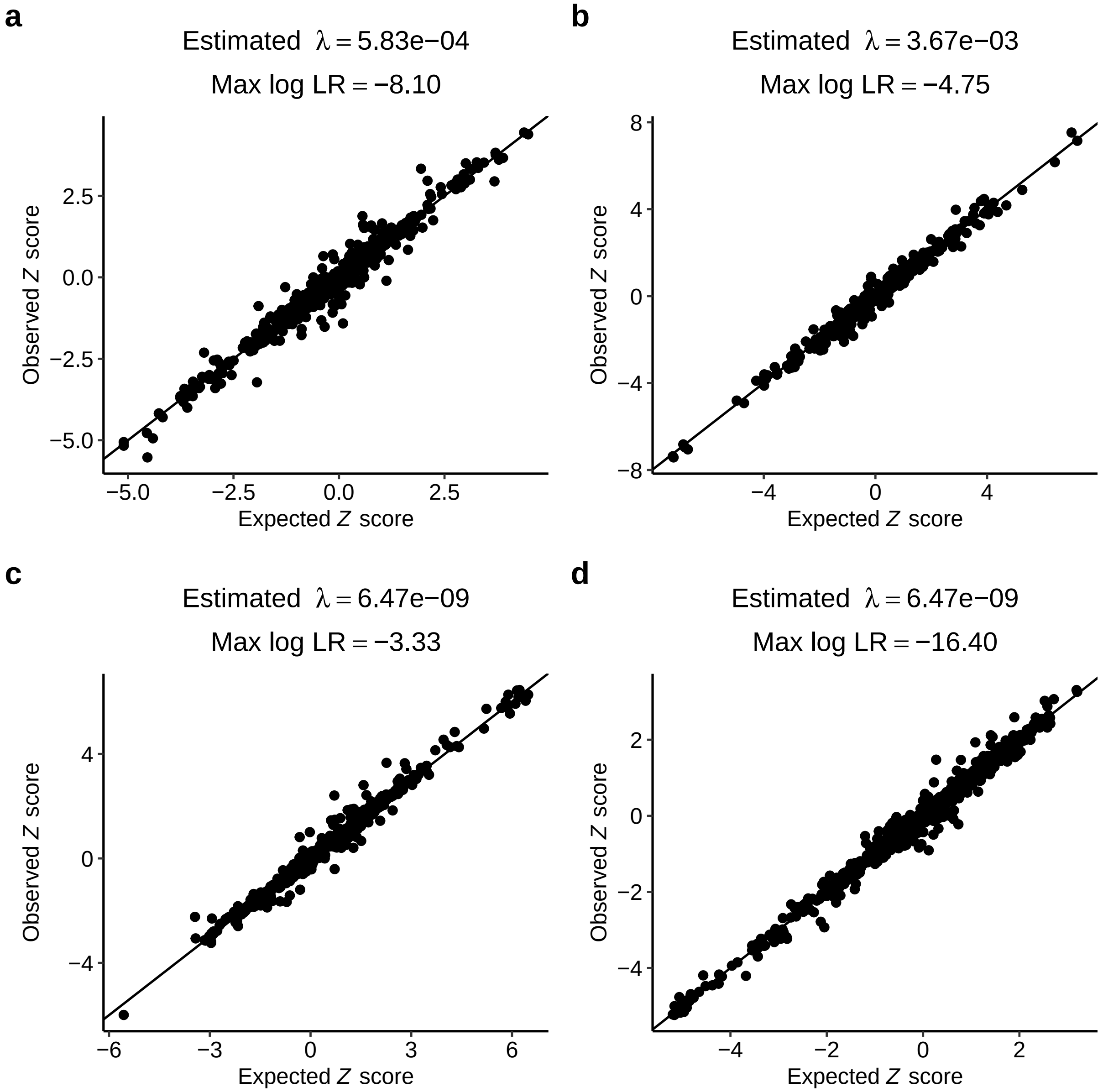

Figure S16: Consistency between GWAS summary statistics and reference LD estimates. We perform the consistency check at four loci, each of which is defined as the ±500 kb region around a trait-associated HC ELE prioritized for either BMI (Fig. 5) or schizophrenia (Fig. 6). For each locus, we estimate a regularization parameter $\lambda \in [0,1]$ as $\hat{\lambda} = \arg\max_{\lambda} \mathcal{N}(\mathbf{z};\mathbf{0},(1-\lambda)\mathbf{R}+\lambda\mathbf{I})$, where $\mathbf{z}$ is the vector of observed GWAS Z scores for all SNPs in a given locus, $\mathbf{R}$ is the LD matrix for these SNPs estimated from the reference panel, and $\mathbf{I}$ is an identity matrix that matches the dimension of $\mathbf{R}$. The estimated $\hat{\lambda}$ reflects the consistency between the GWAS summary statistics ($\mathbf{z}$) and the reference LD matrix ($\mathbf{R}$) – if $\mathbf{z}$ and $\mathbf{R}$ are consistent with one another, $\hat{\lambda}$ will be close to zero. For each locus, we also compute a likelihood ratio (LR) statistic for each SNP to assess whether its alleles in GWAS are coded differently from the reference LD (a.k.a. "allele flip"), and we report the maximum LR value among SNPs with observed GWAS Z scores greater than two in magnitude (i.e., $|Z| > 2$). If a SNP does not have an allele flip, the estimated LR will be less than one (i.e., $\log$ LR < 0). Additionally, we compute the expected Z score of each SNP conditional on all other SNPs in a given locus, and then compare it with the observed GWAS Z score of the same SNP. A large difference between the expected and observed Z scores of the same SNP indicates a potential allele flip. We use the R package susieR to produce diagnostic statistics and plots. Specifically, we use the 'estimate_s_rss' function to estimate λ for each locus, and we use the 'kriging_rss' function to compute the expected Z score of each SNP conditional on all other SNPs in each locus, as well as the LR statistic of SNP for detecting allele flips. **a** BMI-associated HC ELE chr1:208706326-208708453. **b** BMI-associated HC ELE chr7:150671004-150677058. **c** schizophrenia-associated HC ELE chr11:124606211-124611028. **d** schizophrenia-associated HC ELE chr16:9914029-9914886. The diagonal line in each panel has slope 1 and intercept 0. For all four loci, the $\hat{\lambda}$ values are close to zero, the maximum LR values are less than one, and the expected and observed Z scores align across all SNPs, thus confirming the consistency between the GWAS summary statistics and the reference LD estimates.
