# Supplementary material for: Genetic effects of sequence-conserved enhancer-like elements on human complex traits
Source: Genome Biol. 2024 Jan 2;25:1. doi: 10.1186/s13059-023-03142-1 (PMC10759394; doi:10.1186/s13059-023-03142-1)
Supplement: Supplementary file 3 — Additional file 3. Review history. [file 13059_2023_3142_MOESM3_ESM.docx]

**Review history**

**First round of review**

**Reviewer 1**

In this manuscript, the authors present an analysis strategy based on combining functional genomics data in human tissues and cell types with sequence conservation between the mouse and human genomes. Specifically, they combine putative regulatory elements defined in this way (conserved AELEs) with GWAS findings, and report context-specific enrichments of heritability and causal variants for this subset of genomic regions. The authors compare these findings with those obtained with genomic regions defined by human-mouse comparisons of epigenomic data (without a sequence conservation filter). Lastly, the manuscript applies the strategy outlined by the authors to prioritise and annotate trait-associated regulatory elements, with a focus on fine-mapping variants in BMI and schizophrenia.
Overall, the authors present a detailed and interesting analysis based on leveraging of both functional genomics datasets in human and human-mouse sequence conservation. However, in its present form we have a number of concerns on the presentation and interpretation of the results, which we detail below.

Major concerns:
1) As presented, we find the novelty of the approach taken by the authors to be overstated. The use of human-mouse sequence conservation as a way to prioritise GWAS findings is well established in human disease genetics and has been used in many previous studies. Moreover, the use of human-to-mouse sequence conservation and its combination with functional genomics data (in either human or mouse) has informed a number of large-scale initiatives such as the mouse ENCODE project. In this regard, the manuscript introduction could be better balanced in acknowledging seminal studies establishing the concept of sequence conservation as a tool to prioritise epigenomically-defined enhancers (e.g. PMID 20729851, 24360275), and the contribution of previous work to the strategy taken by the authors. Similarly, the Discussion should acknowledge similar approaches and their application to GWAS annotation (such as PMIDs 32579926, 36817700), and include a more detail consideration on whether the prioritisations proposed on Figures 5 and 6 for BMI and schizophrenia substantially add to those proposed in recent post-GWAS studies of these traits).

2) The nomenclature and organisation of the manuscript could be substantially improved for clarity and readability. The use of 'active enhancer-like elements' should be clarified. It is unclear if or how promoter regions were distinguished from enhancers as both types of regulatory elements would exhibit H3K27ac and open chromatin. For example, the concept of promoter H3K27ac signal is obviated on page 3 line 89. Given that promoter and enhancer regions can have substantially different levels of sequence conservation, the authors should clarify to what extent their chosen nomenclature is effectively enriching for promoter regions in some of the conservation categories used in the manuscript.
Further to this, a little more caution should be exercised around the classification of the regulatory elements as active (particularly as no functional validation is included in the manuscript). Line 3, 91 to 93: there is a contradiction in the statement that "biochemical activities are not necessarily definite proof of function", but within same sentence the authors class the putative elements that they identify as "active enhancer-like elements".
More generally, the manuscript text and organisation should be improved for clarity and readability:
- Many of the figures and figure legends should be revised for consistency and clarity (for instance, see Figure 1i axis labels and the description in the figure legend).
- Some sections of the manuscript text are difficult to follow with jumbled terminology ("functional annotations", "SNP annotations", "existing annotations"). The manuscript would benefit from some revision in this area, with use of clear definitions in the main text and consistent terminology.

3) The discussion of enhancer and promoter regions selected based on epigenomic conservation of H3K27ac signals (page 11, line 300-316) is useful to include in the manuscript, but we found the results presented here are poorly described (as is their contextualisation in the Discussion). The analysis presented by the authors seemingly compares regulatory regions identified on conservation of chromatin accessibility and H3K27ac signals in human and mouse (which the authors term "H3K27ac-conserved AELEs" and compared them with regions of high sequence conservation and H3K27ac/chromatin accessibility signals in human ("HC AELEs"). This analysis strategy is biased towards the authors approach and interpretation, in that comparison of high conservation AELEs are compared to "H3K27ac-conserved AELEs" is incomplete. AELEs defined by the authors are heterogeneous in their sequence conservation levels (Figures 1 and 2), as are "H3K27ac-conserved AELEs". A more nuanced analysis should also compare "like-for-like" subsets of AELEs and H3K27ac-conserved AELEs matched for their level of sequence conservation.
The discussion of the "H3K27ac conservation" approach is similarly imprecise in the Discussion section (lines 510-519). Such approaches, as cited by the authors, were often not designed as a post-GWAS annotation approach and have substantial value in answering a number of questions unrelated to the manuscript's. Therefore their discussion could be more balanced, and acknowledge the wider context in which many of these studies were undertaken.
Moreover, the authors could consider benchmarking their approach against existing methods of post-GWAS prioritisation (for instance integrating epigenomic and gene expression data without the use of sequence conservation).

Minor comments:

Page 21, line 560-562; The description for 'NC' categorisation could be more explicit to remove any disambiguation as "to identify AELEs without sequence conservation…" could imply conservation was not considered in the approach, i.e. all putative elements are included. However, "NC" and no conservation could also indicate all elements where sequence was not mapped between humans and mouse (i.e. "human-specific" in this two-way comparison).

Page 4, figure 1: The readability of this figure could be improved. For example, the relatively small palette of colours is carried through the figure without necessarily retaining context, for example panel 1a yellow denoting H3K27ac vs the rest of the figure where yellow is NC. Fig 1e label could be interpreted as DNase-CTCF and Fig 1h font is very small.

Page 19 line 498 spelling error

Page 20 line 525 spelling error

**Reviewer 2**

The manuscript by Zhu and colleagues studied how conservation of regulatory elements may be used to refine our understanding of genetics of complex traits. It shows that highly conserved elements (AELEs) are more enriched with heritability of traits, and with fine-mapped variants than general AELEs. This finding motivated the authors to develop a Bayesian statistical method to integrate AELE annotations with GWAS summary statistics to identify AELEs contributing to genetics of complex traits. The results from applying this approach to BMI and Schizopheria (SCZ) were discussed in details, with some novel genes highlighted.

Overall, this is a well-written paper. It provided systemtic evidence that HC regulatory sequences are important sources of information for human genetic studies. Furthermore, it provided a statistical method for researchers to leverage such information. Additionally, the results in the paper, including the novel risk genes of BMI and SCZ, would be good resources to the community. As such, I think the paper is of good interest to the broad audience of Genome Biology. Below are my specific comments:

Major comments
1) The authors extended their earlier model, RSS-Net, in this paper. The details of the new method, however, are relatively sparse. There are few details about how the inference works, and no results to assess the method (e.g. simulation studies that often accompany new methods). The new method in fact differs quite a bit from the original RSS-Net. It was designed to solve a different biological problem, and the two differ in techincal aspects. In the original RSS-Net, causal variants have the same prior effect size, while in the new model, the prior effect sizes of variants depend on annotations. Given these differences, more details/justification of the new method may be necessary.

One particular concern is how to interpret P1, the metric the authors used to assess relevance of AELEs to the phenotypes. Given the sparse prior used in the model, it seems that the method is trying to perform fine-mapping, with a prior that favors HC AELEs. If this is the case, then P1 can be intepreted as as the probability that an AELE contains one or more causal variant. But this point is never explicitly mentioned. If this is indeed the goal here, then as in any fine-mapping analysis, it would be important to know if P1 is calibrated. Ex. at P1 > 0.9, are the proportion of false AELEs (those containing no causal variants) below 10%? This can be assessed through simulation studies.

2) Related to this point is the discovery of many HC AELEs for BMI and SCZ. The results of SCZ are particularly striking: the paper reported 173 HC AELEs at P1 > 0.9 using HC AELEs as the prior, compared to just 19 using a background (uniform) prior. The difference appears to be somewhat too large. For example, in the PolyFun paper [33199916], the increase of the number of fine-mapped variants by using functional prior is much more modest, about 30-50%. This raises the concern of calibration as pointed above. What explains the large difference of power from using HC AELE prior? What are the values of the hyperparameters learned from data? It would be useful to show some example(s) where the prior makes a difference. This would give the readers some idea of how the method finds new AELEs that would have been missed.

3) The authors reported some novel candidate genes for BMI and SCZ. Again, it is unclear how the new method found these genes, while earlier efforts miss them. Is this due to the better power of "fine-mapping" HC AELEs? Or are these genes far from the GWAS lead variants, so that they were missed previously but now were discovered because of the use of distal interaction data? It would be helpful to show one or two figure to provide the genetic evidence supporting the HC AELEs (GWAS p-values, P1), as well as genomic data linking the AELEs with the genes.

Minor comments

- Definition of HC, MC and LC elements: are these classes disjoint? It seems that they are not, based on the numbers. But the readers have to infer that point. Given that these classes are really fundamental to the paper, it would be helpful to clarify this in the main text.

- In the text about Fig. 1i (the comparison of HC AELE with other annotations): what does V mean? The figure legend says "correlation". It's better to use consistent terms here.

- The extended RSS-Net method in the paper seems quite similar to the bfGWAS method (PMID:28844487). It should be cited, and perhaps discussed.

- Linking enhancers to genes: the authors cited a few studies, but there are few details of how this is done. Are these based on Hi-C data, or cis-eQTLs or other methods? What specific datasets are used? Ex. Supplementary Table(s) in the original studies. Such details need to be provided for the study to be reproducible.

- Fig. 5 legend say, "BMI associations of c". This looks confusing. One would usually put Panel number first, then text.

**Authors’ response to reviewers**

Point-by-point Responses to Reviewer Comments (GBIO-D-22-01305)

Xiang Zhu <[xiangzhu@psu.edu](mailto:xiangzhu@psu.edu)>

We sincerely thank the reviewers for their positive evaluations and constructive comments on our manuscript **GBIO-D-22-01305** entitled "*Genetic effects of sequence-conserved enhancer- like elements on human complex traits*". We have carefully considered all comments and revised our manuscript accordingly. Below, we provide the point-by-point responses to all comments.

Reviewer comments are shown in blue Arial font, and our responses are shown in black. All changes in the revised manuscript are underlined in blue. All display items, page numbers, and line numbers referenced below correspond to the revised manuscript.

# Reviewer #1 Comments

**Reviewer #1**: In this manuscript, the authors present an analysis strategy based on combining functional genomics data in human tissues and cell types with sequence conservation between the mouse and human genomes. Specifically, they combine putative regulatory elements defined in this way (conserved AELEs) with GWAS findings, and report context-specific enrichments of heritability and causal variants for this subset of genomic regions. The authors compare these findings with those obtained with genomic regions defined by human-mouse comparisons of epigenomic data (without a sequence conservation filter). Lastly, the manuscript applies the strategy outlined by the authors to prioritise and annotate trait-associated regulatory elements, with a focus on fine-mapping variants in BMI and schizophrenia.

We thank Reviewer #1 for the summary of our work. This summary is largely accurate, except for the statement "*The authors compare these findings with those obtained with genomic regions defined by human-mouse comparisons of epigenomic data (without a sequence conservation filter).*" In our original manuscript, "a sequence conservation filter" was actually used so that all comparisons were made at the same level of sequence conservation. To ensure this key detail is clearly conveyed to the readers, we have revised our manuscript accordingly. The details can be found in our responses to Reviewer #1's specific comment related to this analysis.

Overall, the authors present a detailed and interesting analysis based on leveraging of both functional genomics datasets in human and human-mouse sequence conservation. However, in its present form we have a number of concerns on the presentation and interpretation of the results, which we detail below.

We appreciate that Reviewer #1 finds our work "detailed and interesting". We also thank Reviewer #1 for all the constructive and inspiring comments provided, each of which has now been addressed in full. Below are our detailed responses and revisions point by point.

**Major concerns**:

1) As presented, we find the novelty of the approach taken by the authors to be overstated.

We greatly appreciate the useful comments from Reviewer #1. As detailed below, we have carefully revised our manuscript so that "the novelty of the approach" is now accurately stated.

The use of human-mouse sequence conservation as a way to prioritise GWAS findings is well established in human disease genetics and has been used in many previous studies. Moreover, the use of human-to-mouse sequence conservation and its combination with functional genomics data (in either human or mouse) has informed a number of large-scale initiatives such as the mouse ENCODE project. In this regard, the manuscript introduction could be better balanced in acknowledging seminal studies establishing the concept of sequence conservation as a tool to prioritise epigenomically-defined enhancers (e.g. PMID 20729851, 24360275), and the contribution of previous work to the strategy taken by the authors.

We thank Reviewer #1 for this insightful comment, as well as the relevant references and specific suggestions to improve the Introduction of our manuscript. In response, we have introduced the following revisions to fully address this comment.

- **Page 2, Lines 55-58**: "*Inspired by previous efforts of combining evolutionary and biochemical approaches to prioritize regulatory sequences in mammalian genomes [14, 15], recent studies have adopted this concept to interpret non-coding variation underlying human traits.*" **Ref.s 14-15** are suggested by Reviewer #1.
- **Page 3, Lines 76-81**: "*Here, we revisit the classical idea of exploiting human-mouse sequence conservation to locate functional elements in the human genome [28, 29], which has informed many large-scale initiatives [9, 30]. Building on this simple but profound idea, we develop a human-mouse comparison method to identify human enhancer-like elements that display both sequence conservation and biochemical activity.*" **Ref. 30** is suggested by Reviewer #1.
- **Page 30, Lines 885-886**: "*[14] Blow, M. J. et al. ChIP-Seq identification of weakly conserved heart enhancers. Nat. Genet. 42, 806–810 (2010).*" This is the relevant publication PMID: 20729851 suggested by Reviewer #1.
- **Page 30, Lines 887-888**: "*[15] Nord, A. S. et al. Rapid and pervasive changes in genome-wide enhancer usage during mammalian development. Cell 155, 1521–1531 (2013).*" This is the relevant publication PMID: 24360275 suggested by Reviewer #1.
- **Page 31, Lines 924-925**: "*[30] Yue, F. et al. A comparative encyclopedia of DNA elements in the mouse genome. Nature 515, 355–364 (2014).*" This is the flagship publication for the mouse ENCODE project suggested by Reviewer #1.

The newly added text and citations lead to a more accurate and balanced Introduction section in acknowledging previous studies that are relevant to the background and approach of our work.

Similarly, the Discussion should acknowledge similar approaches and their application to GWAS annotation (such as PMIDs 32579926, 36817700),

We thank Reviewer #1 for providing the relevant references and the useful suggestions to improve the Discussion section. To fully address this comment, we have made the following additions in our revised manuscript.

- **Page 22, Lines 601-603**: "*Our findings, together with recent studies by others [20-22, 26, 84, 85], emphasize the importance of combining evolutionary and biochemical evidence to understand the regulatory basis of heritable human traits.*" **Ref.s 84-85** are suggested by Reviewer #1.
- **Page 33, Lines 1047-1048**: "*[84] Castelijns, B. et al. Recently evolved enhancers emerge with high interindividual variability and less frequently associate with disease. Cell Rep. 31, 107799 (2020).*" This is the relevant publication PMID: 32579926 suggested by

Reviewer #1.

- **Page 33, Lines 1049-1050**: "*[85] Destici, E. et al. Human-gained heart enhancers are associated with species-specific cardiac attributes. Nat. Cardiovasc. Res. 1, 830–843 (2022).*" This is the relevant publication PMID: 36817700 suggested by Reviewer #1.

With the newly added text and citations, we anticipate that the readers will find our manuscript accurate and balanced in acknowledging relevant studies.

and include a more detail consideration on whether the prioritisations proposed on Figures 5 and 6 for BMI and schizophrenia substantially add to those proposed in recent post-GWAS studies of these traits).

We thank Reviewer #1 for this valuable comment. As requested, we have performed an in-depth lookup of the following four relevant publications, which are cited in our manuscript.

- PMID: 30124842 (**Ref. 39, Page 31, Lines 944-946**) provides the BMI GWAS data for us to generate the post-GWAS results in **Fig. 5**.
- PMID: 29483656 (**Ref. 40, Page 31, Lines 947-949**) provides the schizophrenia GWAS data for us to generate the post-GWAS results in **Fig. 6**.
- PMID: 36581621 (**Ref. 45, Page 31, Lines 960-961**) is the latest BMI GWAS publication with the largest sample size since the submission of our manuscript.
- PMID: 35396580 (**Ref. 72, Page 32, Lines 1019-1020**) is the latest schizophrenia GWAS publication with the largest sample size since the submission of our manuscript.

After this detailed investigation, we have confirmed that our findings in the **Fig.s 5-6**, in particular the candidate effector genes prioritized for BMI (*CDK5*, *HSD11B1*) and schizophrenia (*HYLS1*, *PMM2*), have not been identified by the same GWAS data used in our analysis, nor by the updated GWAS with much larger sample sizes, nor by the multi-omics integrative analysis of these GWAS data at the time of our study. Consequently, we believe that the findings and approaches in the present manuscript represent a major advance for the post-GWAS studies of BMI and schizophrenia. For the convenience of the reviewers, we summarize our lookup results in the following two tables. The label "No" indicates that an approach from a previous study did not identify the gene highlighted in our study.

**Lookup of BMI GWAS and post-GWAS**

| Source | Approach | *CDK5* | *HSD11B1* |
| --- | --- | --- | --- |
| PMID: 30124842, the same BMI GWAS used in our analysis | | | |
| 110_genes_prioritised_with_SM R_for_BMI_Yengo_et_al_HMG  _2018.txt | Summary-data-based Mendelian randomization (SMR) analysis (PMID: 27019110) with expression quantitative trait loci (eQTL) data from multiple human tissues (PMID: 29022597) and methylation QTL data in peripheral blood (PMID: 30514905) | No | No |
| PMID: 36581621, the latest BMI GWAS since the submission of our manuscript | | | |
| Supplementary Data 1 | GWAS meta-analysis | No | No |

**Lookup of schizophrenia GWAS and post-GWAS**

| Source | Approach | *HYLS1* | *PMM2* |
| --- | --- | --- | --- |
| PMID: 29483656, the same schizophrenia GWAS used in our analysis | | | |
| Supplementary Tables 3, 4, 5, 14 | GWAS meta-analysis | No | No |
| Supplementary Table 11 | Fine-mapping analysis of GWAS loci (PMID: 26773131) | No | No |
| Supplementary Table 12 | Fine-mapping analysis (PMID: 26773131) with human brain Hi-C data (PMID: 27760116) | No | No |
| Supplementary Table 13 | SMR analysis (PMID: 27019110) with human brain eQTL data (PMID: 27668389) | No | No |
| PMID: 35396580, the latest schizophrenia GWAS since the submission of our manuscript | | | |
| Extended Data Table 1 | Integrated gene prioritization based on fine-mapping (PMID: 26773131), SMR (PMID: 27019110), and rare  coding variants (PMID: 35396579) | No | No |
| Supplementary Tables 3, 24, 25 | GWAS meta-analysis | No | No |
| Supplementary Tables 11, 13, 14 | Fine-mapping analysis of GWAS loci (PMID: 26773131) | No | No |
| Supplementary Tables 12, 16, 17 | SMR analysis (PMID: 27019110) with human eQTL data from whole blood (PMID: 34475573), adult brain (PMID: 30545856) and fetal brain (PMID: 30419947) | No | No |
| Supplementary Table 18 | SMR analysis (PMID: 27019110) with human brain Hi-C data (PMID: 30545857) | No | No |
| Supplementary Table 19 | Synaptic location and function analysis of prioritized genes in the SynGO database (PMID: 31171447) | No | No |
| Supplementary Table 20 | MAGMA gene set enrichment analysis (PMID: 25885710) | No | No |

Based on this in-depth lookup of relevant publications, we have added the following paragraph in the Discussion of the revised manuscript to address the Reviewer #1' comment in full.

**Page 20, Lines 522-544**: "*Integrating HC ELEs with GWAS and gene regulatory networks further helps pinpoint previously undescribed but functionally relevant genes for BMI (e.g., CDK5, HSD11B1) and schizophrenia (e.g., HYLS1, PMM2). Despite convergent evidence supporting their roles in the biology of BMI and schizophrenia, these genes prioritized by our approach were not identified by the same GWAS data used in our analysis, nor by the updated GWAS with much larger sample sizes, nor by the multi-omics integrative analysis of these GWAS data at the time of this study [39, 40, 45, 72, 73]. Identifying these genes post-GWAS presents two challenges. First, although CDK5 and HSD11B1 are within the loci (+/-500 kb) of known GWAS hits of BMI (rs2907948, P=1.3e-13; rs12140373, P=7.7e-11), these loci contain many protein-coding genes (28 and 10, respectively), thus complicating the nomination of likely causal genes. Our approach helps address this challenge by shortlisting genes on the same regulatory circuit as HC ELEs that are strongly associated with the GWAS trait (*𝑃^!^ ≥0.9*), based on the premise that enhancers affect a trait through their downstream genes [37]. Second, HYLS1 and PMM2 are more than 950 kb away from any GWAS hits of schizophrenia (P<=5e-8), rendering their discoveries through standard GWAS or integrative strategies difficult. Our approach helps address this challenge by first identifying trait-associated HC ELEs, and then linking them to genes that are far away from GWAS signals via long-range enhancer-gene connections. As high- quality enhancer-gene maps are becoming available [37, 74], enhancer-centric approaches like ours will prioritize effector genes for complex traits beyond GWAS.*"

*1*

2) The nomenclature and organisation of the manuscript could be substantially improved for clarity and readability. The use of 'active enhancer-like elements' should be clarified. It is unclear if or how promoter regions were distinguished from enhancers as both types of regulatory elements would exhibit H3K27ac and open chromatin. For example, the concept of promoter H3K27ac signal is obviated on page 3 line 89. Given that promoter and enhancer regions can have substantially different levels of sequence conservation, the authors should clarify to what extent their chosen nomenclature is effectively enriching for promoter regions in some of the conservation categories used in the manuscript.

We thank Reviewer #1 for this insightful comment. To address this comment in full, we have provided additional clarifications and results, as detailed below.

Our use of 'enhancer-like elements' (ELEs) is inspired by the ENCODE Phase 3 flagship publication (PMID: 32728249), which is also cited as the **Ref. 9** in our original manuscript (**Page 30, Lines 874-875**).

As correctly pointed out by Reviewer #1, our approach to the identification of 'enhancer-like elements' (ELEs) will capture promoters with potential enhancer functions. In the revised manuscript, we have made this point clear when defining ELEs (**Page 3, Lines 97-100**), and we have cited the relevant publication (PMID: 28581502; **Ref. 31, Page 31, Lines 926-927**) to support this point.

Following Reviewer #1's suggestion, we have quantified the enhancer-like promoter regions in each of the four omnibus ELE categories created in the present study (NC: without considering any sequence conservation; LC, MC, HC: low, moderate, high levels of sequence conservation between the human and mouse genomes). Specifically, we have overlapped each omnibus ELE with the human candidate cis-regulatory elements (cCREs) from ENCODE Phase 3 (PMID: 32728249) and counted the ELE-cCRE pairs containing promoter-like signatures identified in ENCODE. Below are the numbers and percentages of such ELE-cCRE pairs for each of the four omnibus ELE categories.

| Omnibus ELE category | NC | LC | MC | HC |
| --- | --- | --- | --- | --- |
| Number of ELE-cCRE pairs containing promoter-like signatures | 39744 | 37600 | 35277 | 21902 |
| Percentage in all ELE-cCRE pairs | 4.323755% | 4.732518% | 5.384905% | 8.814711% |

From this new analysis, we can see that only a small fraction (4-9%) of the omnibus ELE-cCRE pairs overlap with promoter-like regions identified in ENCODE Phase 3 (PMID: 32728249). In addition, we find that HC ELEs tend to capture more enhancer-like promoters than NC ELEs (HC: 8.8% versus NC: 4.3%), which is consistent with previous findings that promoters are more conserved across species than enhancers (PMID: 25409824; PMID: 25635462). We have reported this new analysis in the revised manuscript (**Page 5, Lines 133-137**), as well as the supporting publications (**Ref. 25, Page 30, Lines 910-911**; **Ref. 30, Page 31, Lines 924-925**).

In response to this comment, we have made the following additions to our revised manuscript.

- **Page 3, Lines 97-100**: "*Since biochemical activity is not necessarily a definitive proof of enhancer function [1, 6] and some promoters may display enhancer activity [31], we cautiously termed these regions marked by H3K27ac and chromatin accessibility signals 'enhancer-like elements' (ELEs).*"
- **Page 5, Lines 133-137**: "*Around 4-9% of the omnibus ELE-cCRE pairs contain promoter-like signatures, likely capturing promoters with enhancer functions [31]. HC ELEs tend to capture more enhancer-like promoters than NC ELEs (HC: 8.8%; NC: 4.3%), recapitulating previous findings that promoters are more conserved across species than enhancers [25, 30].*"
- **Page 31, Lines 924-925**: "*[30] Yue, F. et al. A comparative encyclopedia of DNA elements in the mouse genome. Nature 515, 355–364 (2014).*"
- **Page 31, Lines 926-927**: "*[31] Dao, L. T. M. et al. Genome-wide characterization of mammalian promoters with distal enhancer functions. Nat. Genet. 49, 1073–1081 (2017).*"

After introducing the necessary clarifications and citing the relevant publications, we have clarified the use of 'enhancer-like elements' (ELEs) and addressed the enhancer-like promoters in our revised manuscript.

Further to this, a little more caution should be exercised around the classification of the regulatory elements as active (particularly as no functional validation is included in the manuscript). Line 3, 91 to 93: there is a contradiction in the statement that "biochemical activities are not necessarily definite proof of function", but within same sentence the authors class the putative elements that they identify as "active enhancer-like elements".

We agree with Reviewer #1 that "caution should be exercised" regarding "the classification of the regulatory elements as active", and we thank Reviewer #1 for this valuable comment. To address this comment, we have replaced the original term "active enhancer-like element" (AELE) with a new term "enhancer-like element" (ELE) throughout the revised manuscript.

More generally, the manuscript text and organisation should be improved for clarity and readability:

- Many of the figures and figure legends should be revised for consistency and clarity (for instance, see Figure 1i axis labels and the description in the figure legend).

We thank Reviewer #1 for this comment. During this round of revision, we have carefully checked each figure and its legend to ensure "consistency and clarity", as Reviewer #1 requested. Specifically, we have revised the axis labels and the legend of **Fig. 1i** as follows.

**Page 4, Fig. 1i Legend**: "***i*** *Each point denotes a pair of an ELE-based SNP annotation and one of the 96 known SNP annotations (Table S4), with the y and x-axis indicating correlations between the known SNP annotation and the SNP annotation for NC and HC ELEs, respectively. For* ***h****-****i****, the correlation of two binary annotations is quantified by Cramer's V (from 0 to 1; V=0: no correlation; V=1: complete correlation). The correlation of a binary and a quantitative annotation is quantified by Pearson's R (from -1 to 1; R=0: no correlation; |R|=1: complete correlation).*"

- Some sections of the manuscript text are difficult to follow with jumbled terminology ("functional annotations", "SNP annotations", "existing annotations"). The manuscript would benefit from some revision in this area, with use of clear definitions in the main text and consistent terminology.

We thank Reviewer #1 for this useful suggestion. We have revised the main text as follows.

- At its first mention in the revised manuscript, we have clearly defined the term "*SNP annotations*" as "*the annotations of common SNPs*" (**Page 6, Line 167**).
- After the definition, we have consistently used the term "*SNP annotations*" throughout the revised manuscript, and terms like "functional annotations" and "existing annotations" are no longer used in the main text.
- For brevity, we occasionally refer to "*SNP annotations*" as "annotations" and we believe that the readers can comprehend this shortened term from the context.

These wording changes ensure the use of "clear definition" and "consistent terminology" in our revised manuscript, as Reviewer #1 suggested.

3) The discussion of enhancer and promoter regions selected based on epigenomic conservation of H3K27ac signals (page 11, line 300-316) is useful to include in the manuscript, but we found the results presented here are poorly described (as is their contextualisation in the Discussion). The analysis presented by the authors seemingly compares regulatory regions identified on conservation of chromatin accessibility and H3K27ac signals in human and mouse (which the authors term "H3K27ac-conserved AELEs" and compared them with regions of high sequence conservation and H3K27ac/chromatin accessibility signals in human ("HC AELEs"). This analysis strategy is biased towards the authors approach and interpretation, in that comparison of high conservation AELEs are compared to "H3K27ac-conserved AELEs" is incomplete. AELEs defined by the authors are heterogeneous in their sequence conservation levels (Figures 1 and 2), as are "H3K27ac-conserved AELEs". A more nuanced analysis should also compare "like-for-like" subsets of AELEs and H3K27ac-conserved AELEs matched for their level of sequence conservation.

We greatly appreciate that Reviewer #1 finds the comparison of HC ELEs based on the mouse genome and mouse H3K27ac profile "useful to include in the manuscript". We also fully agree with Reviewer #1 that such a comparison needs to be conducted in a "like-for-like" manner such that the two approaches are "matched for their levels of sequence conservation".

Indeed, we have already included "a sequence conservation filter" in our original analysis to ensure the same level of sequence conservation for the "like-for-like" comparison as requested by Reviewer #1. Specifically, we wrote in the Methods of our original manuscript (**Page 23, Lines 638-639**) that "we first mapped H3K27ac peaks from the mouse to human genome using liftOver with the default setting and 'minMatch' being 0.9", which was the same sequence conservation level used in the creation of HC ELEs based on the mouse genome (**Page 5, Lines 113-116**; **Page 22, Lines 620-624**). In addition, the liftOver arrow in the original **Fig. S4 Panel a** indicates the existence of "a sequence conservation filter" in our original analysis.

To address this comment and improve clarity of our manuscript, we have introduced the following revisions to the main text and Fig. S4.

- **Page 12, Lines 325-327**: "*For each context, we created two sets of HC ELEs based on the same level of sequence conservation (minMatch=0.9) with the mouse genome (Fig. 1a) and mouse H3K27ac peaks (Fig. S4a; Methods), respectively.*"
- **Page 23, Lines 633-637**: "*To benchmark our primary approach (Fig. 1a), we developed an alternative method (Fig. S4a) to identify conserved ELEs in the human genome that*

*(1) displayed H3K27ac signals in both humans and mice for the same context and (2) reached the same level of human-mouse sequence conservation as our primary approach. We termed this alternative 'H3K27ac-conserved ELEs'.*"

- **Supplemental Information Page 10, Fig. S4a Legend**: "*To achieve a fair comparison, HC ELEs based on the mouse genome and mouse H3K27ac profile use the same level of sequence conservation (minMatch=0.9).*"
- **Supplemental Information Page 10, Fig. S4a**: We have added a new text annotation, "minMatch=0.9", near the liftOver arrow to highlight that the same level of sequence conservation was used in our comparison. Please see below.

With these revisions, we anticipate the readers to comprehend the important message that HC ELEs and H3K27ac-conserved ELEs are "matched for their levels of sequence conservation".

The discussion of the "H3K27ac conservation" approach is similarly imprecise in the Discussion section (lines 510-519). Such approaches, as cited by the authors, were often not designed as a post-GWAS annotation approach and have substantial value in answering a number of questions unrelated to the manuscript's. Therefore their discussion could be more balanced, and acknowledge the wider context in which many of these studies were undertaken.

We thank Reviewer #1 for this constructive comment. We agree with Reviewer #1 that we need to make our discussion here "more balanced" by (1) "acknowledging the wider context in which" the previous studies were conceived and (2) articulating the specific context in which our studyshows strengths. Following Reviewer #1's insightful suggestions, we have revised the following text in the Discussion.

**Page 21, Lines 558-560**: "*While the idea of combining evolutionary and biochemical data has proven broadly useful [14-17, 25], our study demonstrates several key strengths of implementing this idea specifically for multi-omics integrative analysis in GWAS.*"

Moreover, the authors could consider benchmarking their approach against existing methods of post-GWAS prioritisation (for instance integrating epigenomic and gene expression data without the use of sequence conservation).

We thank Reviewer #1 for this comment. We find the suggested benchmarking analysis broadly useful for the post-GWAS field. However, adding this analysis here is beyond the scope of our manuscript. Below is our detailed reasoning.

The present manuscript is firmly based on the comparison of:

- NC ELEs, which display high levels of enhancer-like biochemical activity;
- HC ELEs, which display high levels of both enhancer-like biochemical activity and human-mouse sequence conservation.

Through this comparison, the key message of this manuscript is that combining human epigenomic data with human-mouse sequence conservation (HC ELEs) can produce more informative SNP annotations for GWAS downstream analysis than those based on epigenomic data alone (NC ELEs). This key message is delivered by three in-depth post-GWAS applications: inferring SNP heritability enrichments (**Fig.s 2-3**), fine-mapping causal variants (**Fig. 4**), and identifying trait-associated regulatory elements (**Fig.s 5-6**).

While Reviewer #1 correctly points out other useful post-GWAS strategies such as integration of epigenomic and transcriptomic data, including these strategies here will inevitably complicate the comparison by introducing additional variables (e.g., sequence conservation versus transcription) and therefore blur the key message of this work. Hence, we respectfully opt not to include the suggested benchmarking in order to ensure the clarity and readability of this manuscript, which are advocated by Reviewer #1 in other comments.

We agree with Reviewer #1 that other strategies (e.g., integration of epigenomic and transcriptomic data) are useful in the broader context of post-GWAS analysis. As such, we have acknowledged these approaches in the Discussion of the revised manuscript (**Page 22, Line 595**), highlighting them as an important direction for future work beyond this manuscript.

**Minor comments**:

Page 21, line 560-562; The description for 'NC' categorisation could be more explicit to remove any disambiguation as "to identify AELEs without sequence conservation…" could imply conservation was not considered in the approach, i.e. all putative elements are included. However, "NC" and no conservation could also indicate all elements where sequence was not mapped between humans and mouse (i.e. "human-specific" in this two-way comparison).

We greatly appreciate this comment, and we fully agree with Reviewer #1 on the importance of explicitly defining 'NC' in the manuscript "to remove any disambiguation". In this work, 'NC ELEs' indicate all enhancer-like elements identified from epigenomic data alone, without considering any sequence conservation between species.

In response to this comment, we have added the following text in the revised manuscript.

- **Pages 3-5, Lines 109-111**: "*First, we generated all ELEs for each context without considering sequence conservation in the mouse genome, denoted as NC ELEs.*"
- **Page 22, Lines 617-620**: "*To identify NC ELEs without considering sequence conservation between the human and mouse genomes for each of the 106 contexts, we intersected H3K27ac and accessible chromatin peaks in this context using BEDTools*

*[87] (version 2.27.1).*"

These clarifications are expected to help the readers understand the definition of 'NC ELEs'.

Page 4, figure 1: The readability of this figure could be improved. For example, the relatively small palette of colours is carried through the figure without necessarily retaining context, for example panel 1a yellow denoting H3K27ac vs the rest of the figure where yellow is NC. Fig 1e label could be interpreted as DNase-CTCF and Fig 1h font is very small.

We thank Reviewer #1 for all the suggestions to improve the readability of Fig. 1. In response to this comment, we have revised **Fig. 1** (**Page 4**) as follows.

In the revised **Fig. 1a**, we have used a new color palette that differs from the palette for other panels, as shown below. This change will help avoid the confusion mentioned by Reviewer #1.

In the revised **Fig. 1e**, we have adjusted the x-axis labels to prevent potential misinterpretation.

In addition, we have added the description of "DNH3" in the revised **Fig. 1e Legend**: "*DNH3: signature marked by DNase and H3K4me3.*"

In the revised **Fig. 1h**, we have increased the font size through abbreviations, as shown below.

In addition, we have defined all the abbreviations in the revised **Fig. 1h Legend**: "*BN: bone. CB: cancer (blood). CO: cancer (other). CV: cardiovascular. CN: connective. EN: endocrine. EP: epithelial. GI: gastrointestinal. IM: immune. MS: muscle. NR: neural. OM: omnibus. OT: other tissues consisting of kidney, liver and lung. RP: reproductive. SK: skin. SC: stem cell.*"

Page 19 line 498 spelling error

We thank Reviewer #1 for this comment. We have fixed the typo and used the correct spelling "Fourth" (**Page 21, Line 555**).

Page 20 line 525 spelling error

We thank Reviewer #1 for this comment. We have fixed the typo and used the correct spelling "phylogenetic" (**Page 21, Line 582**).

# Reviewer #2 Comments

**Reviewer #2**: The manuscript by Zhu and colleagues studied how conservation of regulatory elements may be used to refine our understanding of genetics of complex traits. It shows that highly conserved elements (AELEs) are more enriched with heritability of traits, and with fine- mapped variants than general AELEs. This finding motivated the authors to develop a Bayesian statistical method to integrate AELE annotations with GWAS summary statistics to identify AELEs contributing to genetics of complex traits. The results from applying this approach to BMI and Schizopheria (SCZ) were discussed in details, with some novel genes highlighted.

Overall, this is a well-written paper. It provided systemtic evidence that HC regulatory sequences are important sources of information for human genetic studies. Furthermore, it provided a statistical method for researchers to leverage such information. Additionally, the results in the paper, including the novel risk genes of BMI and SCZ, would be good resources to the community. As such, I think the paper is of good interest to the broad audience of Genome Biology. Below are my specific comments:

We thank Reviewer #3 for the accurate summary and positive feedback on our manuscript. We are also very grateful for the insightful and constructive comments provided. We have addressed all the comments in full and revised the manuscript accordingly, as detailed below.

**Major comments**

1) The authors extended their earlier model, RSS-Net, in this paper. The details of the new method, however, are relatively sparse. There are few details about how the inference works, and no results to assess the method (e.g. simulation studies that often accompany new methods). The new method in fact differs quite a bit from the original RSS-Net. It was designed to solve a different biological problem, and the two differ in techincal aspects. In the original RSS-Net, causal variants have the same prior effect size, while in the new model, the prior effect sizes of variants depend on annotations. Given these differences, more details/justification of the new method may be necessary.

We agree that the original RSS-NET model (PMID: 33990562) and the new RSS-NET extension in the present manuscript differ in several aspects, as Reviewer #3 correctly pointed out. Despite their differences, the two RSS-NET models are closely related for two reasons. First, both models are built on the same multiple-SNP regression likelihood for GWAS summary statistics (PMID: 29399241). Second, through straightforward algebra, the prior used in the new RSS- NET extension can be shown as a special case of the prior used in the original RSS-NET model; see the new **Note S1** for derivation (**Supplemental Information Page 1**). Owning to the close connection of the two RSS-NET models, we used the extensive simulation results from the original RSS-NET publication (49 unique simulation scenarios, 200 simulated datasets per scenario; PMID: 33990562) to support the development of new RSS-NET extension, without conducting new simulation studies in the initial version of this manuscript.

We appreciate Reviewer #3's request for conducting new simulation studies that accompany the new RSS-NET extension. In the revised manuscript, we have reported results of 8 unique simulation scenarios that were specifically designed to evaluate the RSS-NET extension (see the next paragraph for details). Overall, the new simulation results confirm that the new RSS-NET method produces accurate estimation and valid inference, which is consistent with our previously published simulation results (PMID: 33990562).

In response to this comment, we have made the following additions to our revised manuscript.

- **Page 14, Lines 382-384**: "*After validating this RSS-NET extension through simulations (Note S2; Fig.s S5-8), we applied it to analyze the omnibus ELEs in the GWAS of BMI*

*[39] (Fig. 5) and schizophrenia [40] (Fig. 6).*"

- **Page 27, Lines 756-776**: "*We evaluated the RSS-NET extension through a large array of simulations (Note S2). To reduce the computation, we performed simulations on 348965 genome-wide common SNPs [98], with 19335 SNPs annotated by HC omnibus ELEs*

*(*𝑎_"_ = *1). To mimic the genetic architectures of various complex traits, we specified 8 simulation scenarios with varying proportions of (1) trait-associated SNPs and (2) phenotypic variation explained by all SNPs. For each scenario, we simulated 200 'positive' datasets where SNP effects (*𝛽"*) were simulated from priors (8)-(9) with the presence of enrichment parameters (*𝜃 > *0 or* 𝜎*^2^* > *0), as well as 200 'negative' datasets where SNP effects (*𝛽"*) were simulated from priors (8)-(9) without any enrichment (*𝜃 = *0 and* 𝜎*^2^* = *0). To ensure a fair comparison in each scenario, we matched the positive and negative datasets by the proportions of (1) trait-associated SNPs and (2) phenotypic variation explained by all SNPs. We combined the simulated SNP effects with the genotypes of 348965 genome-wide SNPs from 1458 individuals [98] to simulate phenotypes using an additive multiple-SNP model with Gaussian noise as previously described [38]. We performed the standard single-SNP analysis of simulated individual- level datasets to generate GWAS summary statistics, on which we compared RSS-NET results with the ground truth of each simulation scenario. The simulation results show that the RSS-NET extension produces accurate posterior estimation for model parameters (Fig. S5), as well as valid inference of enrichments (Fig. S6) and associations (Fig.s S7- S8).*"

- **Supplemental Information Page 1, Note S1**: Connection between the original RSS- NET and the present RSS-NET extension, including the derivation that shows the prior distribution used in the RSS-NET extension as a special case of the prior used in the original RSS-NET model.
- **Supplemental Information Pages 2-3, Note S2**: Details of the simulations to assess the RSS-NET extension.
- **Supplemental Information Page 11, Fig. S5**: Posterior estimation of enrichment parameters {𝜃, 𝜎} in the RSS-NET extension across 4 simulation scenarios.
- **Supplemental Information Page 12, Fig. S6**: Trade-off between false and true enrichments identified by the RSS-NET extension across 8 simulation scenarios.
- **Supplemental Information Page 13, Fig. S7**: Trade-off between false and true associations at the level of HC ELEs identified by the RSS-NET extension across 8 simulation scenarios.
- **Supplemental Information Page 14, Fig. S8**: False positive and discovery rates for 8 simulation scenarios across a wide range of 𝑃^!^ thresholds (0.45-0.95).

*1*

Altogether, these new additions are expected to provide the readers with sufficient details and justifications for the new RSS-NET extension described in this manuscript.

One particular concern is how to interpret P1, the metric the authors used to assess relevance of AELEs to the phenotypes. Given the sparse prior used in the model, it seems that the method is trying to perform fine-mapping, with a prior that favors HC AELEs. If this is the case, then P1 can be intepreted as as the probability that an AELE contains one or more causal variant. But this point is never explicitly mentioned. If this is indeed the goal here, then as in any fine- mapping analysis, it would be important to know if P1 is calibrated. Ex. at P1 > 0.9, are the proportion of false AELEs (those containing no causal variants) below 10%? This can be assessed through simulation studies.

We thank Reviewer #3 for this valuable comment concerning the calibration of our new method to identify trait-associated HC ELEs. We have performed the additional analysis as requested by Reviewer #3 and revised the manuscript accordingly. Below are our detailed responses.

We provided the following interpretations of 𝑃*_1_* in the original manuscript, which are consistent with Reviewer #3's interpretation above.

- **Page 15, Lines 390-392**: "*To quantify the genetic association between an ELE and a trait, we computed a posterior probability for each ELE that at least one SNP in this element is associated with the trait (*𝑃*_1_; Methods)*";
- **Page 26, Lines 746-747**: "*To identify if a HC ELE is associated with a trait, we used* 𝑃*_1_, the posterior probability that at least one SNP in the HC ELE is trait-associated*".

While Reviewer #3's interpretation of 𝑃*_1_* is essentially correct, we cautiously avoid labeling SNPs as "causal" and thus we opt to use the term "trait-associated" instead. In addition, we intentionally avoid using the term "fine-mapping" in this part of the manuscript. This is because another section of the manuscript presents results based on the standard fine-mapping analysis of likely causal SNPs (**Pages 12-14, Lines 338-377 & Fig. 4**; **Page 25, Lines 713-719**). Framing the methods and results related to 𝑃*_1_* as another fine-mapping analysis could lead to confusion.

We agree with Reviewer #3 on the importance of assessing the calibration in our analysis through simulation studies. Following Reviewer #3's suggestion above, we have assessed the calibration of our approach via the following two quantities in each of the 8 simulation scenarios:

- 'False positive rate', defined as the proportion of HC ELEs that do not contain any causal SNP but reach a given significance threshold of 𝑃^!^ (henceforth 'false positives') among all the HC ELEs that do not contain any causal SNP;

*1*

- 'False discovery rate', defined as the proportion of false positives among all the HC ELEs with posterior trait-associated probability under the enrichment prior for HC ELEs reaching a given threshold (e.g., 𝑃^!^ ≥0.9).

*1*

For each of the 8 simulation scenarios (**Note S2**), we aggregate the results of all 200 simulated datasets to compute the false positive and discovery rates. In the revised manuscript, we have visualized these results as the new **Fig. S8**. For the convenience of the reviewers, we include the **Fig. S8 Panel b** below to illustrate the new calibration analysis. The complete **Fig. S8** can be found in the **Supplemental Information Page 14**.

To answer the specific question raised by Reviewer #3 above, we show the false positive and discovery rates at 𝑃^!^ ≥ 0.9 across 8 simulation scenarios in the following table.

*1*

| Simulation scenario | False positive rate | False discovery rate |
| --- | --- | --- |
| PVE=0.6, 𝜃*0*=-4, 𝜃=2, 𝜎*0*=1, 𝜎=0 | 6.220132e-05 | 0.09899418 |
| PVE=0.6, 𝜃*0*=-4, 𝜃=2, 𝜎*0*=1, 𝜎=2 | 7.850006e-05 | 0.08912387 |
| PVE=0.6, 𝜃*0*=-2, 𝜃=2, 𝜎*0*=1, 𝜎=0 | 3.473513e-05 | 0.02177943 |
| PVE=0.6, 𝜃*0*=-2, 𝜃=2, 𝜎*0*=1, 𝜎=2 | 3.673055e-04 | 0.04032454 |
| PVE=0.2, 𝜃*0*=-4, 𝜃=2, 𝜎*0*=1, 𝜎=0 | 3.326274e-06 | 0.10101010 |
| PVE=0.2, 𝜃*0*=-4, 𝜃=2, 𝜎*0*=1, 𝜎=2 | 3.326274e-06 | 0.07751938 |
| PVE=0.2, 𝜃*0*=-2, 𝜃=2, 𝜎*0*=1, 𝜎=0 | 3.443951e-04 | 0.06541269 |
| PVE=0.2, 𝜃*0*=-2, 𝜃=2, 𝜎*0*=1, 𝜎=2 | 7.589995e-04 | 0.06783803 |

Across the 8 simulation scenarios, the false discovery rates at 𝑃^!^ ≥ 0.9 are all below or close to 0.1, confirming that the posterior trait-associated probabilities under the enrichment prior for HC ELEs (𝑃^!^) produced by the RSS-NET extension are well calibrated.

*1*

*1*

In response to this comment, we have made the following additions to our revised manuscript.

- **Page 15, Lines 395-398**: "*Here we used a significant association cutoff of* 𝑃^!^ ≥ *0.9, which yielded false positive rates less than 7.6e-04 and false discovery rates less than 0.1 across all simulation scenarios (Fig. S8).*"

*1*

- **Supplemental Information Page 14, Fig. S8**: False positive and discovery rates for 8 simulation scenarios across a wide range of 𝑃^!^ thresholds (0.45-0.95).

*1*

These new results demonstrate that our approach based on 𝑃^!^ is well calibrated.

*1*

2) Related to this point is the discovery of many HC AELEs for BMI and SCZ. The results of SCZ are particularly striking: the paper reported 173 HC AELEs at P1 > 0.9 using HC AELEs as the prior, compared to just 19 using a background (uniform) prior. The difference appears to be somewhat too large. For example, in the PolyFun paper [33199916], the increase of the number of fine-mapped variants by using functional prior is much more modest, about 30-50%. This raises the concern of calibration as pointed above. What explains the large difference of power from using HC AELE prior? What are the values of the hyperparameters learned from data? It would be useful to show some example(s) where the prior makes a difference. This would give the readers some idea of how the method finds new AELEs that would have been missed.

We greatly appreciate this insightful comment provided by Reviewer #3. Below are our point- by-point responses, along with detailed revisions to address this comment in full.

We have carefully reviewed the PolyFun paper (PMID: 33199916). However, we believe that the results from the PolyFun paper may not be directly comparable to those from our manuscript due to fundamental differences in focus. Specifically, the PolyFun paper is centered on fine-mapping of causal SNPs, while our manuscript primarily focuses on the identification of trait-associated HC ELEs. In essence, PolyFun generates the posterior causal probability of a SNP within a 3-Mb locus for a given trait, whereas our approach calculates the posterior probability that at least one SNP in a HC ELE is associated with a trait. Given that the two studies assess evidence for genetic associations at different levels (PolyFun: each SNP; ours: each HC ELE), we find it challenging to draw direct comparisons between our findings and those from the PolyFun paper.

As detailed in our responses to the previous comment, we have conducted extensive simulations to confirm the calibration of the posterior trait-associated probabilities under the enrichment prior for HC ELEs (𝑃^!^) produced by the RSS-NET extension (**Fig. S8**). Therefore, the additional genetic associations identified for BMI and schizophrenia under the enrichment prior for HC ELEs are unlikely to be caused by any calibration issues.

*1*

The posterior estimates of hyper-parameters {𝜃*_0_*, 𝜃, 𝜎*^2^*, 𝜎*^2^*} help explain the larger number of genetic associations for BMI and schizophrenia identified under the enrichment prior for HC ELEs, as compared to the baseline prior. On the same GWAS, the RSS-NET extension gives similar estimates for the baseline parameters {𝜃*_0_*, 𝜎*^2^*} under both the baseline and enrichment models, and it produces positive estimates for the enrichment parameters {𝜃, 𝜎*^2^*} if HC ELEs are identified as enriched for genetic associations with BMI (**Fig. 5a**) and schizophrenia (**Fig. 6a**).

*0*

*0*

The positive estimate of 𝜃 increases 𝜋" = (*1* + *10*^#(%^*0*&'!%))^#^*^1^*, the prior probability of

association for SNPs in HC ELEs, which in turn increases the posterior probability of association

for these SNPs. The positive estimate of 𝜎*^2^* increases 𝜎*^2^* = 𝜎*^2^* + 𝑎_"_𝜎*^2^*, the prior effect size for

"	*0*

trait-associated SNPs in HC ELEs, which in turn increases the posterior effect size for these SNPs. Collectively, the positive enrichment parameter estimates enhance the evidence for associations between SNPs in HC ELEs and the GWAS trait, leading to the identification of more trait-associated elements under the enrichment model for HC ELEs. Below are the posterior means of {𝜃*_0_*, 𝜃, 𝜎*^2^*, 𝜎*^2^*} estimated in each GWAS under each model.

*0*

| GWAS | BMI | | Schizophrenia | |
| --- | --- | --- | --- | --- |
| Model | Baseline | HC ELE | Baseline | HC ELE |
| E(𝜃*_0_* \| Data) | -1.999552 | -2.014042 | -1.988379 | -1.990281 |
| E(𝜃 \| Data) | NA | 0.9323156 | NA | 0.7485804 |
| E(𝜎*^2^* \| Data)  *0* | 1.259058e-05 | 1.094558e-05 | 1.603915e-04 | 1.186280e-04 |
| E(𝜎*^2^* \| Data) | NA | 1.112187e-06 | NA | 6.868873e-05 |

We have reported theses posterior estimates of {𝜃*_0_*, 𝜃, 𝜎*^2^*, 𝜎*^2^*}, along with the reasoning above, as a new **Note S3** in the **Supplemental Information Pages 3-4**.

*0*

We agree with Reviewer #3 on the importance of "showing examples where the prior makes a difference". Indeed, we provided such examples as **Fig.s 5c-d** and **6c-d** in our original manuscript: HC ELEs that contain instances of certain motifs display higher posterior trait- associated probabilities under the enrichment prior for HC ELEs (𝑃^!^) compared to those under the enrichment prior for NC ELEs (𝑃^)^), for both BMI (**Fig.s 5c-d**) and schizophrenia (**Fig.s 6c- d**). To address Reviewer #3's comment here, we have expanded these examples in a new **Fig. S9** by adding two more comparisons:

*1*

*1*

- posterior trait-associated probabilities under the enrichment prior for HC ELEs (𝑃^!^)

*1*

versus those under the baseline prior without any enrichment (𝑃^*^);

*1*

- posterior trait-associated probabilities under the enrichment prior for NC ELEs (𝑃^)^)

*1*

versus those under the baseline prior without any enrichment (𝑃^*^).

*1*

For the convenience of the reviewers, we include the **Fig. S9 Panel a** below to illustrate the new comparison. The complete **Fig. S9** can be found in the **Supplemental Information Page 15**.

These new comparisons, together with the previous results, showcase that the enrichment prior for HC ELEs enhance the statistical evidence for genetic associations between HC ELEs and GWAS traits, thus supporting the conclusion in our original manuscript.

In response to this comment, we have made the following additions to our revised manuscript.

- **Page 15, Lines 407-413**: "*The enhanced evidence for genetic associations (measured by*

𝑃^!^*) is attributed to the enrichment-informed design of RSS-NET for prioritizing associations at HC ELEs. Specifically, once the enrichment of HC ELEs is identified for a trait, RSS-NET automatically increases the prior association probability and effect size for SNPs therein, which in turn increases the posterior association probability and effect size for these SNPs (Note S3).*"

*1*

- **Supplemental Information Pages 3-4, Note S3**: Prior specification and posterior estimation of hyper-parameters in the RSS-NET analysis of HC omnibus ELEs and GWAS of BMI and schizophrenia, as well as the reasoning behind how positive estimates for the enrichment parameters lead to increased evidence for associations between SNPs in HC ELEs and the GWAS trait.
- **Supplemental Information Page 15, Fig. S9**: Comparison of posterior trait-associated probabilities under the enrichment model for HC ELEs (𝑃^!^), the enrichment model for

*1*

NC ELEs (𝑃^)^), and the baseline model without any enrichment (𝑃^*^), for the same HC

*1	1*

ELE in the same GWAS.

With these new additions, we anticipate that readers will have sufficient information to understand how our new method could potentially identify additional trait-associated regulatory elements that might otherwise be overlooked.

3) The authors reported some novel candidate genes for BMI and SCZ. Again, it is unclear how the new method found these genes, while earlier efforts miss them. Is this due to the better power of "fine-mapping" HC AELEs? Or are these genes far from the GWAS lead variants, so that they were missed previously but now were discovered because of the use of distal interaction data? It would be helpful to show one or two figure to provide the genetic evidence supporting the HC AELEs (GWAS p-values, P1), as well as genomic data linking the AELEs with the genes.

We thank Reviewer #3 for this constructive comment. In response to this comment, we have added new text and figures to the revised manuscript as suggested by Reviewer #3.

First, we have added the following paragraph in the Discussion section of the revised manuscript, which aims to help the readers better understand why "the new method found these genes, while earlier efforts missed them", as Reviewer #3 inquired.

**Page 20, Lines 522-544**: "*Integrating HC ELEs with GWAS and gene regulatory networks further helps pinpoint previously undescribed but functionally relevant genes for BMI (e.g., CDK5, HSD11B1) and schizophrenia (e.g., HYLS1, PMM2). Despite convergent evidence supporting their roles in the biology of BMI and schizophrenia, these genes prioritized by our approach were not identified by the same GWAS data used in our analysis, nor by the updated GWAS with much larger sample sizes, nor by the multi-omics integrative analysis of these GWAS data at the time of this study [39, 40, 45, 72, 73]. Identifying these genes post-GWAS presents two challenges. First, although CDK5 and HSD11B1 are within the loci (+/-500 kb) of known GWAS hits of BMI (rs2907948, P=1.3e-13; rs12140373, P=7.7e-11), these loci contain many protein-coding genes (28 and 10, respectively), thus complicating the nomination of likely causal genes. Our approach helps address this challenge by shortlisting genes on the same regulatory circuit as HC ELEs that are strongly associated with the GWAS trait, based on the premise that enhancers affect a trait through their downstream genes [37]. Second, HYLS1 and PMM2 are more than 950 kb away from any GWAS hits of schizophrenia (P<=5e-8), rendering their discoveries through standard GWAS or integrative strategies difficult. Our approach helps address this challenge by first identifying trait-associated HC ELEs, and then linking them to genes that are far away from GWAS signals via long-range enhancer-gene connections. As high- quality enhancer-gene maps are becoming available [37, 74], enhancer-centric approaches like ours will prioritize effector genes for complex traits beyond GWAS.*"

Second, we have added **Fig.s S11-14** in the **Supplemental Information Pages 17-20** that show "the genetic evidence supporting the HC ELEs (GWAS p-values, P1), as well as genomic data linking the ELEs with the genes", as Reviewer #3 requested. For the convenience of the reviewers, we include the **Fig. S11** below to illustrate the new visualization.

This figure provides all the information requested by Reviewer #3, as detailed below.

- **Panel a**: Each dot represents a GWAS SNP in the given genomic region, with its GWAS P-value on the −log10 scale shown on the vertical axis and its chromosomal position shown on the horizontal axis. The purple horizontal dashed line indicates the genome- wide significance threshold (P=5e-8) on the −log10 scale.
- **Panel b**: Each triangle represents a HC ELE in the given genomic region, with its posterior trait-associated probability under the enrichment model for HC ELEs (𝑃^!^) shown on the vertical axis and its chromosomal position shown on the horizontal axis. The red horizontal dashed line indicates the significance threshold for posterior trait- associated probabilities (𝑃^!^=0.9).

*1*

*1*

- **Panel c**: Each triangle represents the same trait-associated HC ELE (𝑃^!^ ≥ *0.9*) identified by the RSS-NET extension in the given genomic region, and each bar represents a protein-coding gene. If the trait-associated HC ELE is linked to a gene according to a recently published gene regulatory network (PMID: 30545857), then they are connected by a curved edge. The edge color corresponds to the estimated edge weight in the gene regulatory network.

*1*

Across three panels, the green vertical solid line indicates the trait-associated HC ELE (𝑃^!^ ≥ *0.9*) identified by the RSS-NET extension, and the blue rectangular area indicates the prioritized effector gene that is (1) likely regulated by the trait-associated HC ELE and (2) supported by multiple lines of external evidence (**Fig. S15**).

*1*

**Minor comments**

- Definition of HC, MC and LC elements: are these classes disjoint? It seems that they are not, based on the numbers. But the readers have to infer that point. Given that these classes are really fundamental to the paper, it would be helpful to clarify this in the main text.

Reviewer #3 is correct that HC, MC and LC are not disjoint. We agree with Reviewer #3 that we need to make this point clear in the main text. In response to this comment, we have added the following clarifications in the revised manuscript.

- **Page 3, Lines 104-107**: "*Consequently, a conserved ELE became a smaller subset of the original ELE as the sequence conservation level increased, and an ELE might not have a conserved subset if the conservation level was set too high.*"
- **Page 5, Lines 113-116**: "*We then created three non-disjoint sets of conserved ELEs in the same context by setting the sequence conservation level as 0.1, 0.5 and 0.9, denoted as lowly (LC), moderately (MC) and highly conserved (HC) ELEs, respectively.*"
- **Pages 22-23, Lines 625-627**: "*Since each conserved ELE is a subset of the original ELE (Fig. 1a), the sets of NC, LC, MC and HC ELEs are not disjoint for each context.*"
- In the text about Fig. 1i (the comparison of HC AELE with other annotations): what does V mean? The figure legend says "correlation". It's better to use consistent terms here.

The *V* statistic denotes the Cramer's *V*, which ranges from 0 to 1 and measures the correlation between two binary annotations (*V=*0: no correlation; *V=*1: complete correlation). The interpretation of Cramer's *V* was provided in the Methods section of the original manuscript (**Page 23, Lines 654-656**), as well as the **Table S3 Legend** (**Supplemental Information Page 4**). Following the suggestion from Reviewer #3, we have revised the **Fig. 1i Legend** for better clarity and consistency as follows.

**Page 4, Fig. 1i Legend**: "*For* ***h****-****i****, the correlation of two binary annotations is quantified by Cramer's V (from 0 to 1; V=0: no correlation; V=1: complete correlation). The correlation of a binary and a quantitative annotation is quantified by Pearson's R (from -1 to 1; R=0: no correlation; |R|=1: complete correlation).*"

- The extended RSS-Net method in the paper seems quite similar to the bfGWAS method (PMID:28844487). It should be cited, and perhaps discussed.

We thank Reviewer #3 for this suggestion. We have cited the bfGWAS paper (PMID: 28844487) to support the prior specification for the RSS-NET extension (**Page 26, Lines 733 & 736**). The bfGWAS paper is now included as **Ref. 96** in the revised manuscript, as shown below.

**Page 34, Lines 1073-1076**: "*[96] Yang, J., Fritsche, L. G., Zhou, X., Abecasis, G. & International Age-Related Macular Degeneration Genomics Consortium. A scalable Bayesian method for integrating functional information in genome-wide association studies. Am. J. Hum. Genet. 101, 404-416 (2017).*"

Although its prior specifications are similar to those used in our RSS-NET extension, the bfGWAS approach requires the input of individual-level genotypes and phenotypes from a GWAS, and thus it cannot be utilized for the analysis in the present manuscript.

- Linking enhancers to genes: the authors cited a few studies, but there are few details of how this is done. Are these based on Hi-C data, or cis-eQTLs or other methods? What specific datasets are used? Ex. Supplementary Table(s) in the original studies. Such details need to be provided for the study to be reproducible.

We agree with Reviewer #3 on the importance of providing the method descriptions and data sources for each study that we used to create the enhancer-gene linkages, in order to ensure the reproducibility of the present study. We provide all the information requested by Reviewer #3 in the table shown on the following page.

| Study | Method summary | Data source |
| --- | --- | --- |
| Adult mouse brain | This study integrated snATAC-seq with scRNA-seq data to connect candidate  cis-regulatory elements (cCREs) to genes on the basis of both co-accessibility between cCREs and positive association with gene expression. | For each cell type, the BEDPE file of gene-cCRE connections is available at <http://catlas.org/catlas_downl> oads/mousebrain/conns/. |
| Fetal human brain | This study used Hi-C data to identify linkages of enhancers and genes. | The linkages are available as the "Table S5: enhancer-gene predictions" in the publication (https://doi.org/10.1186/s130 73-021-00980-1). These  linkages are also available at https://doi.org/10.6084/m9.fig share.16829164.v1. |
| Adult human brain | This study first combined Hi-C linkages, quantitative-trait loci (QTLs), transcription factor (TF) binding sites to build a reference network of gene regulation in brain, and then refined enhancer-gene connections by relating the activity of TFs to expression of target genes via elastic net regression. The network construction used both the repeat-masked TF binding site map (GRN1) and the complete map (GRN2). | INT-11 (GRN1):  [http://resource.psychencode.o](http://resource.psychencode.o/) rg/Datasets/Integrative/INT- 11_ElasticNet_Filtered_Cutof f_0.1_GRN_1.csv  INT-14 (GRN2):  [http://resource.psychencode.o](http://resource.psychencode.o/) rg/Datasets/Integrative/INT- 14_ElasticNet_Filtered_Cutof f_0.1_GRN_2.csv |

In response to this comment, we have incorporated all the requested information from the table above into the Methods (**Page 28, Lines 794-821**) and References sections (**Page 34, Lines 1092-1099**) of the revised manuscript. With these newly added method descriptions and data sources, we anticipate the readers to have the necessary information to reproduce the enhancer- gene linkage analysis in our manuscript.

- Fig. 5 legend say, "BMI associations of c". This looks confusing. One would usually put Panel number first, then text.

We thank Reviewer #3 for this useful comment. As suggested, we have put the panel numbers before the text in the **Fig. 5 Legend** of the revised manuscript (**Page 16**). In addition, we have revised the **Fig. 6 Legend** in the same way (**Page 19**).

**Second round of review**

**Reviewer 1**

Overall the authors have done a good job in addressing reviewer concerns, and the revised manuscript has been substantially improved as a result.

Nevertheless, we have some outstanding minor comments for the authors to consider:

- Page 3, lines 104-107: in our opinion, this clarification is somewhat obscure as written by the authors. We recommend this is reworked for clarity.

- Page 5, lines 133-137: Whilst the authors have sought to address enhancer-like promoters, they do not clarify how/if they negated the potential for inclusion of promoters acting as promoters within their enhancer-like element population - in other words, the assumption that "ELE-cCRE pairs containing promoter-like signatures likely capture promoters with enhancer functions" seems largely unwarranted. For example, in Figure 1 e, there are separate classifications for promoter-like element (PL) and DNase sensitive/ H3K4me3 (DNH3), the latter of which are hallmarks of promoters. This can be substantiated from the ENCODE publication cited (ref. 9). In addition, if the approach outlined in ref.9 is what the authors utilised in distinguishing the different regulatory elements classes, it would be useful to have more direct mention of criteria or indeed citation in context of methods, as the authors of the ENCODE paper used more criteria such as H3K4me3 signal and TSS distance to subset the types of regulatory elements. This information is lacking in the current manuscript, yet would aid reader comprehension.

Moreoever, the percentages of ELEs with "promoter-like" signatures seems relatively low. For completeness, and in addition to any ENCODE estimates based on biochemical signatures, it would be helpful to complement ELEs annotation with distance to annotated transcriptional start sites. What percentage of ELEs are proximal to annotated TSS?

- Page 22, lines 601-603: The authors could include mention and references to the recent Zoonomia consortium studies, which have included use of evolutionary conservation to annotate heritability of human traits and diseases.

**Reviewer 2**

With new simulations and results, the authors have significantly strengthened the paper. I still have some comments though:

(1) The RSS-Net method requires reference LD. What LD panel was used in the analysis of real data? It is known fine-mapping analysis is susceptible to false positive findings when the reference LD does not match the in-sample LD. Given that in-sample LD of large meta-analysis is often not available, what external LD reference panel is used is critical. In fact, even with the best LD reference panel, e.g. from a large number of samples matching ancestries, fine-mapping may still report false positives. In such cases, it would be important to filter out possible LD mismatches. This has been implemented in several recent papers, including DENTIST, SuSiE-RSS and CARMA. While RSS-Net is not explicitly doing fine-mapping, the underlying statistical model is very similar. Has such filtering be done in real data analysis?

To see why this is a concern, one can look at the example in Fig. S11 (page 22 of the letter). It seems that the method found multiple signals in this region. The highlighted ELE is not the top signal in the region. This could be real, but one can also imagine that the association of the ELE with the trait is actually due to LD with the top signal. But the LD reference panel may miss this LD, and report the ELE as a secondary signal.

(2) The scales of the parameters (prior effect variance) seem to be different in simulations (page 18 of the letter) and in real data (page 19). It would be good to report the results in the same scale.

**Authors’ response to reviewers**

We sincerely thank Reviewers #1 and #2 for their positive evaluations and constructive comments on our recently revised manuscript **GBIO-D-22-01305R1** entitled "*Genetic effects of sequence-conserved enhancer-like elements on human complex traits*". We have carefully considered all the new comments and revised our manuscript accordingly. Below, we provide the point-by-point responses to all comments. Reviewer comments are shown in blue Arial font, and our responses are shown in black. All display items, page numbers, and line numbers referenced below correspond to the newly revised manuscript. All changes in the revised manuscript to address the new comments are underlined in blue. All of these changes are also included here for the convenience of both reviewers and editors.

# Reviewer #1 Comments

**Reviewer #1**: Overall the authors have done a good job in addressing reviewer concerns, and the revised manuscript has been substantially improved as a result. Nevertheless, we have some outstanding minor comments for the authors to consider:

We thank Reviewer #1 for the positive feedback on our revision. We have addressed all the new comments from Reviewer #1 in full and revised the manuscript accordingly, as detailed below.

Page 3, lines 104-107: in our opinion, this clarification is somewhat obscure as written by the authors. We recommend this is reworked for clarity.

We thank Reviewer #1 for this constructive comment. To improve the clarity, we have revised this part of the main text as follows.

**Pages 4-5, Lines 179-187**: "*Under this definition, a higher level of sequence conservation would pose a more stringent threshold for a given ELE to be classified as conserved. Consequently, the collection of conserved ELEs became a smaller subset of all ELEs as the conservation level increased.*"

Page 5, lines 133-137: Whilst the authors have sought to address enhancer-like promoters, they do not clarify how/if they negated the potential for inclusion of promoters acting as promoters within their enhancer-like element population - in other words, the assumption that "ELE-cCRE pairs containing promoter-like signatures likely capture promoters with enhancer functions" seems largely unwarranted. For example, in Figure 1 e, there are separate classifications for

promoter-like element (PL) and DNase sensitive/ H3K4me3 (DNH3), the latter of which are hallmarks of promoters. This can be substantiated from the ENCODE publication cited (ref. 9).

We greatly appreciate this valuable comment from Reviewer #1. We agree that a small fraction of the identified ELEs may capture regulatory elements in close proximity to "promoters acting as promoters", in addition to "enhancer-like promoters". To address this comment and enhance clarity, we have revised the main text as follows.

**Page 6, Lines 234-251**: "*While the vast majority of ELEs map to promoter-distal regions that are more than 2 kb away from a transcriptional start site (TSS), a small fraction of ELEs may lie in close proximity to promoters. Specifically, approximately 1-3% of the omnibus ELEs fall within 200 bp of an annotated TSS, and roughly 4-9% of the omnibus ELE-cCRE pairs possess promoter-like signatures that are marked by H3K4me3 strength and TSS proximity (Methods).*

*These ELEs may denote TSS-proximal enhancers (Ref. 31), enhancer-like promoters (Ref. 32), or other functional elements around canonical promoters (Ref. 9). Compared to NC ELEs, HC ELEs tend to overlap with a TSS more frequently (3.1% versus 0.9%; Fig. S1e) and capture more promoter-like signatures (8.8% versus 4.3%; Fig. 1e). These observations recapitulate previous findings that promoters are more conserved across species than enhancers (Ref.s 25 and 30).*"

In addition, if the approach outlined in ref.9 is what the authors utilised in distinguishing the different regulatory elements classes, it would be useful to have more direct mention of criteria or indeed citation in context of methods, as the authors of the ENCODE paper used more criteria such as H3K4me3 signal and TSS distance to subset the types of regulatory elements. This information is lacking in the current manuscript, yet would aid reader comprehension.

In the present study, we compare the ELEs with the human cCREs cataloged in the ENCODE Phase 3 flagship publication (Ref. 9; PMID: 32728249) to assess and understand the biochemical relevance of ELEs. We agree with Reviewer #1 that a "more direct mention of" how ENCODE defined "different regulatory elements classes" can "be useful" and "aid reader comprehension". As such, we have added the following subsection entitled "*ENCODE cCRE classifications*" in the revised Methods section (**Pages 24-25, Lines 1092-1112**) and referenced this new Methods subsection in the revised Results section accordingly (**Page 5, Line 222; Page 6, Line 243**).

**Pages 24-25, Lines 1092-1112**: "*To assess their biochemical relevance, we compared ELEs with the human cCREs (version 3) cataloged in Phase 3 of the ENCODE Project (Ref. 9). The ENCODE cCREs were classified into the following five groups (Fig. 1e). Distal enhancer-like signature (dELS) elements are cCREs with high DNase and H3K27ac signals that are located more than 2 kb from the nearest TSS. Proximal enhancer-like signature (pELS) elements are cCREs with high DNase and H3K27ac but low H3K4me3 signals that are located within 2 kb of a TSS. Promoter-like signature (PLS) elements are cCREs with high DNase and H3K4me3*

*signals that are located within 200 bp of a TSS. Other high-DNase and high-H3K4me3 (DNH3) elements are cCREs with high DNase and H3K4me3 signals but low H3K27ac signals that do not reside within 200 bp of a TSS. CTCF-only elements are cCREs that possess high DNase and CTCF signals but low signals for H3K4me3 and H3K27ac.*"

Moreoever, the percentages of ELEs with "promoter-like" signatures seems relatively low. For completeness, and in addition to any ENCODE estimates based on biochemical signatures, it would be helpful to complement ELEs annotation with distance to annotated transcriptional start sites. What percentage of ELEs are proximal to annotated TSS?

We agree with Reviewer #1 that "it would be helpful to complement ELEs annotation with distance to annotated transcriptional start sites (TSSs)", and we thank Reviewer #1 for this insightful suggestion. As requested, we have presented the "percentages of ELEs proximal to annotated TSS" as a new **Fig. S1 Panel e** in the revised **Supplemental Information (Page 8)** and referenced this new figure in the revised Results section accordingly (**Page 6, Line 248**). We include this new figure below for the convenience of reviewers.

We have followed the ENCODE Phase 3 flagship publication (Ref. 9; PMID: 32728249) to perform this new analysis. Specifically, we first determine the center of each ELE, and then we categorize an ELE as (1) "TSS-overlapping" if its center is located within 200 bp of a TSS, (2) "TSS-proximal" if its center falls between 200 bp and 2 kb of a TSS, and (3) "TSS-distal" if the genomic distance from its center to the nearest TSS exceeds 2 kb. The annotated TSS list is obtained from UCSC RefGene hg19.

The percentages of omnibus ELE-cCRE pairs containing promoter-like signatures (NC: 4.32%, LC: 4.73%, MC: 5.38%, HC: 8.81%; Page 6, Line 239) align with the percentages of omnibus ELEs proximal to annotated TSS as shown above. Furthermore, the ENCODE Phase 3 flagship publication (Ref. 9; PMID: 32728249) reports that 3.7% of human cCREs are promoter-like elements, which is also consistent with our results.

Page 22, lines 601-603: The authors could include mention and references to the recent Zoonomia consortium studies, which have included use of evolutionary conservation to annotate heritability of human traits and diseases.

We thank Reviewer #1 for providing this timely and relevant reference, which was published 8 months subsequent to the initial submission of our manuscript. Upon reviewing the latest collection of 11 research articles that were published by the Zoonomia project in *Science* on April 28, 2023, we determine that the study most pertinent to Reviewer #1's comment should be Sullivan PF, et al. (2023). Leveraging base-pair mammalian constraint to understand genetic variation and human disease. *Science*, 380(6643), eabn2937 (PMID: 37104612). This study reports that "constrained positions are enriched for variants that explain common disease heritability more than other functional annotations."

Following the suggestion of Reviewer #1, we have cited this Zoonomia study in the Conclusions section of our revised manuscript (**Ref. 8, Page 22, Line 1006**). In addition, we have cited the same study in the Introduction section (**Ref. 8, Page 2, Line 77**).

Lastly, we would like to underscore the *significant conceptual difference* between this Zoonomia publication (PMID: 37104612) and our manuscript. The Zoonomia study compares genomes of 240 placental mammals to identify evolutionarily constrained regions in the human genome, and then illustrates the enrichment of SNP-heritability in constrained regions across 63 GWASs. Our study integrates human epigenomic data with human-mouse genome comparison to identify sequence-conserved enhancer-like elements (HC ELEs) in the human genome across 106 tissues and cell types, and then demonstrates the heritability enrichment in HC ELEs across 468 GWASs. In other words, this Zoonomia study exclusively employs evolutionary information whereas our study combines both evolutionary and biochemical information to detect functional elements in the human genome for post-GWAS analysis. Hence, our manuscript both complements and significantly departs from the Zoonomia publication (PMID: 37104612).

# Reviewer #2 Comments

**Reviewer #2**: With new simulations and results, the authors have significantly strengthened the paper. I still have some comments though:

We thank Reviewer #3 for the positive feedback on our revision. We have addressed all the new comments from Reviewer #3 in full and revised the manuscript accordingly, as detailed below.

(1) The RSS-Net method requires reference LD. What LD panel was used in the analysis of real data? It is known fine-mapping analysis is susceptible to false positive findings when the reference LD does not match the in-sample LD. Given that in-sample LD of large meta-analysis is often not available, what external LD reference panel is used is critical. In fact, even with the best LD reference panel, e.g. from a large number of samples matching ancestries, fine- mapping may still report false positives. In such cases, it would be important to filter out possible LD mismatches. This has been implemented in several recent papers, including DENTIST,

SuSiE-RSS and CARMA. While RSS-Net is not explicitly doing fine-mapping, the underlying statistical model is very similar. Has such filtering be done in real data analysis?

We agree with Reviewer #3 on the importance of careful quality control (QC) for LD matrices derived from external reference panels when analyzing GWAS summary statistics. Indeed, we diligently executed a series of QC procedures to ensure consistency between the GWAS summary statistics and the reference LD matrices in the present study while preparing the manuscript for initial submission on September 2, 2022. During this round of revision, we have further surveyed the latest literature (including the three papers suggested by Reviewer #3) and confirmed that our existing QC procedures can effectively tackle the known sources of possible LD mismatches in the analysis of GWAS summary statistics to date. The following table lists the major sources of inconsistencies between GWAS summary statistics and reference LD estimates, as well as our QC procedures implemented in this study to address these issues.

| **Source of potential GWAS-LD mismatches** | **QC procedure used in the present study** |
| --- | --- |
| Differences in population or ancestry between the sample to generate GWAS summary statistics and the sample to derive reference LD estimates. | Since the GWAS summary statistics of BMI (PMID: 30124842) and schizophrenia (PMID: 29483656) analyzed in this study were generated from European ancestry cohorts, we estimated LD using the haplotypes of unrelated individuals with European ancestry from Phase 3 of the 1000 Genomes Project (PMID: 26432245). |
| Errors in genotyping or imputation, as well as genetic variants with incorrectly specified effect alleles. | We used the HapMap3 SNPs (PMID: 20811451) as an approximation of the well- imputed genome-wide SNPs (PMID: 26414678), and we further excluded SNPs on sex chromosomes, SNPs with minor allele frequency less than 1%, and SNPs in the major histocompatibility complex region to avoid potential LD mismatches caused by poorly genotyped or imputed SNPs and incorrect allele encodings. |
| Inconsistent sample size coverage at different genetic variants when meta-analyzed cohorts are genotyped or imputed at variable density. | We excluded SNPs that were not analyzed in all individual cohorts in the GWAS meta- analysis, such as SNPs measured on custom arrays [e.g., Metabochip (PMID: 22876189) and Immunochip (PMID: 21345260)]. |
| 'Allele flip' in the sense that alleles in the GWAS summary statistics are encoded differently from those in the reference LD | For each SNP analyzed, we checked whether its alleles were consistently encoded in both the GWAS summary statistics and the |

| estimates. | reference LD estimates. We manually adjusted SNPs that used different allele encodings prior to any downstream analysis. |
| --- | --- |

Inspired by this helpful comment from Reviewer #3, we have incorporated the table above along with relevant references as a new **Note S4** in the revised **Supplemental Information (Page 4)**. With the addition of the new text and references, we anticipate that readers will have adequate information to comprehend (1) the LD reference panels that we used and (2) the QC procedures that we implemented to minimize potential LD mismatches in the present study.

To see why this is a concern, one can look at the example in Fig. S11 (page 22 of the letter). It seems that the method found multiple signals in this region. The highlighted ELE is not the top signal in the region. This could be real, but one can also imagine that the association of the ELE with the trait is actually due to LD with the top signal. But the LD reference panel may miss this LD, and report the ELE as a secondary signal.

To thoroughly address Reviewer #3's comment on the potential LD mismatches in our analyses of real data, we have conducted the following three diagnostics to verify the consistency between the GWAS summary statistics and the reference LD estimates for a given locus in this study.

- We estimate a regularization parameter 𝜆 ∈ [*0*,*1*] as 𝜆# = 𝑎𝑟𝑔𝑚𝑎𝑥𝜆𝑁(𝑧; *0*, (*1* − 𝜆)𝑅 +

𝜆𝐼), where 𝑧 is the vector of observed GWAS *Z* scores for all SNPs in the locus, 𝑅 is the LD matrix for these SNPs estimated from the reference panel, and 𝐼 is an identity matrix that matches the dimension of 𝑅. The estimated 𝜆# reflects the consistency between the GWAS summary statistics (𝑧) and the reference LD matrix (𝑅) -- if 𝑧 and 𝑅 are consistent with one another, 𝜆# will be close to zero. We use the 'estimate_s_rss' function from the R package susieR (Ref. 95) to estimate 𝜆.

- We compute a likelihood ratio (LR) statistic for each SNP to assess whether its alleles in the GWAS are encoded differently from the reference LD (a.k.a. "allele flip"), and we report the maximum LR value among SNPs with observed GWAS *Z* scores greater than two in magnitude (i.e., |𝑍| > *2*). If a SNP does not have an allele flip, the estimated LR will be less than one (i.e., 𝑙𝑜𝑔𝐿𝑅 < *0*). We use the 'kriging_rss' function from the R package susieR to calculate the LR statistic of SNP for detecting allele flips.
- We also use the 'kriging_rss' function to compute the expected *Z* score of each SNP conditional on all other SNPs in a given locus, and then compare it with the observed GWAS *Z* score of the same SNP. A large difference between the expected and observed *Z* scores of the same SNP indicates potential LD mismatches caused by allele flips.

We have carefully assessed the consistency at four loci, each defined as the +/- 500 kb region around a trait-associated HC ELE prioritized by the RSS-NET extension for either BMI (Fig.s S11-12) or schizophrenia (Fig.s S13-14). For all four loci, the 𝜆# values are close to zero, the maximum LR values are less than one (i.e., 𝑙𝑜𝑔𝐿𝑅 < *0*), and the expected and observed Z scores

are well aligned across all SNPs. These results confirm the consistency between the GWAS summary statistics and the reference LD matrices at the four prioritized loci, thereby addressing Reviewer #3's comment. We have added the consistency check results as a new **Fig. S16** in the revised **Supplemental Information (Page 23)**. For the convenience of reviewers, we include **Fig. S16 Panel a** below, which demonstrates the consistency check for the specific example (Fig. S11) mentioned in Reviewer #3's comment above.

We thank Reviewer #3 for this comprehensive and insightful comment. In response, we have introduced the following revisions to fully address this comment and improve the manuscript.

- **Page 31, Lines 1389-1399**: "*Since both GWAS datasets were derived from cohorts of EUR ancestry, we supplied the RSS-NET extension with reference LD estimates based on the haplotypes of unrelated individuals with EUR ancestry from Phase 3 of the 1000 Genomes Project (Ref. 34). Prior to the analysis of each GWAS with the RSS-NET extension, we executed a series of quality control procedures to ensure consistency between the GWAS summary statistics and the reference LD estimates (Note S4; Fig. S16).*"
- **Supplemental Information Page 4, Note S4:** Major sources of inconsistencies between the GWAS summary statistics and the reference LD estimates, as well as the QC procedures implemented in this study to address them.
- **Supplemental Information Page 23, Fig. S16**: Consistency between GWAS summary statistics and reference LD estimates at four loci prioritized by HC ELEs associated with either BMI (Fig.s S11-12) or schizophrenia (Fig.s S13-14).

With these new revisions, we have articulated the LD reference panels and the QC procedures employed in the present study, as well as confirmed the consistency between the GWAS summary statistics and the reference LD estimates that underpin our previous results.

(2) The scales of the parameters (prior effect variance) seem to be different in simulations (page 18 of the letter) and in real data (page 19). It would be good to report the results in the same scale.

We agree with Reviewer #3 that the prior effect variance parameters {𝜎*^2^*, 𝜎*^2^*} in both simulation studies and real data analyses should ideally be on the same scale (at least in principle), and we did thoroughly investigate this factor while designing simulation studies for the previous round of revision. However, enforcing the scale of {𝜎*^2^*, 𝜎*^2^*} in simulations to be the same as the very small values in real GWAS is practically infeasible in this study. This is because the sample size of individual-level genotype data that we have access to for the present study (*N*=1458) is much smaller in magnitude than the total sample size underlying the GWAS meta-analysis summary statistics (e.g., *N*=795,640 for BMI). If we were to set the scale of {𝜎*^2^*, 𝜎*^2^*} in simulations as small as that in real GWAS, then we would have to compile a much larger dataset of individual- level genotypes that match the total sample size of the current GWAS meta-analysis, in order to estimate the small variance values with adequate statistical power. For the present study, we do not have access to any other individual-level genotype datasets with sample sizes matching the current GWAS meta-analysis, and therefore we cannot match the scales of {𝜎*^2^*, 𝜎*^2^*} in both simulation studies and real data analyses as Reviewer #3 suggested. That being said, we believe that our extensive simulation studies presented in the previous round of revision have effectively demonstrated the statistical properties and practical utilities of our new approach, which are consistent with the results obtained when applying this approach to real GWAS.

*0*

*0*

*0*

*0*
